# Supplementary material for: Synthesis and Spectral Study of a New Family of 2,5-Diaryltriazoles Having Restricted Rotation of the 5-Aryl Substituent
Source: Molecules. 2020 Jan 23;25(3):480. doi: 10.3390/molecules25030480 (PMC7037111; doi:10.3390/molecules25030480)
Supplement: Supplementary file 1 [file molecules-25-00480-s001.pdf]

Supplementary information

Synthesis and spectral study of new family of 2,5-diaryltriazoles having restricted rotation of 5-aryl substituent

Tsyrenova Biligma[a], Nenajdenko Valentine[a]

[a] *Department of Chemistry, Lomonosov Moscow State University, Leninsky Gory Street, 1, 119234, Moscow, Russia*

22.04.2019 21:59:35

Acquisition Time (sec)	4.0894	***Comment***	5 mm BBO BB-1H/D Z3918/0123	***Date***	01 Feb 2019 13:02:56
Date Stamp	01 Feb 2019 13:02:56				
File Name				***Frequency (MHz)***	400.13
Nucleus	1H	***Number of Transients***	4	***Origin*** spect	***Original Points Count*** 32768
Owner	root	***Points Count***	131072	***Pulse Sequence*** zg30	***Receiver Gain*** 114.00
SW(cyclical) (Hz)	8012.82	***Solvent***	CHLOROFORM-d		***Spectrum Offset (Hz)*** 2395.8254
Sweep Width (Hz)	8012.76	***Temperature (degree C)***	27.000		

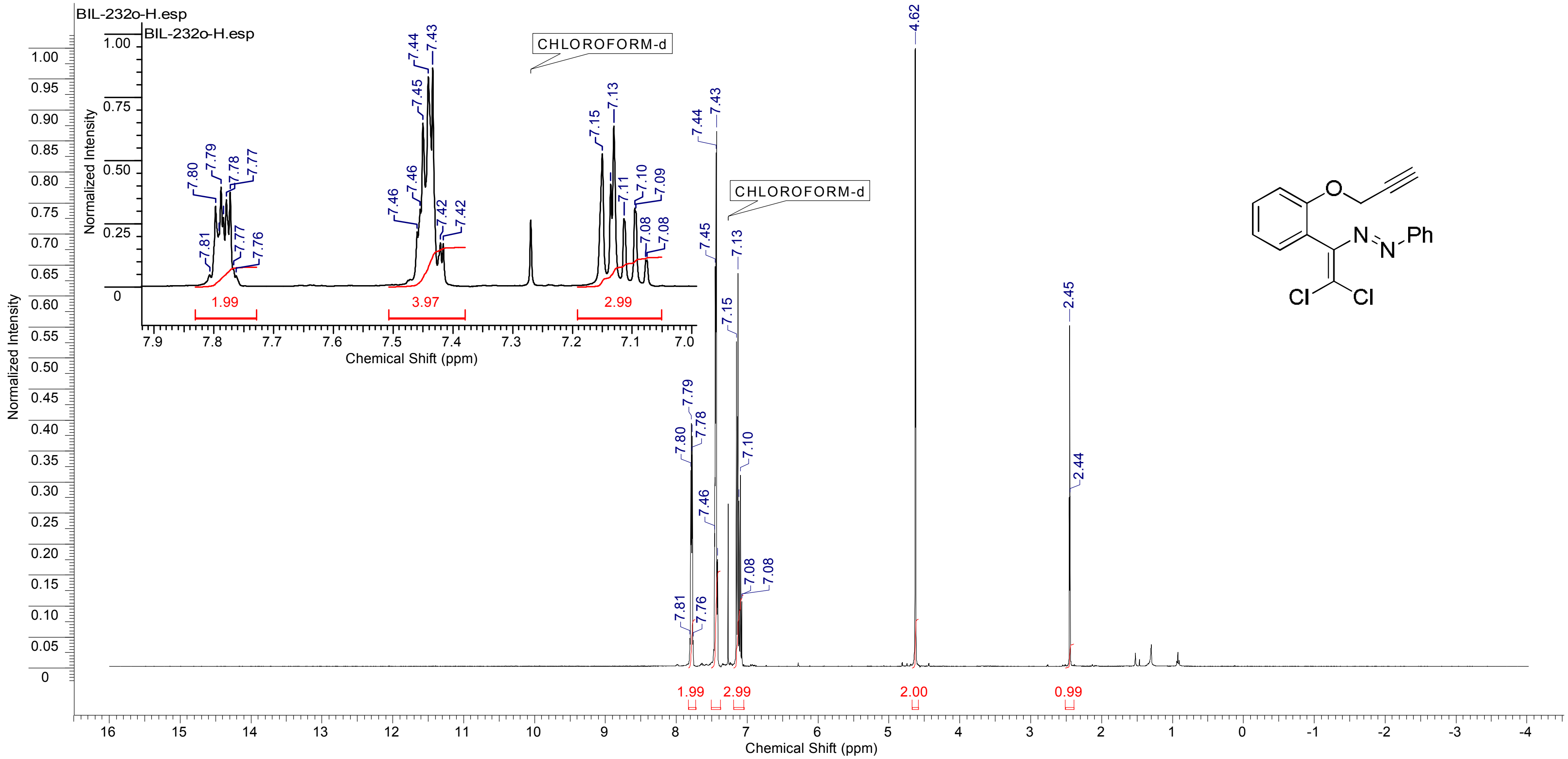

^{1}H NMR spectrum of **2a** (400.1 MHz, $CDCl_3$)

22.04.2019 21:54:14

**Acquisition Time (sec)**	0.6783	**Comment**	5 mm BBO BB-1H/D Z3918/0123			**Date**	01 Feb 2019 13:05:04
**Date Stamp**	01 Feb 2019 13:05:04						
**File Name**						**Frequency (MHz)**	100.61
**Nucleus**	13C	**Number of Transients**	93	**Origin**	spect	**Original Points Count**	16384
**Owner**	root	**Points Count**	131072	**Pulse Sequence**	zgpg30	**Receiver Gain**	13004.00
**SW(cyclical) (Hz)**	24154.59	**Solvent**	CHLOROFORM-d	**Spectrum Offset (Hz)**	11059.6152	**Sweep Width (Hz)**	24154.41
**Temperature (degree C)**	27.000						

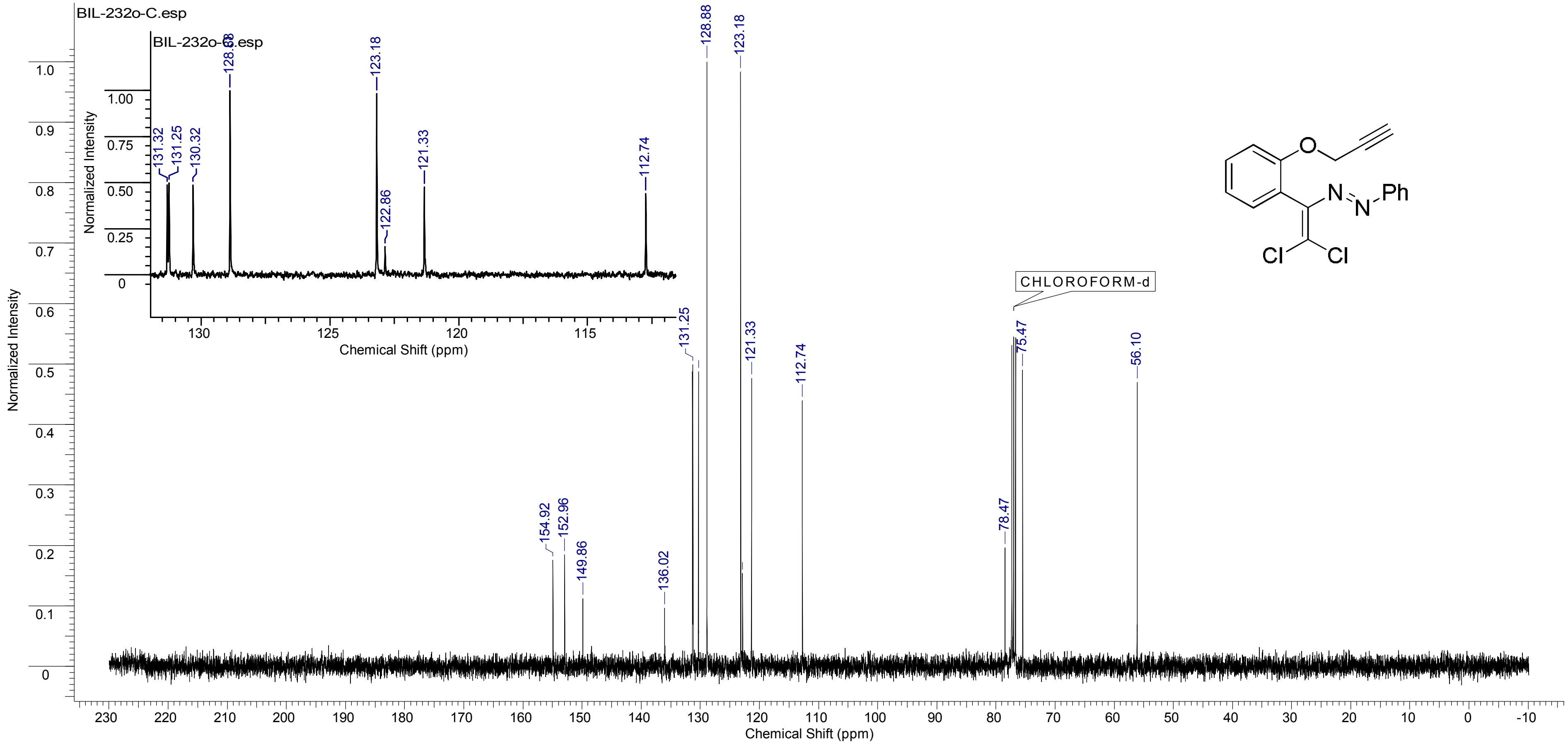

^{13}C NMR spectrum of **2a** (100.6 MHz, $CDCl_3$)

22.04.2019 22:32:15

**Acquisition Time (sec)**	2.5559	**Comment**	5 mm BBO BB-1H/D Z3918/0123			**Date**	08 Jun 2018 12:18:08
**Date Stamp**	08 Jun 2018 12:18:08			**File Name**		**Frequency (MHz)**	400.13
**Nucleus**	1H	**Number of Transients**	4	**Origin**	spect	**Original Points Count**	16384
						**Owner**	root
**Points Count**	65536	**Pulse Sequence**	zg30	**Receiver Gain**	161.30	**SW(cyclical) (Hz)**	6410.26
						**Solvent**	CHLOROFORM-d
**Spectrum Offset (Hz)**	2595.7979	**Sweep Width (Hz)**	6410.16	**Temperature (degree C)**	27.000		

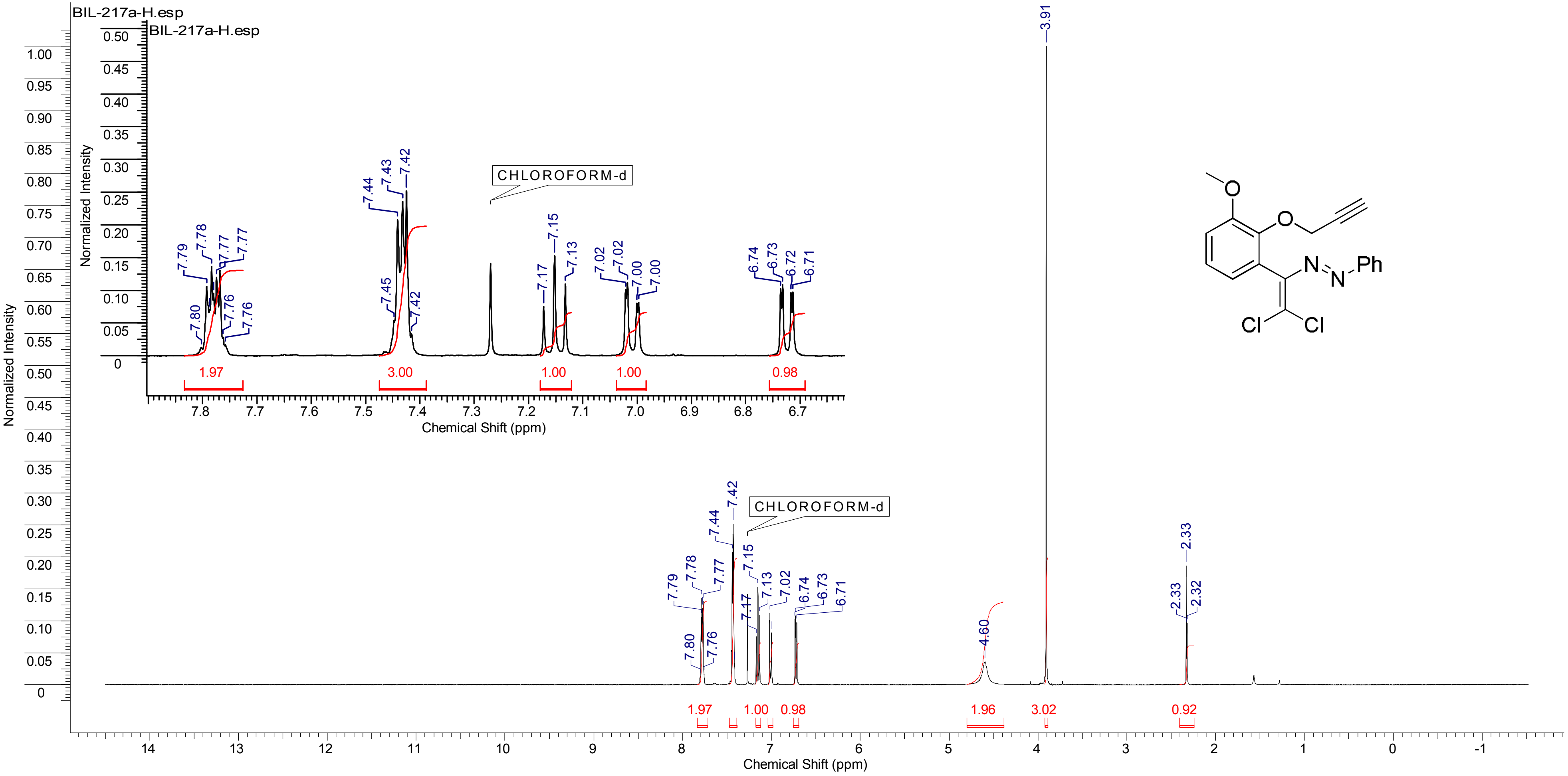

^{1}H NMR spectrum of **2b** (400.1 MHz, $CDCl_3$)

22.04.2019 22:33:14

Acquisition Time (sec)	0.4999	***Comment***	5 mm BBO BB-1H/D Z3918/0123			***Date***	07 Jun 2018 09:46:40		
Date Stamp	07 Jun 2018 09:46:40			***File Name***				***Frequency (MHz)***	100.61
Nucleus	13C	***Number of Transients***	262	***Origin***	spect	***Original Points Count***	12076	***Owner***	root
Points Count	65536	***Pulse Sequence***	zgpg30	***Receiver Gain***	13004.00	***SW(cyclical) (Hz)***	24154.59		
Solvent	CHLOROFORM-d			***Spectrum Offset (Hz)***	11061.1816	***Sweep Width (Hz)***	24154.22	***Temperature (degree C)***	27.000

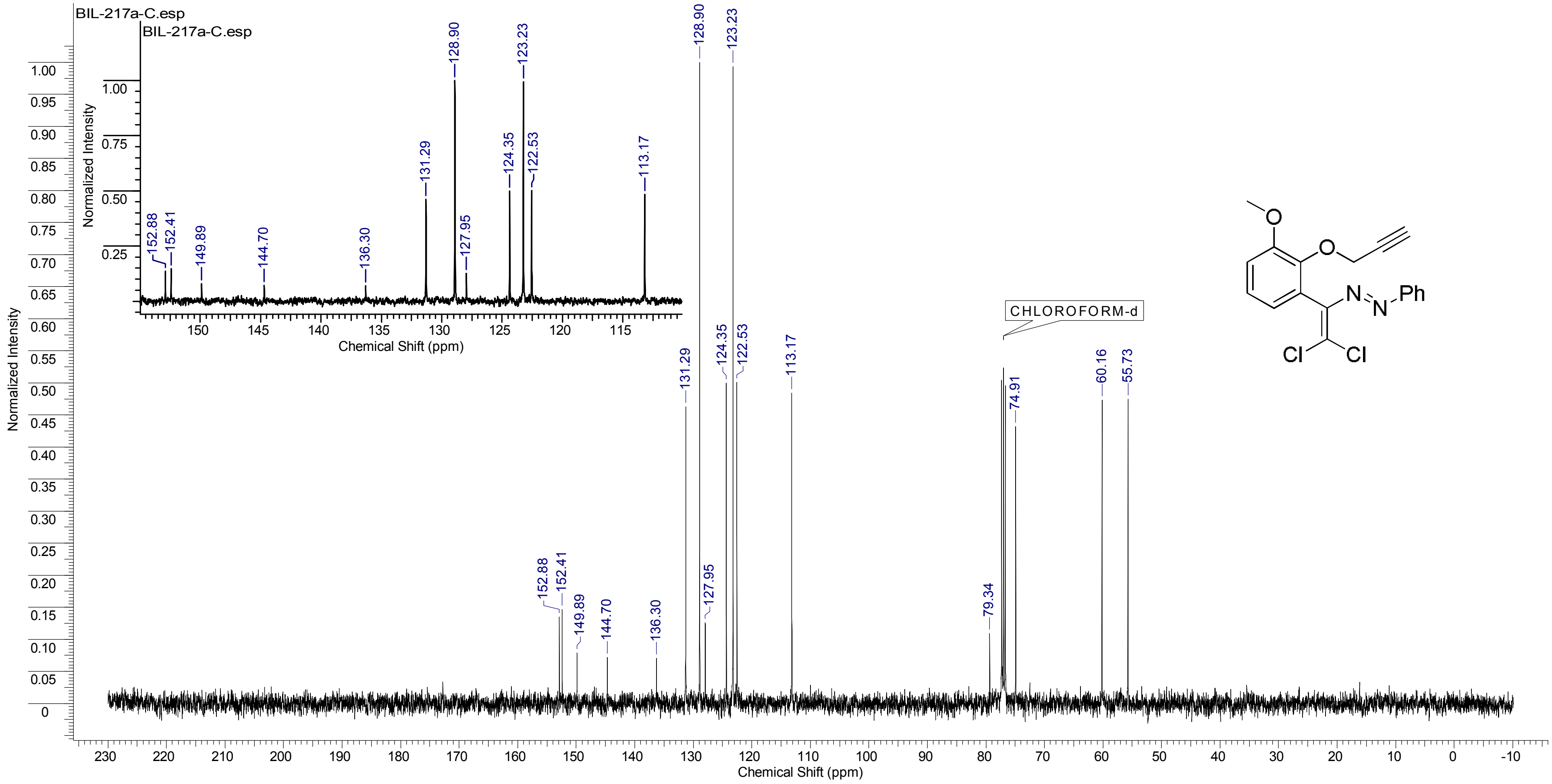

^{13}C NMR spectrum of **2b** (100.6 MHz, $CDCl_3$)

26.04.2019 0:33:15

Acquisition Time (sec)	4.0894	***Comment***	Imported from UXNMR.			***Date***	21 Feb 2019 11:44:08
File Name						***Frequency (MHz)***	400.13
Nucleus	1H	***Number of Transients***	4	***Original Points Count***	32768	***Points Count***	131072
Pulse Sequence	zg30	***Solvent***	CHLOROFORM-d			***Spectrum Offset (Hz)***	2396.2495
Sweep Width (Hz)	8012.82	***Temperature (degree C)***	27.000				

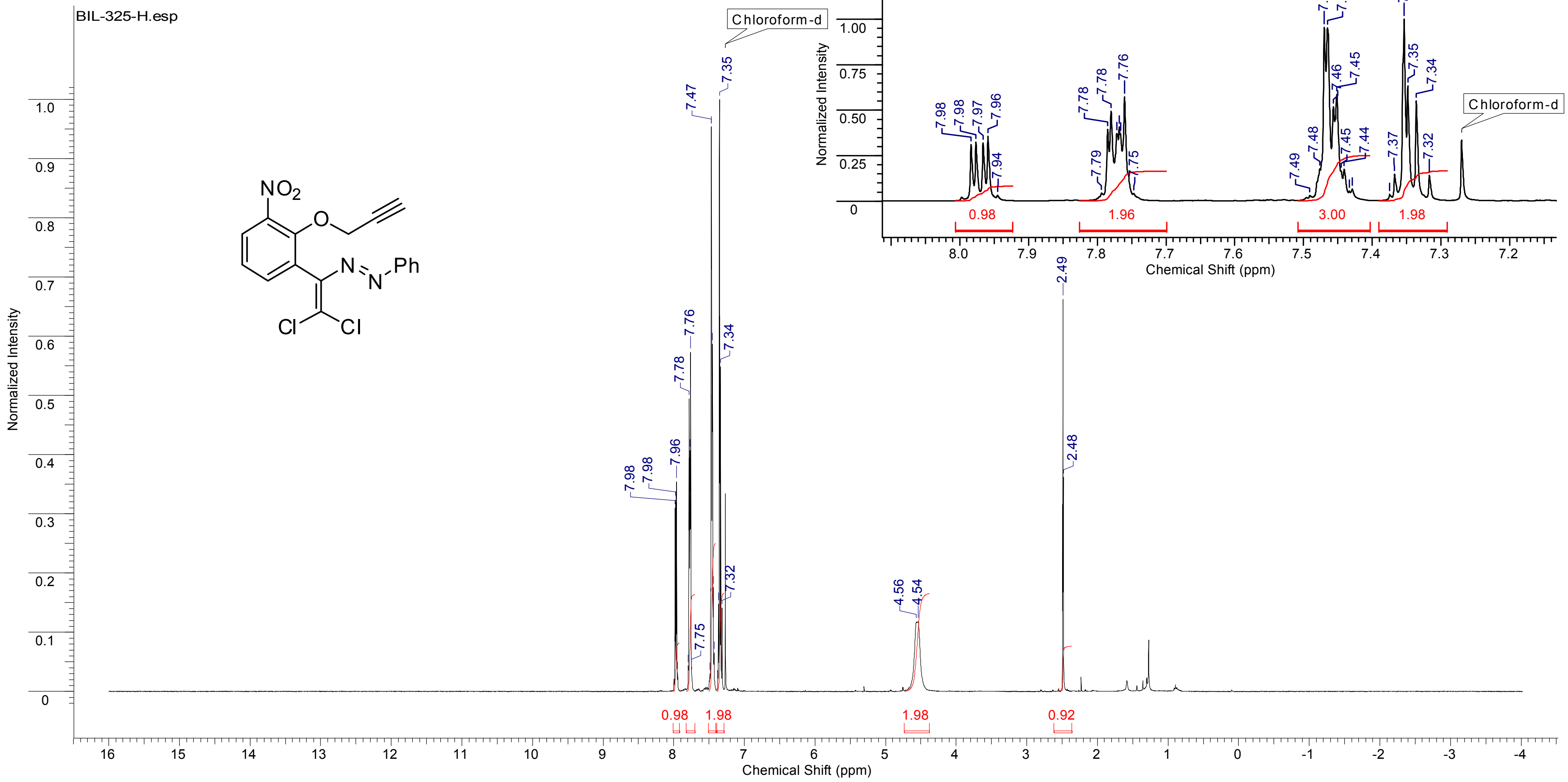

^{1}H NMR spectrum of **2c** (400.1 MHz, $CDCl_3$)

26.04.2019 0:41:44

Acquisition Time (sec)	0.6783	***Comment***	Imported from UXNMR.			***Date***	21 Feb 2019 11:50:38
File Name						***Frequency (MHz)***	100.61
Nucleus	13C	***Number of Transients***	143	***Original Points Count***	16384	***Points Count***	131072
Pulse Sequence	zgpg30	***Solvent***	CHLOROFORM-d			***Spectrum Offset (Hz)***	11060.7461
Sweep Width (Hz)	24154.59	***Temperature (degree C)***	27.000				

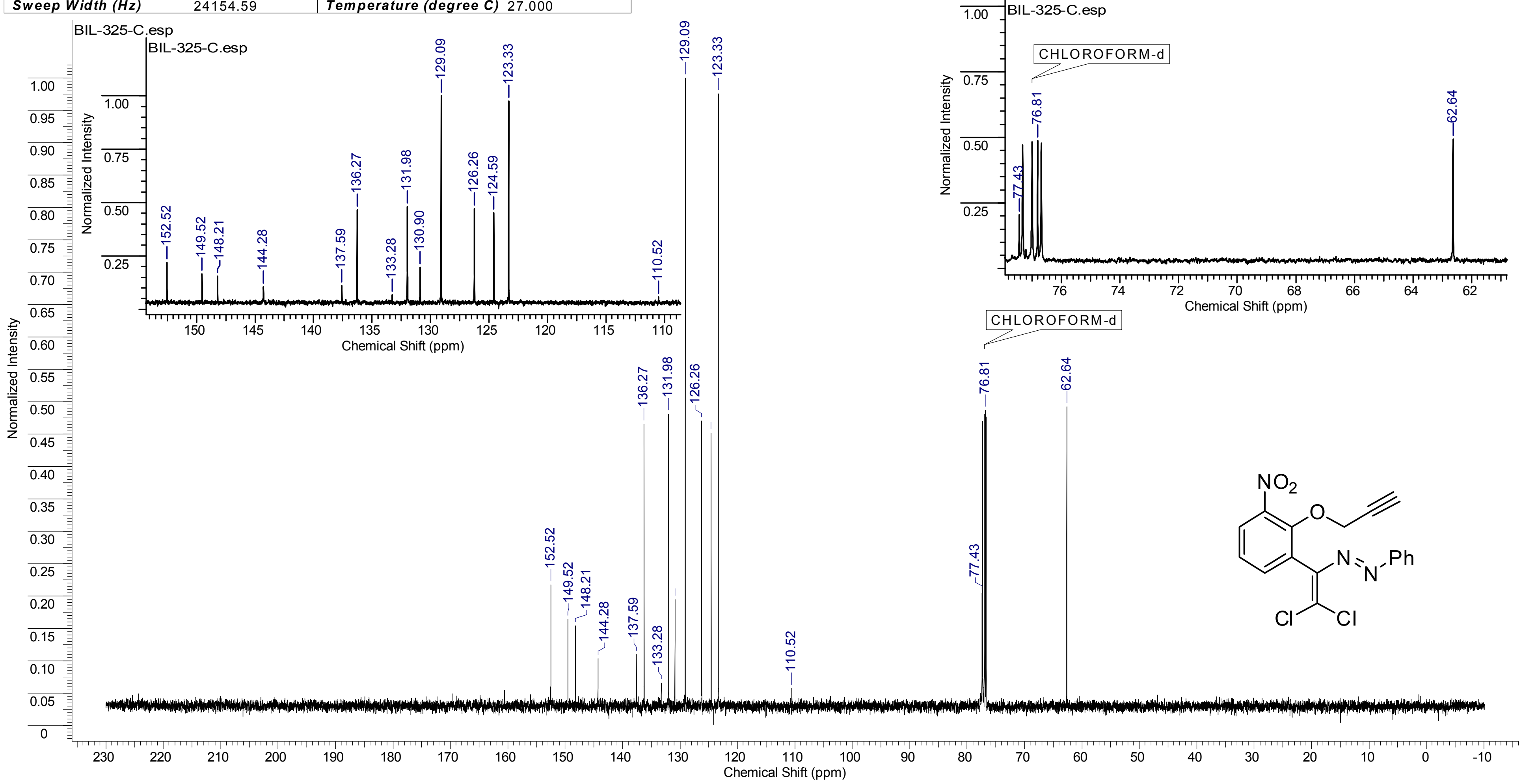

^{13}C NMR spectrum of **2c** (100.6 MHz, $CDCl_3$)

23.04.2019 0:19:59

**Acquisition Time (sec)**	4.0894	**Comment**	5 mm Dual 13C/1H Z3756/0200			**Date**	19 Feb 2019 11:46:08
**Date Stamp**	19 Feb 2019 11:46:08						
**File Name**						**Frequency (MHz)**	400.13
**Nucleus**	1H	**Number of Transients**	6	**Origin**	spect	**Original Points Count**	32768
**Owner**	root	**Points Count**	131072	**Pulse Sequence**	zg30	**Receiver Gain**	114.00
**SW(cyclical) (Hz)**	8012.82	**Solvent**	CHLOROFORM-d			**Spectrum Offset (Hz)**	2396.6816
**Sweep Width (Hz)**	8012.76	**Temperature (degree C)**	27.000				

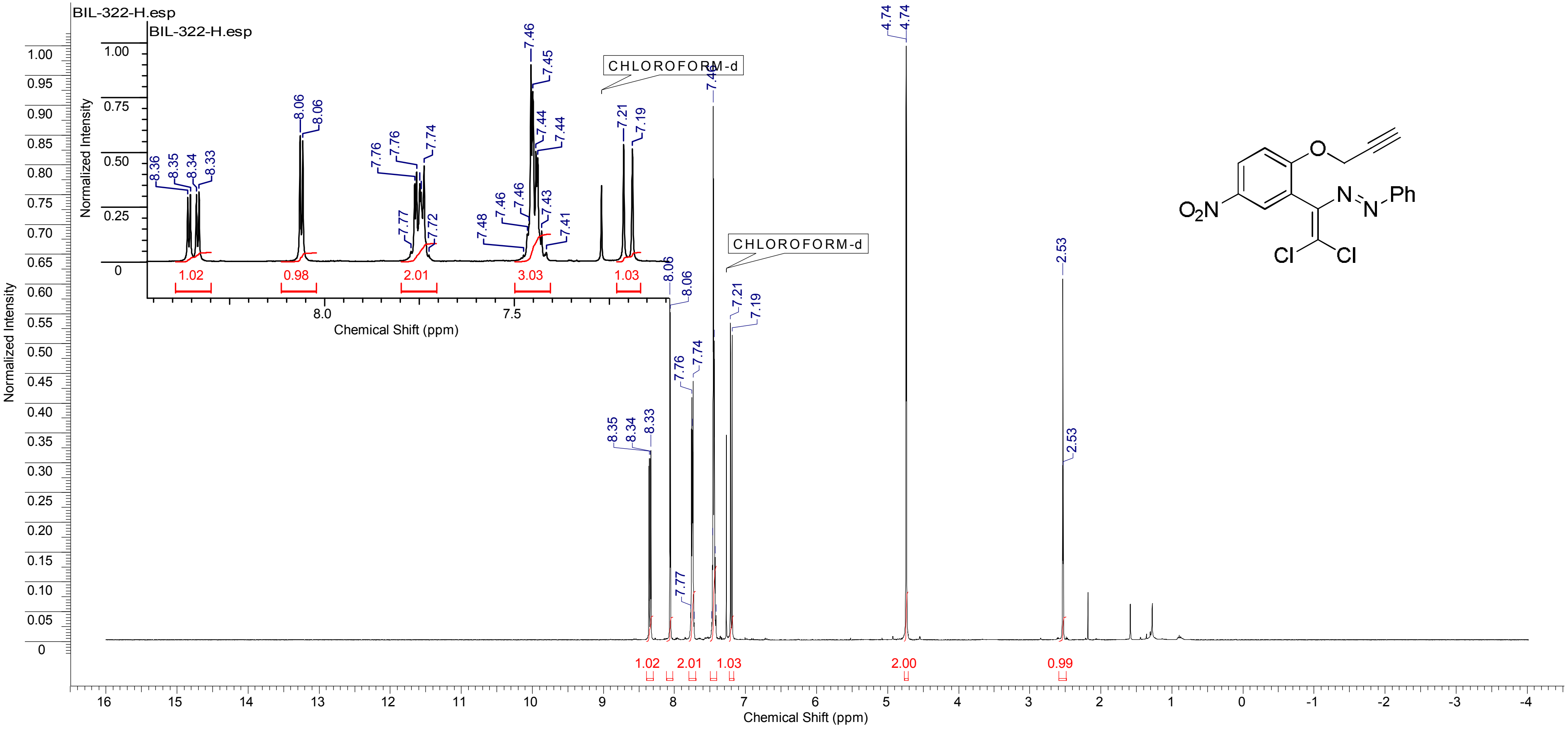

^{1}H NMR spectrum of **2d** (400.1 MHz, $CDCl_3$)

23.04.2019 0:20:59

**Acquisition Time (sec)**	0.6783	**Comment**	5 mm Dual 13C/1H Z3756/0200			**Date**	19 Feb 2019 11:46:08
**Date Stamp**	19 Feb 2019 11:46:08						
**File Name**						**Frequency (MHz)**	100.61
**Nucleus**	13C	**Number of Transients**	97	**Origin**	spect	**Original Points Count**	16384
**Owner**	root	**Points Count**	131072	**Pulse Sequence**	zgpg30	**Receiver Gain**	14596.50
**SW(cyclical) (Hz)**	24154.59	**Solvent**	CHLOROFORM-d	**Spectrum Offset (Hz)**	11077.1221	**Sweep Width (Hz)**	24154.41
**Temperature (degree C)**	27.000						

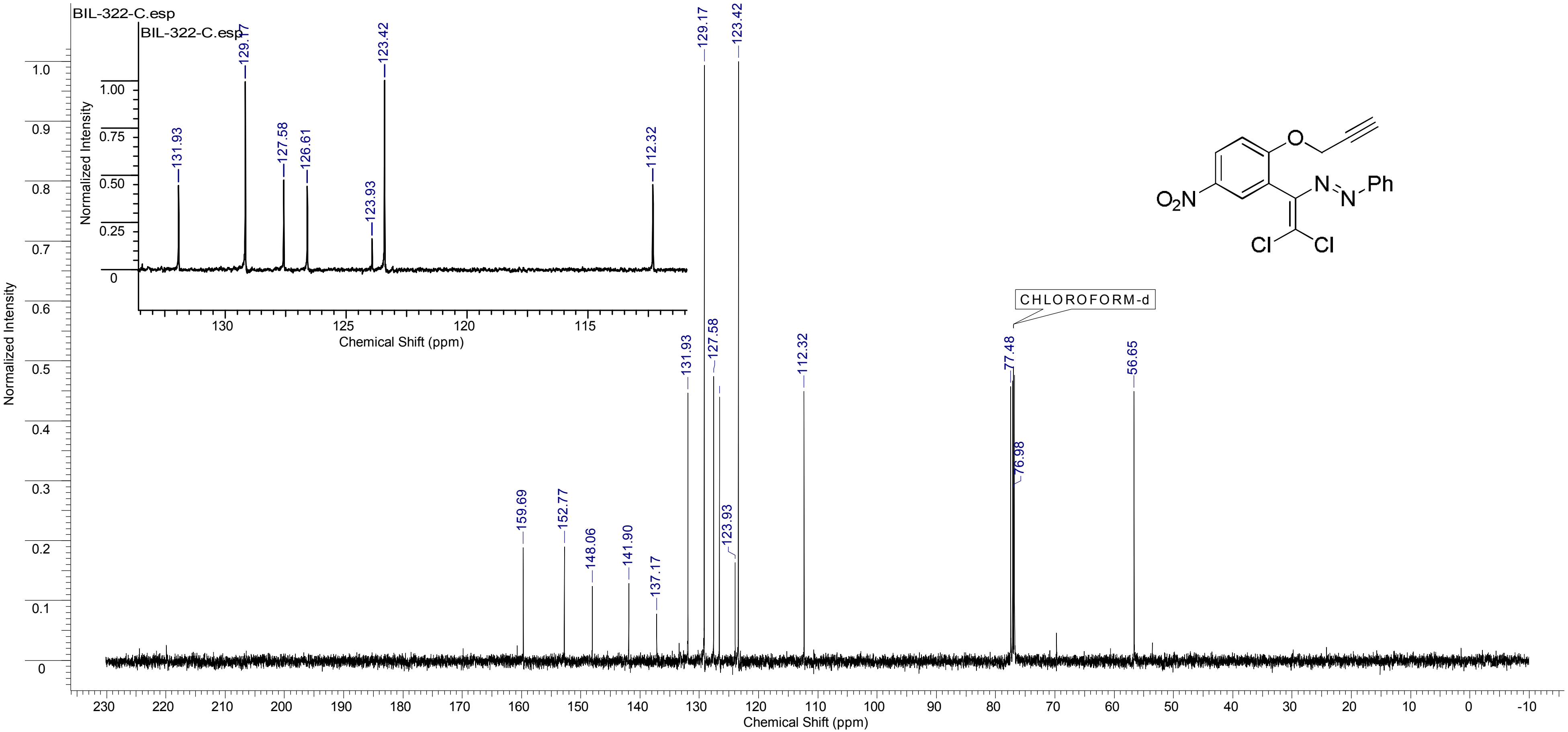

^{13}C NMR spectrum of **2d** (100.6 MHz, $CDCl_3$)

22.04.2019 23:41:47

Acquisition Time (sec)	2.5559	***Comment***	Imported from UXNMR.			***Date***	28 Jan 2019 15:39:06		
File Name						***Frequency (MHz)***	400.13	***Nucleus***	1H
Number of Transients	4	***Original Points Count***	16384	***Points Count***	65536	***Pulse Sequence***	zg30	***Solvent***	DMSO-d6
Spectrum Offset (Hz)	2595.9883	***Sweep Width (Hz)***	6410.26	***Temperature (degree C)***	27.000				

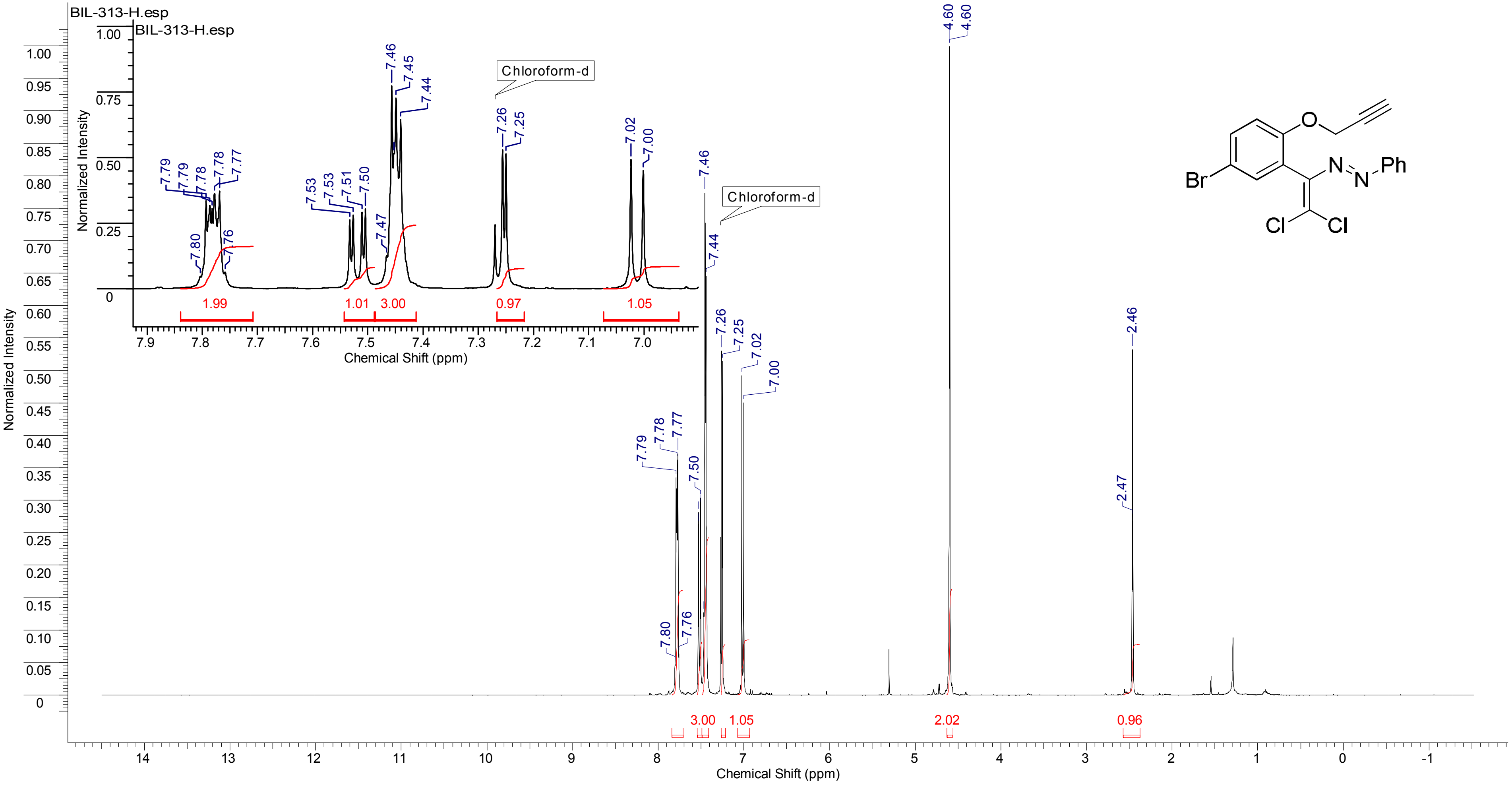

^{1}H NMR spectrum of **2e** (400.1 MHz, $CDCl_3$)

22.04.2019 23:42:33

Acquisition Time (sec)	0.6783	***Comment***	Imported from UXNMR.			***Date***	28 Jan 2019 15:40:56
File Name						***Frequency (MHz)***	100.61
Nucleus	13C	***Number of Transients***	23	***Original Points Count***	16384	***Points Count***	131072
Pulse Sequence	zgpg30	***Solvent***	CHLOROFORM-D	***Spectrum Offset (Hz)***	11058.7188	***Sweep Width (Hz)***	24154.59
Temperature (degree C)	27.000						

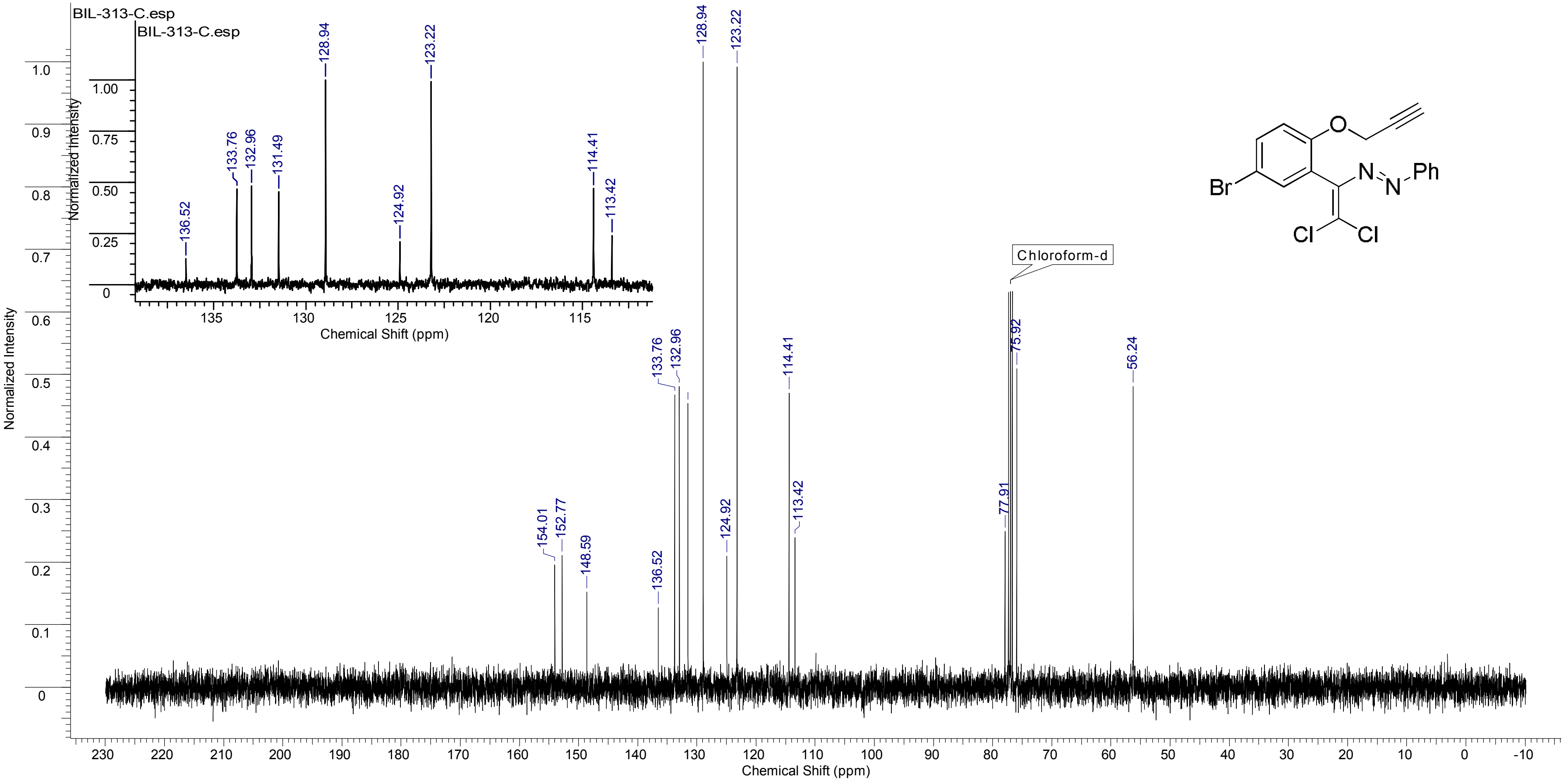

^{13}C NMR spectrum of **2e** (100.6 MHz, $CDCl_3$)

24.06.2019 19:29:34

**Acquisition Time (sec)**	4.0894	**Comment**	5 mm BBO BB-1H/D Z3918/0123			**Date**	30 Apr 2019 12:39:28		
**Date Stamp**	30 Apr 2019 12:39:28			**File Name**					
**Frequency (MHz)**	400.13	**Nucleus**	1H	**Number of Transients**	4	**Origin**	spect	**Original Points Count**	32768
**Owner**	root	**Points Count**	131072	**Pulse Sequence**	zg30	**Receiver Gain**	128.00	**SW(cyclical) (Hz)**	8012.82
**Solvent**	CHLOROFORM-d			**Spectrum Offset (Hz)**	2395.7644	**Sweep Width (Hz)**	8012.76	**Temperature (degree C)**	27.000

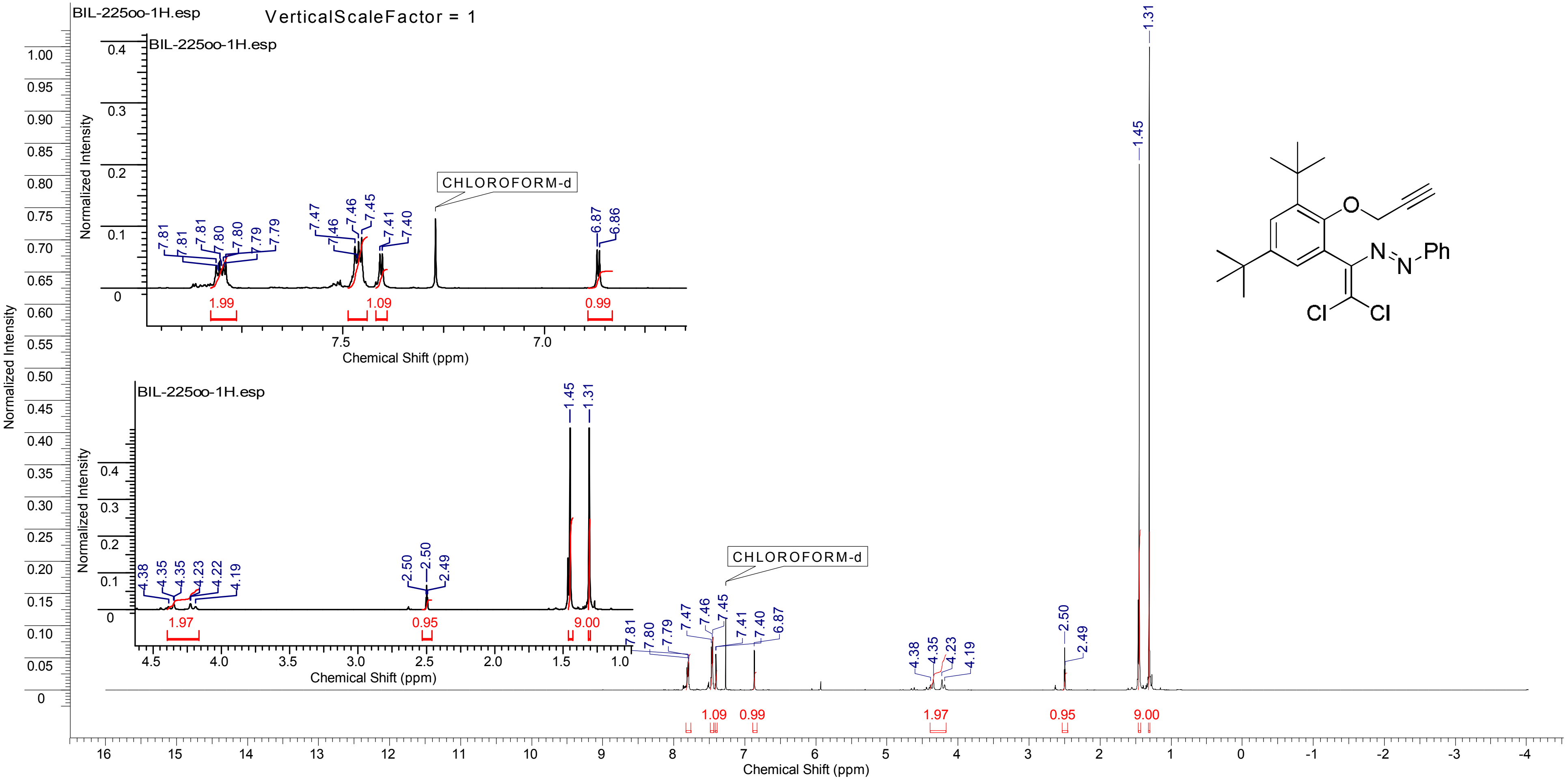

^{1}H NMR spectrum of **2f** (400.1 MHz, $CDCl_3$)

24.06.2019 20:26:57

Acquisition Time (sec)	0.6783	***Comment***	5 mm BBO BB-1H/D Z3918/0123			***Date***	30 Apr 2019 12:39:28
Date Stamp	30 Apr 2019 12:39:28			***File Name***			
Frequency (MHz)	100.61	***Nucleus***	13C	***Number of Transients***	145	***Origin***	spect
Original Points Count	16384	***Owner***	root	***Points Count***	131072	***Pulse Sequence***	zgpg30
Receiver Gain	13004.00	***SW(cyclical) (Hz)***	24154.59	***Solvent***	CHLOROFORM-d		
Spectrum Offset (Hz)	11063.6699	***Sweep Width (Hz)***	24154.41	***Temperature (degree C)***	27.000		

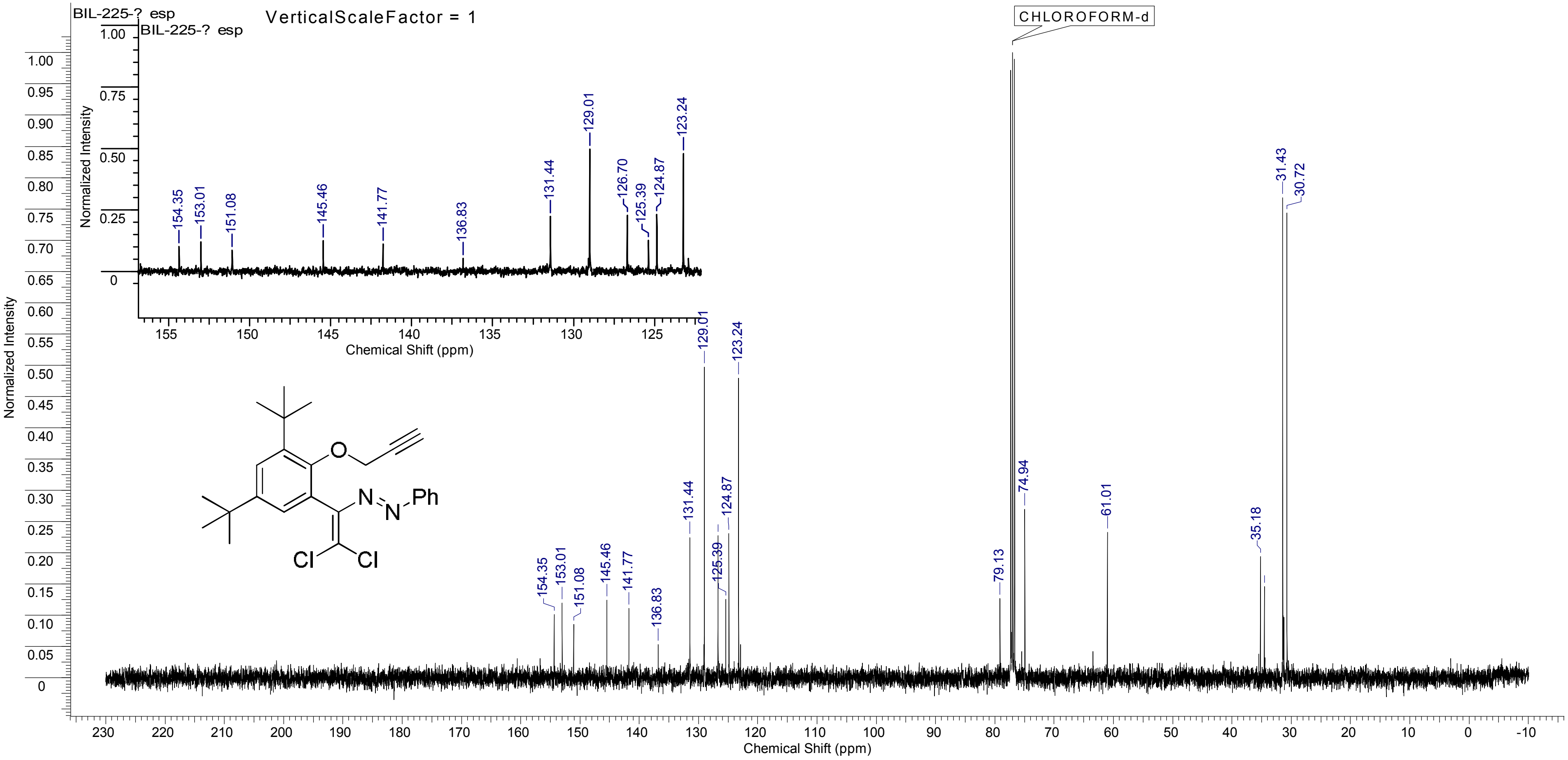

^{13}C NMR spectrum of **2f** (100.6 MHz, $CDCl_3$)

12.05.2019 15:00:32

**Acquisition Time (sec)**	2.5559	**Comment**	5 mm BBO BB-1H/D Z3918/0123			**Date**	22 Oct 2018 13:02:56
**Date Stamp**	22 Oct 2018 13:02:56						
**File Name**						**Frequency (MHz)**	400.13
**Nucleus**	1H	**Number of Transients**	4	**Origin**	spect	**Original Points Count**	16384
**Owner**	root	**Points Count**	65536	**Pulse Sequence**	zg30	**Receiver Gain**	181.00
**SW(cyclical) (Hz)**	6410.26	**Solvent**	CHLOROFORM-d			**Spectrum Offset (Hz)**	2595.7979
**Sweep Width (Hz)**	6410.16	**Temperature (degree C)**	27.000				

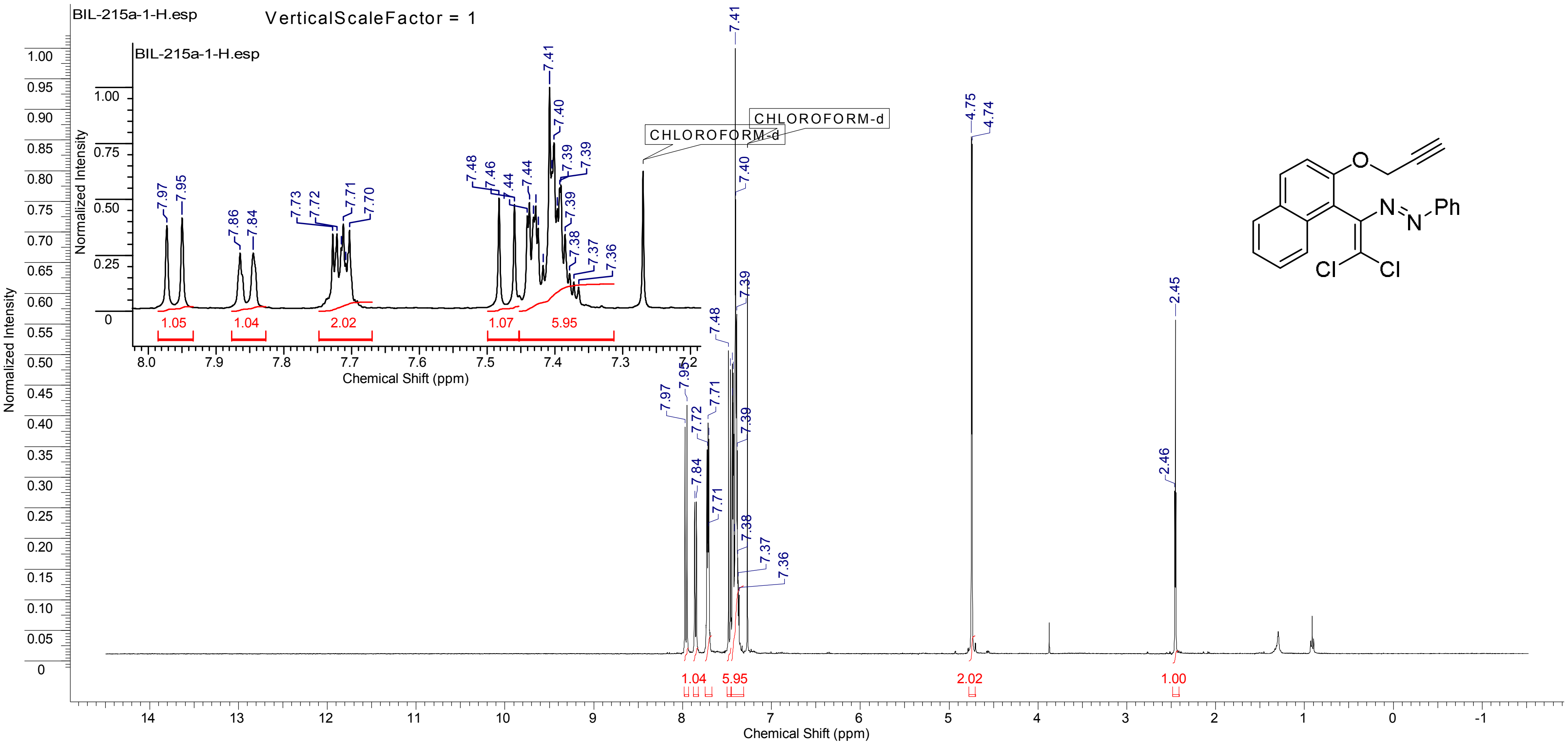

^{1}H NMR spectrum of **2g** (400.1 MHz, $CDCl_3$)

12.05.2019 15:05:34

**Acquisition Time (sec)**	0.4999	**Comment**	5 mm BBO BB-1H/D Z3918/0123			**Date**	24 Oct 2018 15:00:16
**Date Stamp**	24 Oct 2018 15:00:16						
**File Name**						**Frequency (MHz)**	100.61
**Nucleus**	13C	**Number of Transients**	320	**Origin**	spect	**Original Points Count**	12076
**Owner**	root	**Points Count**	65536	**Pulse Sequence**	zgpg30	**Receiver Gain**	13004.00
**SW(cyclical) (Hz)**	24154.59	**Solvent**	CHLOROFORM-d	**Spectrum Offset (Hz)**	11061.5498	**Sweep Width (Hz)**	24154.22
**Temperature (degree C)**	27.000						

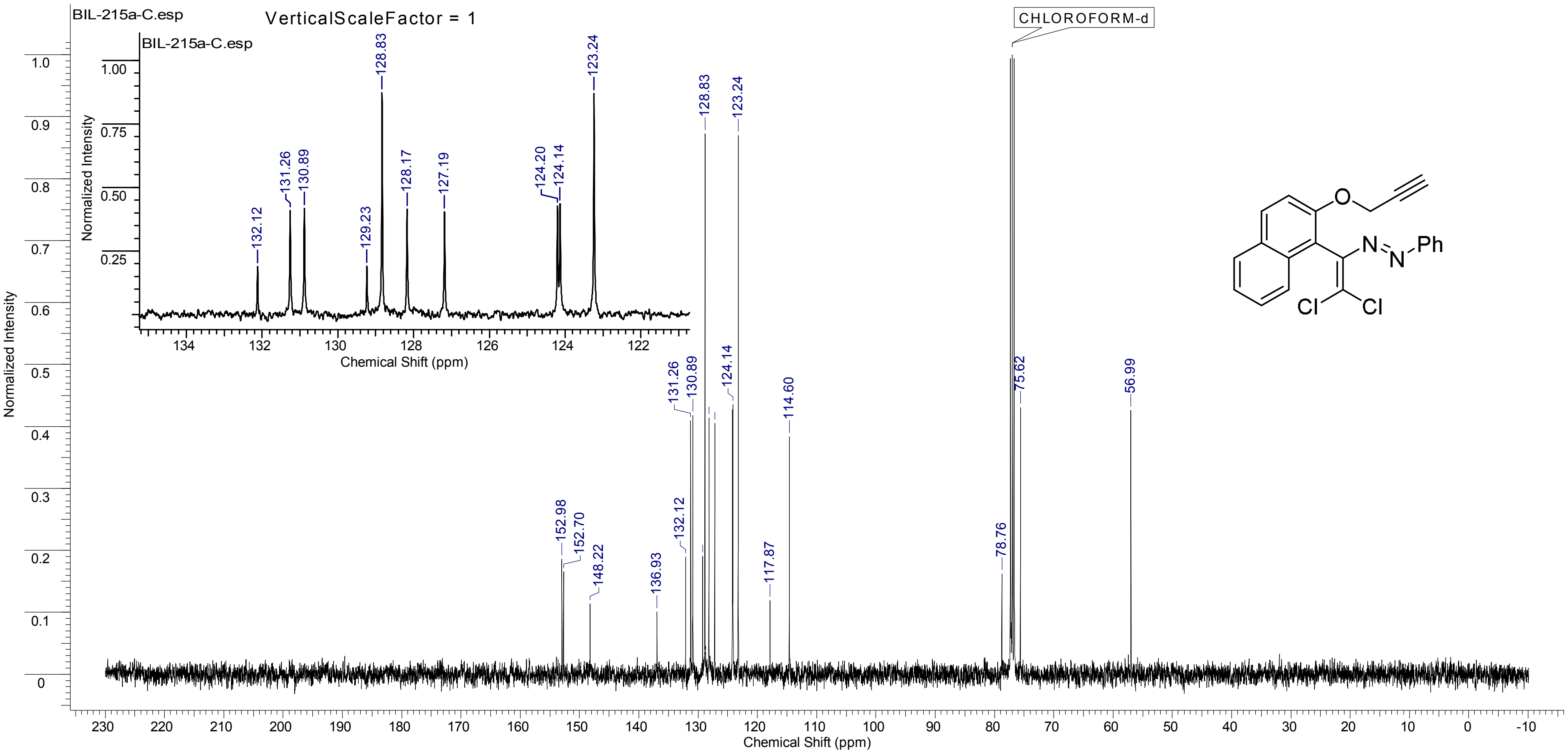

^{13}C NMR spectrum of **2g** (100.6 MHz, $CDCl_3$)

20.11.2019 11:04:41

Acquisition Time (sec)	2.5559	***Comment***	5 mm BBO BB-1H/D Z3918/0123			***Date***	26 Oct 2018 12:39:28
Date Stamp	26 Oct 2018 12:39:28						
File Name						***Frequency (MHz)***	400.13
Nucleus	1H	***Number of Transients***	5	***Origin***	spect	***Original Points Count***	16384
Owner	root	***Points Count***	65536	***Pulse Sequence***	zg30	***Receiver Gain***	114.00
SW(cyclical) (Hz)	6410.26	***Solvent***	CHLOROFORM-d			***Spectrum Offset (Hz)***	2595.7979
Sweep Width (Hz)	6410.16	***Temperature (degree C)***	27.000				

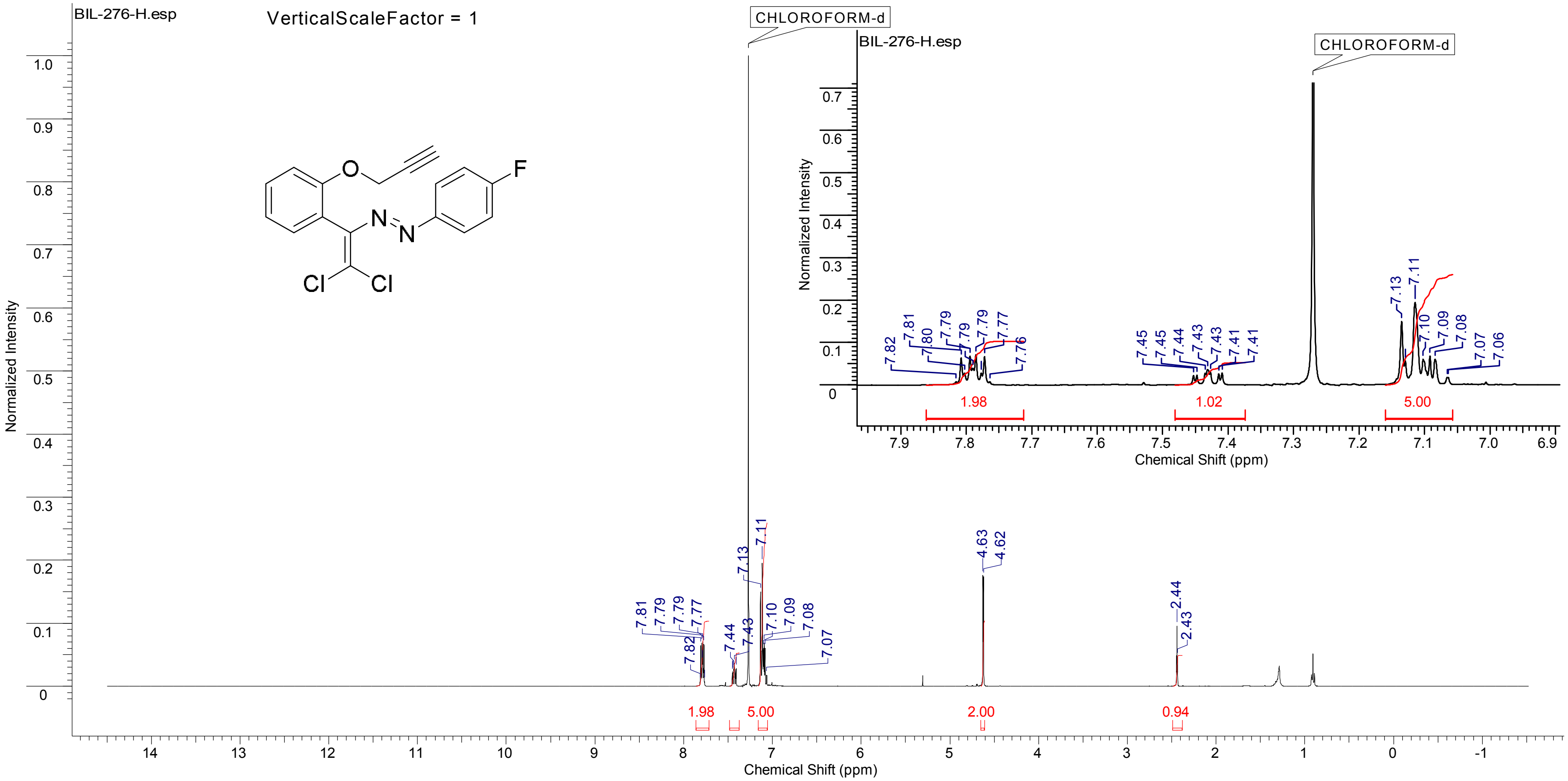

^{1}H NMR spectrum of **2h** (400.1 MHz, $CDCl_3$)

12.05.2019 15:55:13

**Acquisition Time (sec)**	0.4999	**Comment**	5 mm BBO BB-1H/D Z3918/0123			**Date**	26 Oct 2018 12:41:36
**Date Stamp**	26 Oct 2018 12:41:36						
**File Name**						**Frequency (MHz)**	100.61
**Nucleus**	13C	**Number of Transients**	116	**Origin**	spect	**Original Points Count**	12076
**Owner**	root	**Points Count**	65536	**Pulse Sequence**	zgpg30	**Receiver Gain**	8192.00
**SW(cyclical) (Hz)**	24154.59	**Solvent**	CHLOROFORM-d	**Spectrum Offset (Hz)**	11061.9189	**Sweep Width (Hz)**	24154.22
**Temperature (degree C)**	27.000						

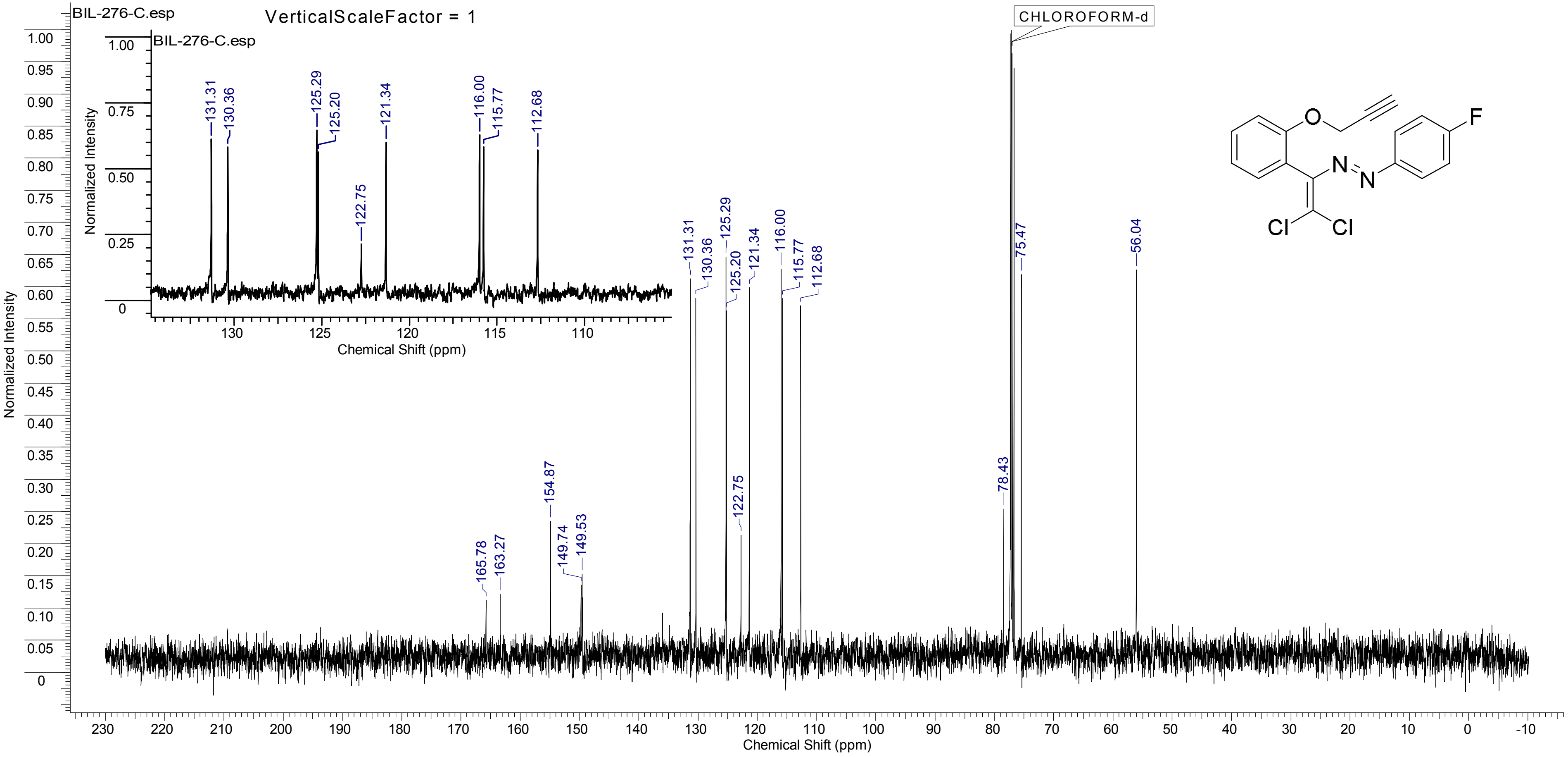

^{13}C NMR spectrum of **2h** (100.6 MHz, $CDCl_3$)

14.05.2019 12:27:41

Acquisition Time (sec)	4.0894	***Comment***	5 mm BBO BB-1H/D Z3918/0123			***Date***	31 Oct 2018 14:30:24
Date Stamp	31 Oct 2018 14:30:24						
File Name						***Frequency (MHz)***	400.13
Nucleus	1H	***Number of Transients***	5	***Origin***	spect	***Original Points Count***	32768
Owner	root	***Points Count***	131072	***Pulse Sequence***	zg30	***Receiver Gain***	228.10
SW(cyclical) (Hz)	8012.82	***Solvent***	CHLOROFORM-d			***Spectrum Offset (Hz)***	2395.6421
Sweep Width (Hz)	8012.76	***Temperature (degree C)***	27.000				

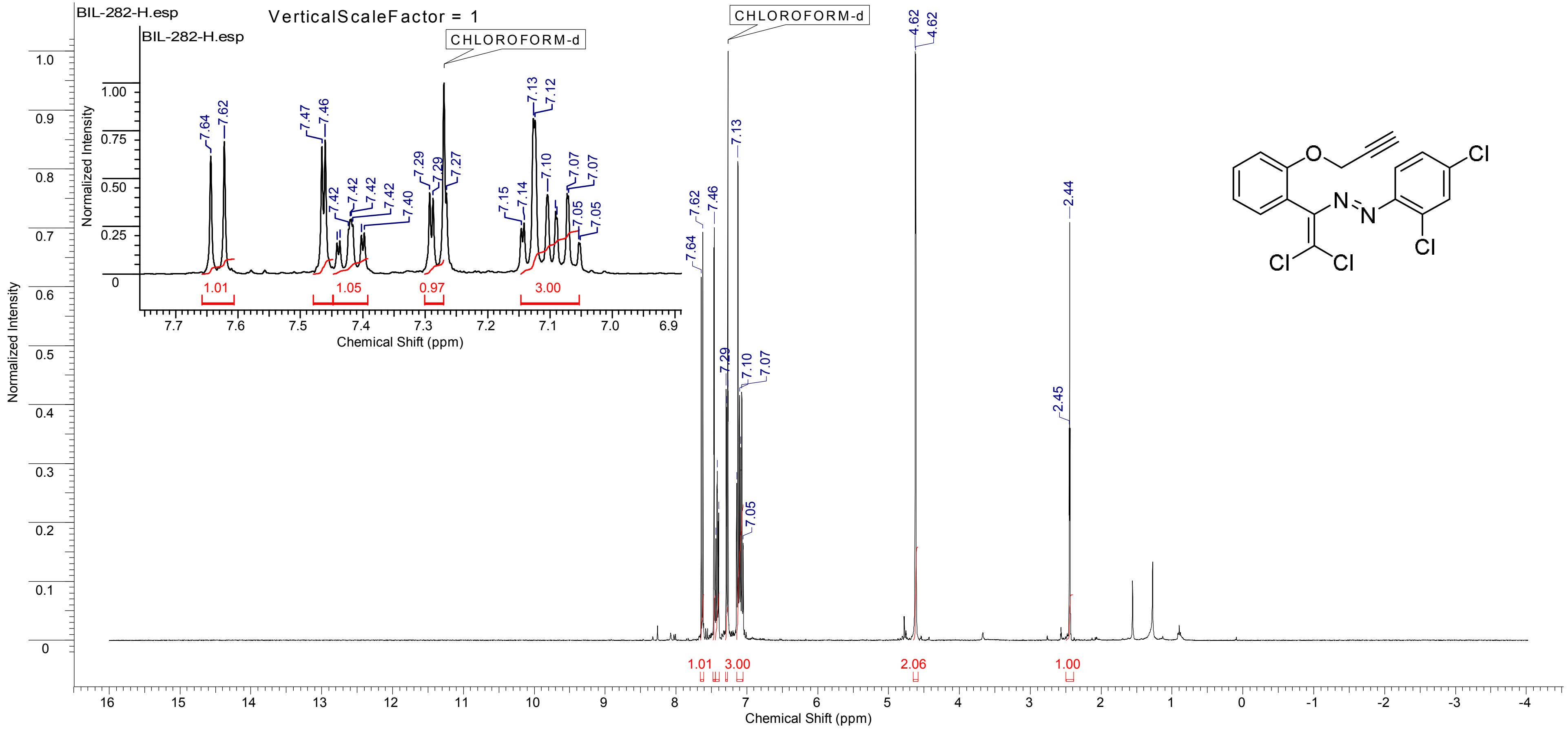

^{1}H NMR spectrum of **2i** (400.1 MHz, $CDCl_3$)

14.05.2019 12:28:20

Acquisition Time (sec)	0.4999	***Comment***	5 mm BBO BB-1H/D Z3918/0123			***Date***	31 Oct 2018 14:32:32
Date Stamp	31 Oct 2018 14:32:32						
File Name						***Frequency (MHz)***	100.61
Nucleus	13C	***Number of Transients***	92	***Origin***	spect	***Original Points Count***	12076
Owner	root	***Points Count***	65536	***Pulse Sequence***	zgpg30	***Receiver Gain***	8192.00
SW(cyclical) (Hz)	24154.59	***Solvent***	CHLOROFORM-d	***Spectrum Offset (Hz)***	11062.6563	***Sweep Width (Hz)***	24154.22
Temperature (degree C)	27.000						

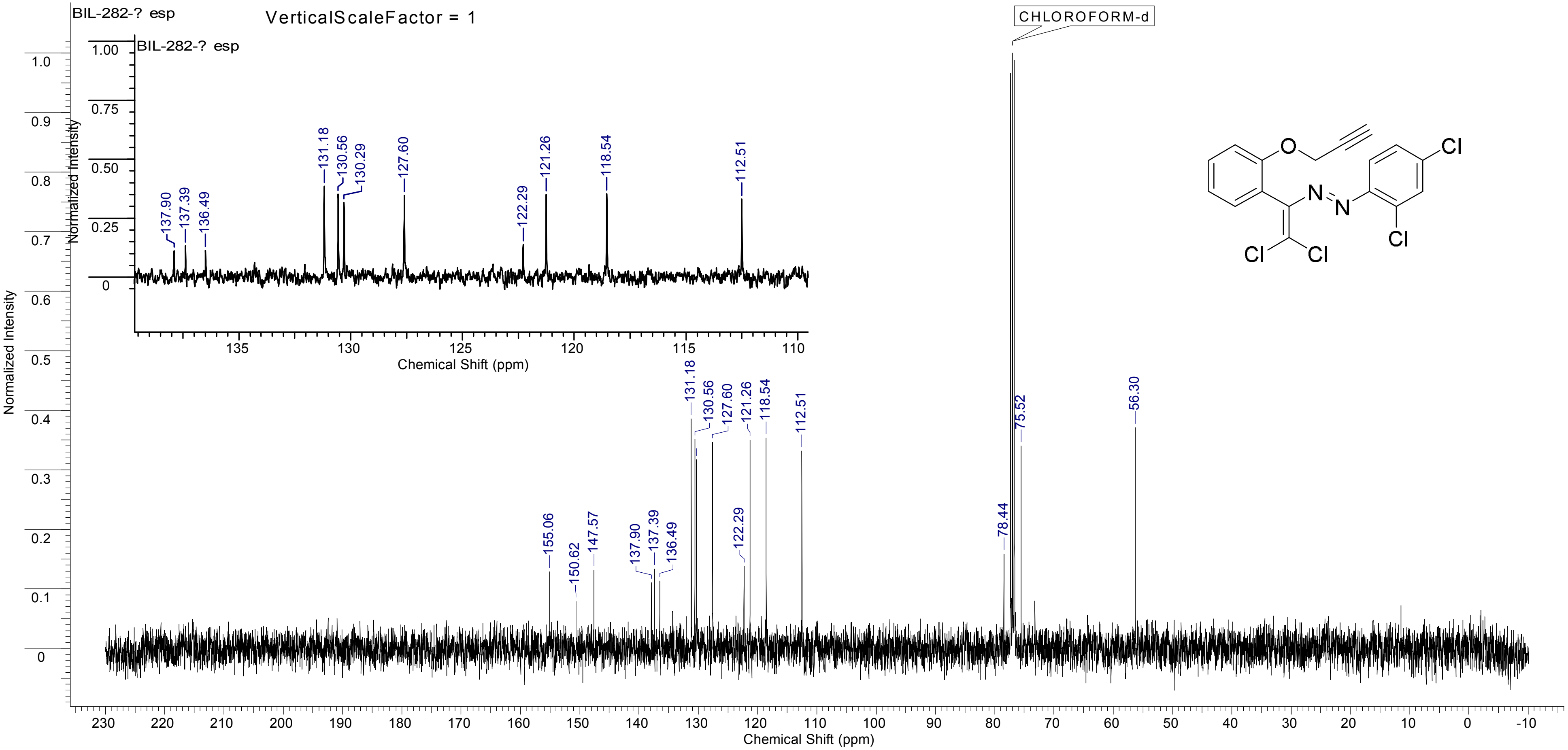

^{13}C NMR spectrum of **2i** (100.6 MHz, $CDCl_3$)

14.05.2019 18:37:09

Acquisition Time (sec)	4.0894	***Comment***	5 mm BBO BB-1H/D Z3918/0123			***Date***	07 Feb 2019 08:14:56
Date Stamp	07 Feb 2019 08:14:56						
File Name						***Frequency (MHz)***	400.13
Nucleus	1H	***Number of Transients***	4	***Origin***	spect	***Original Points Count***	32768
Owner	root	***Points Count***	131072	***Pulse Sequence***	zg30	***Receiver Gain***	362.00
SW(cyclical) (Hz)	8012.82	***Solvent***	CHLOROFORM-d			***Spectrum Offset (Hz)***	2395.7031
Sweep Width (Hz)	8012.76	***Temperature (degree C)***	27.000				

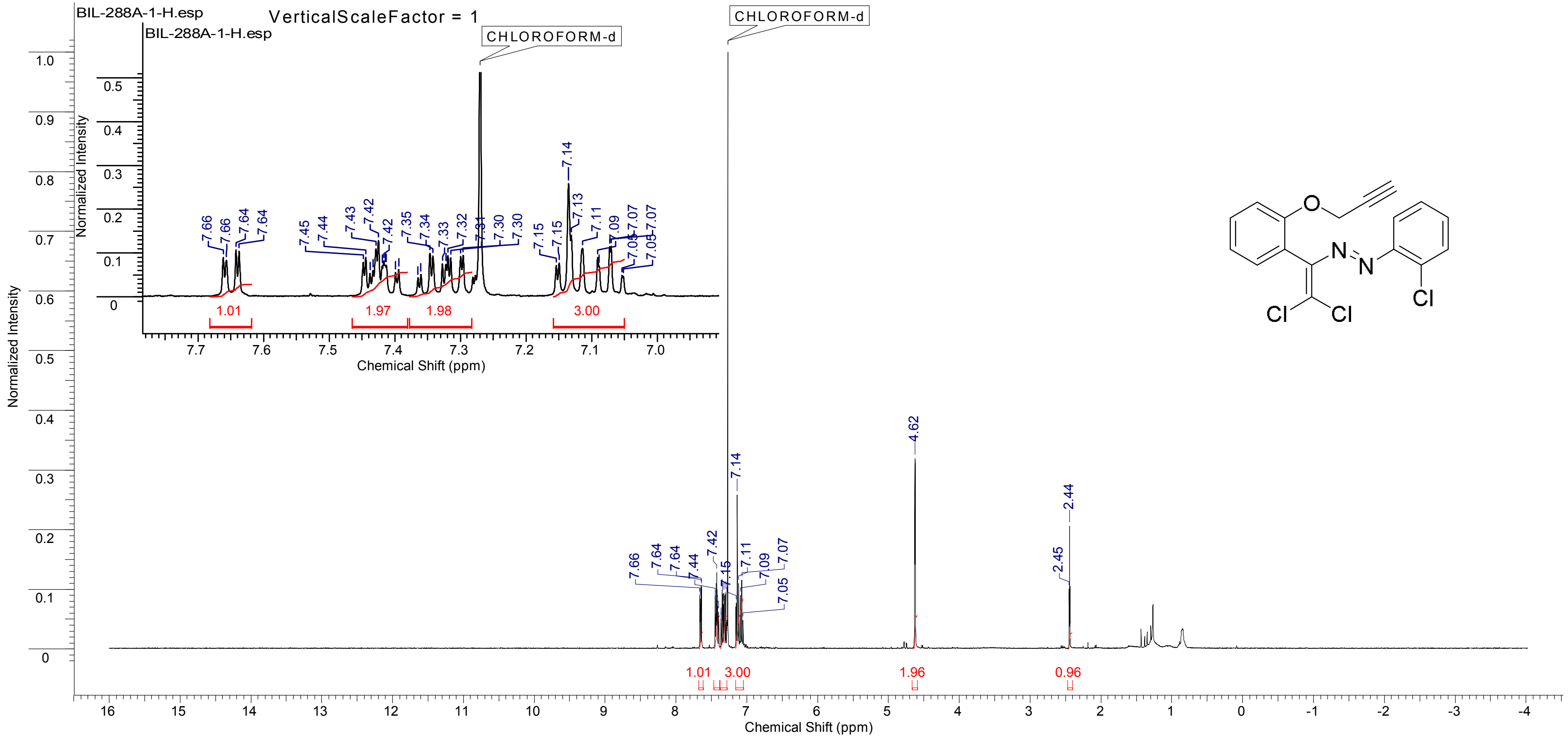

^{1}H NMR spectrum of **2j** (400.1 MHz, $CDCl_3$)

14.05.2019 18:35:53

Acquisition Time (sec)	2.0000	Date	Feb 11 2019	Date Stamp	Feb 11 2019		
File Name							
Frequency (MHz)	100.58	Nucleus	13C	Number of Transients	18000	Original Points Count	43103
Points Count	65536	Pulse Sequence	s2pul	Receiver Gain	54.00	Solvent	CHLOROFORM-d
Spectrum Offset (Hz)	9822.5713	Sweep Width (Hz)	21551.72	Temperature (degree C)	24.000		

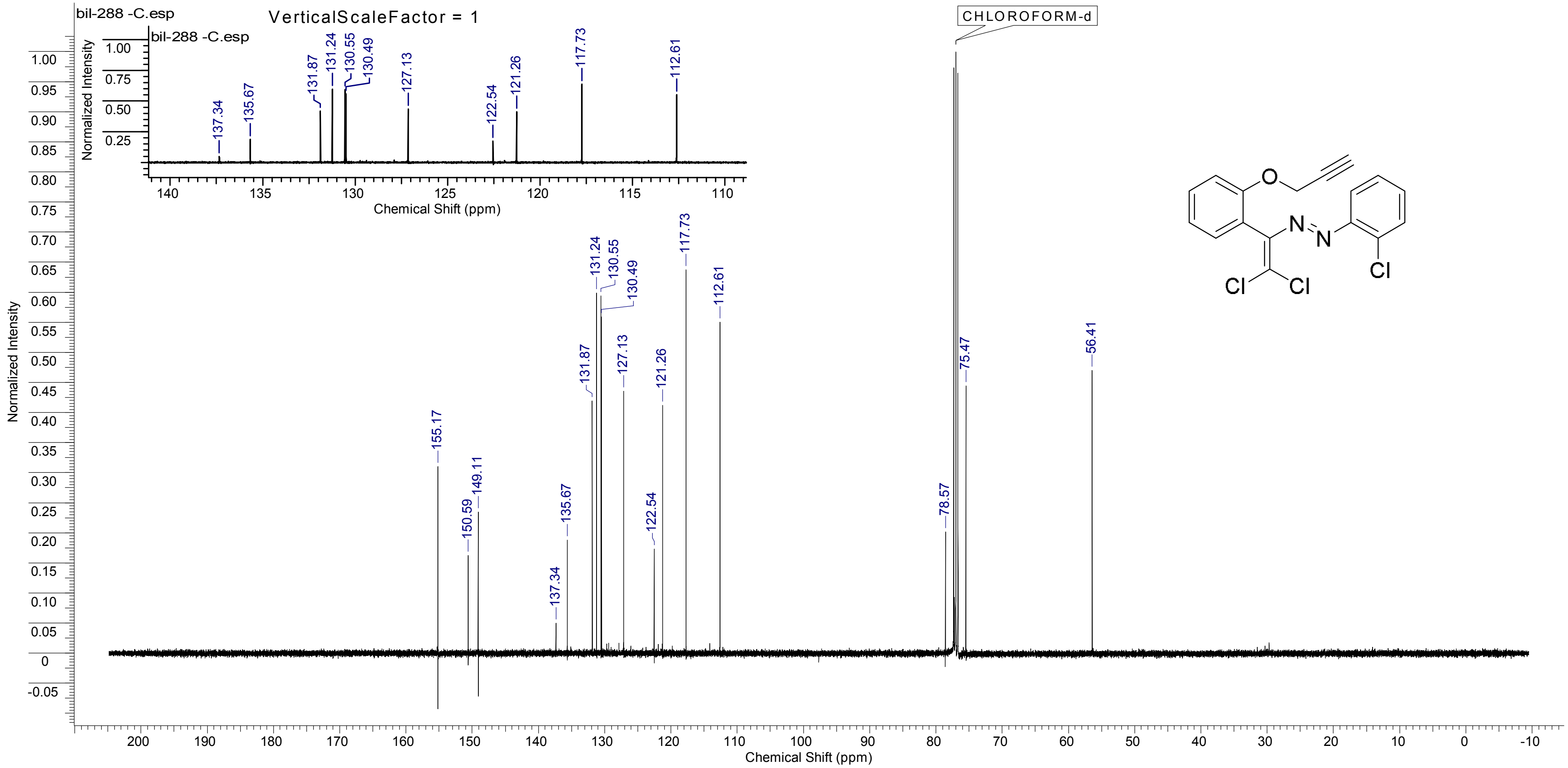

^{13}C NMR spectrum of **2j** (100.6 MHz, $CDCl_3$)

14.05.2019 18:28:59

Acquisition Time (sec)	2.5559	***Comment***	5 mm BBO BB-1H/D Z3918/0123			***Date***	31 Oct 2018 14:36:48
Date Stamp	31 Oct 2018 14:36:48						
File Name						***Frequency (MHz)***	400.13
Nucleus	1H	***Number of Transients***	5	***Origin***	spect	***Original Points Count***	16384
Owner	root	***Points Count***	65536	***Pulse Sequence***	zg30	***Receiver Gain***	362.00
SW(cyclical) (Hz)	6410.26	***Solvent***	CHLOROFORM-d			***Spectrum Offset (Hz)***	2595.7000
Sweep Width (Hz)	6410.16	***Temperature (degree C)***	27.000				

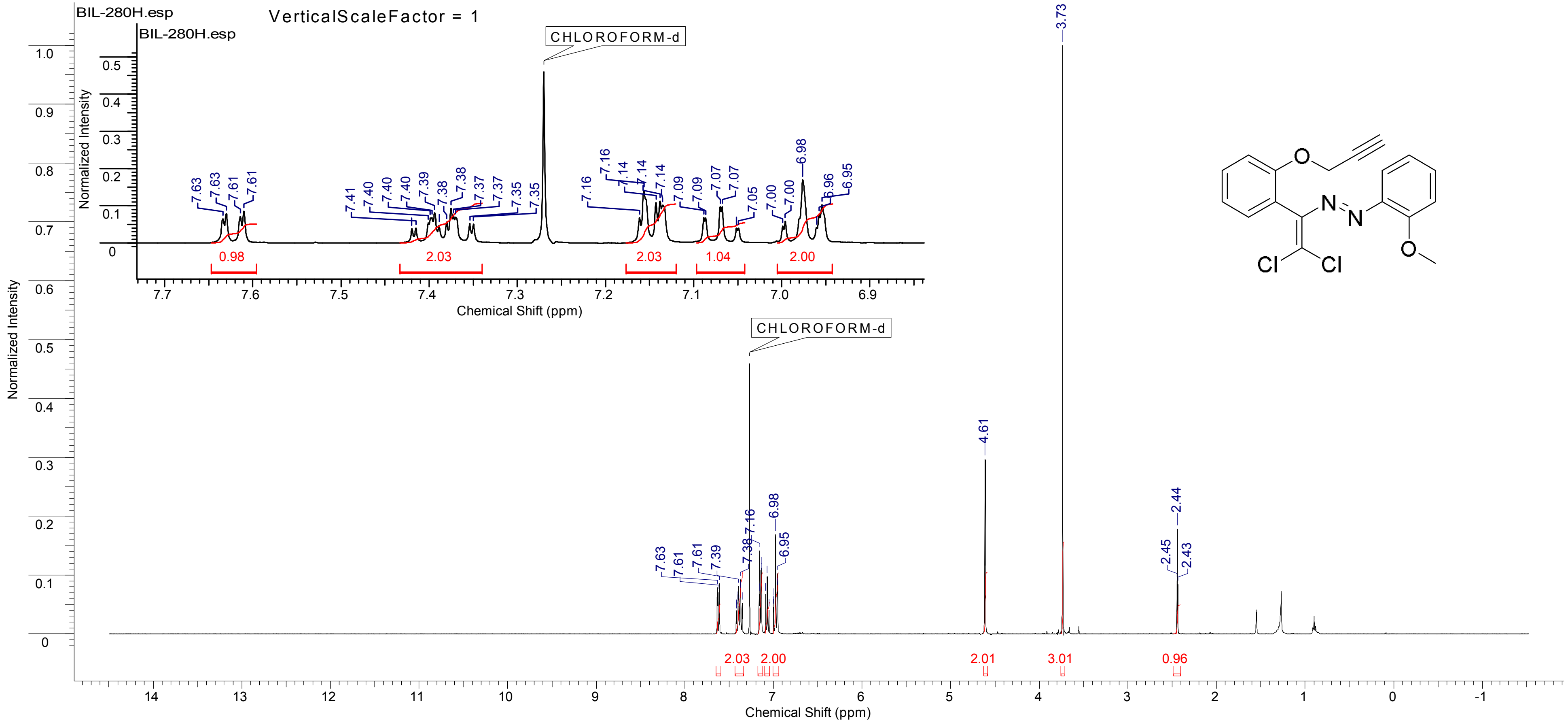

^{1}H NMR spectrum of **2k** (400.1 MHz, $CDCl_3$)

Acquisition Time (sec)	0.4999	***Comment***	5 mm BBO BB-1H/D Z3918/0123			***Date***	14 Nov 2018 14:17:36
Date Stamp	14 Nov 2018 14:17:36			***File Name***			
Frequency (MHz)	100.61	***Nucleus***	13C	***Number of Transients***	129	***Origin***	spect
Original Points Count	12076	***Owner***	root	***Points Count***	65536	***Pulse Sequence***	zgpg30
Receiver Gain	8192.00	***SW(cyclical) (Hz)***	24154.59	***Solvent***	CHLOROFORM-d		
Spectrum Offset (Hz)	11056.0225	***Sweep Width (Hz)***	24154.22	***Temperature (degree C)***	27.000		

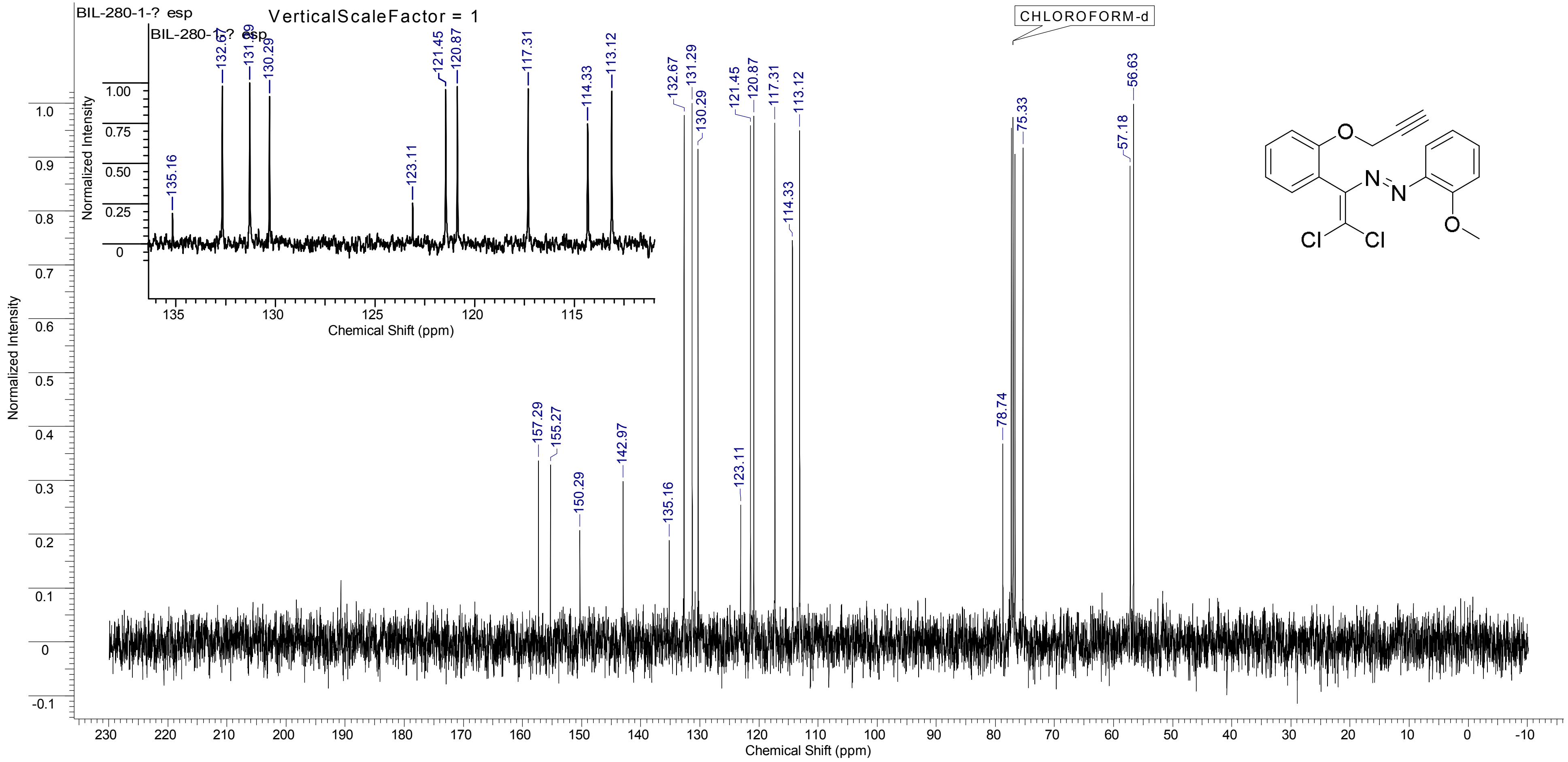

^{13}C NMR spectrum of **2k** (100.6 MHz, $CDCl_3$)

14.05.2019 19:31:46

**Acquisition Time (sec)**	4.0894	**Comment**	5 mm BBO BB-1H/D Z3918/0123			**Date**	07 Dec 2018 12:26:40		
**Date Stamp**	07 Dec 2018 12:26:40			**File Name**				**Frequency (MHz)**	400.13
**Nucleus**	1H	**Number of Transients**	4	**Origin**	spect	**Original Points Count**	32768	**Owner**	root
**Points Count**	131072	**Pulse Sequence**	zg30	**Receiver Gain**	724.10	**SW(cyclical) (Hz)**	8012.82	**Solvent**	CHLOROFORM-d
**Spectrum Offset (Hz)**	2395.7031	**Sweep Width (Hz)**	8012.76	**Temperature (degree C)**	27.000				

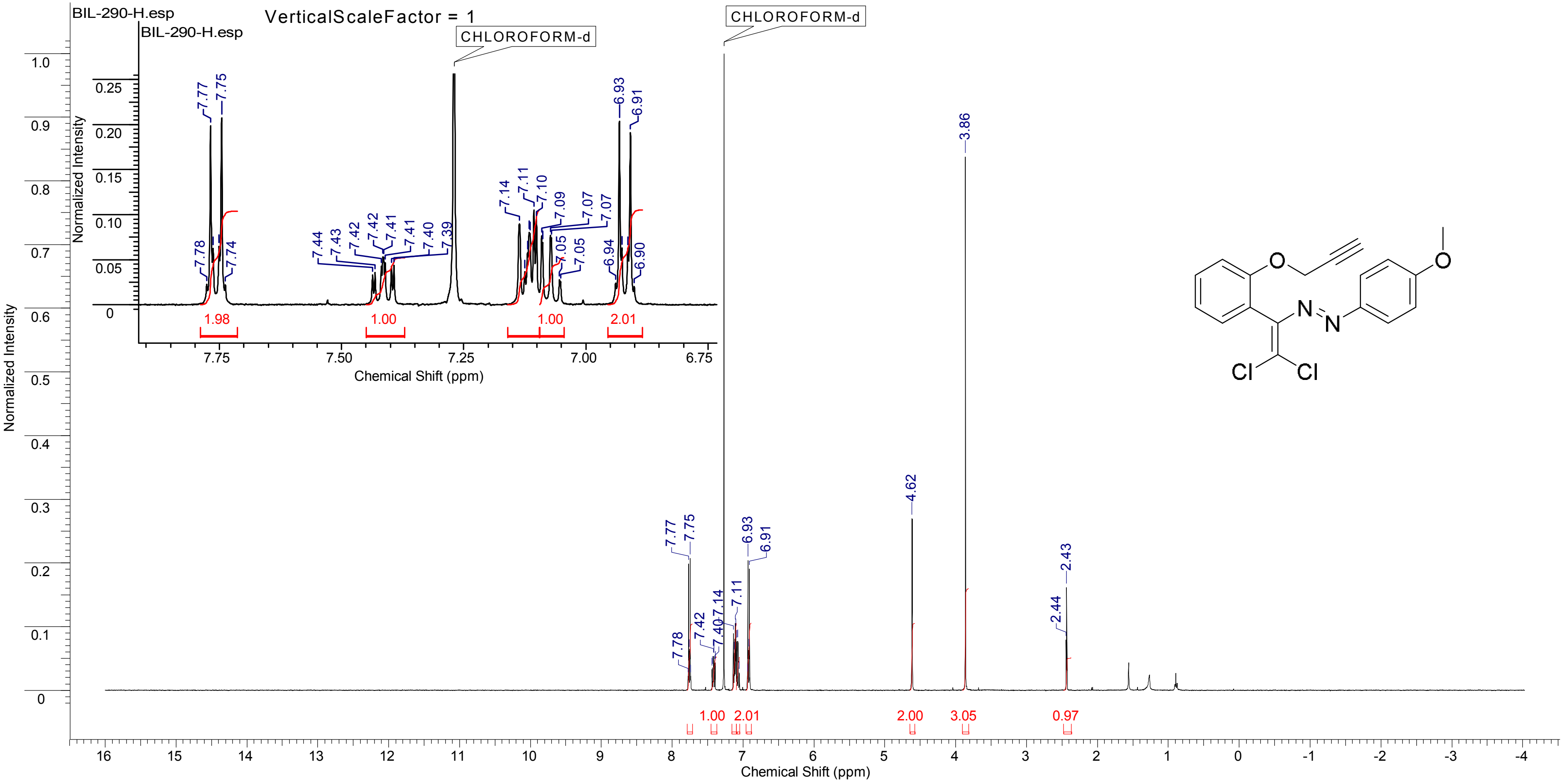

^{1}H NMR spectrum of **2l** (400.1 MHz, $CDCl_3$)

14.05.2019 19:33:00

**Acquisition Time (sec)**	0.4999	**Comment**	5 mm BBO BB-1H/D Z3918/0123			**Date**	20 Nov 2018 11:37:36		
**Date Stamp**	20 Nov 2018 11:37:36			**File Name**				**Frequency (MHz)**	100.61
**Nucleus**	13C	**Number of Transients**	195	**Origin**	spect	**Original Points Count**	12076	**Owner**	root
**Points Count**	65536	**Pulse Sequence**	zgpg30	**Receiver Gain**	8192.00	**SW(cyclical) (Hz)**	24154.59		
**Solvent**	CHLOROFORM-d			**Spectrum Offset (Hz)**	11062.2871	**Sweep Width (Hz)**	24154.22	**Temperature (degree C)**	27.000

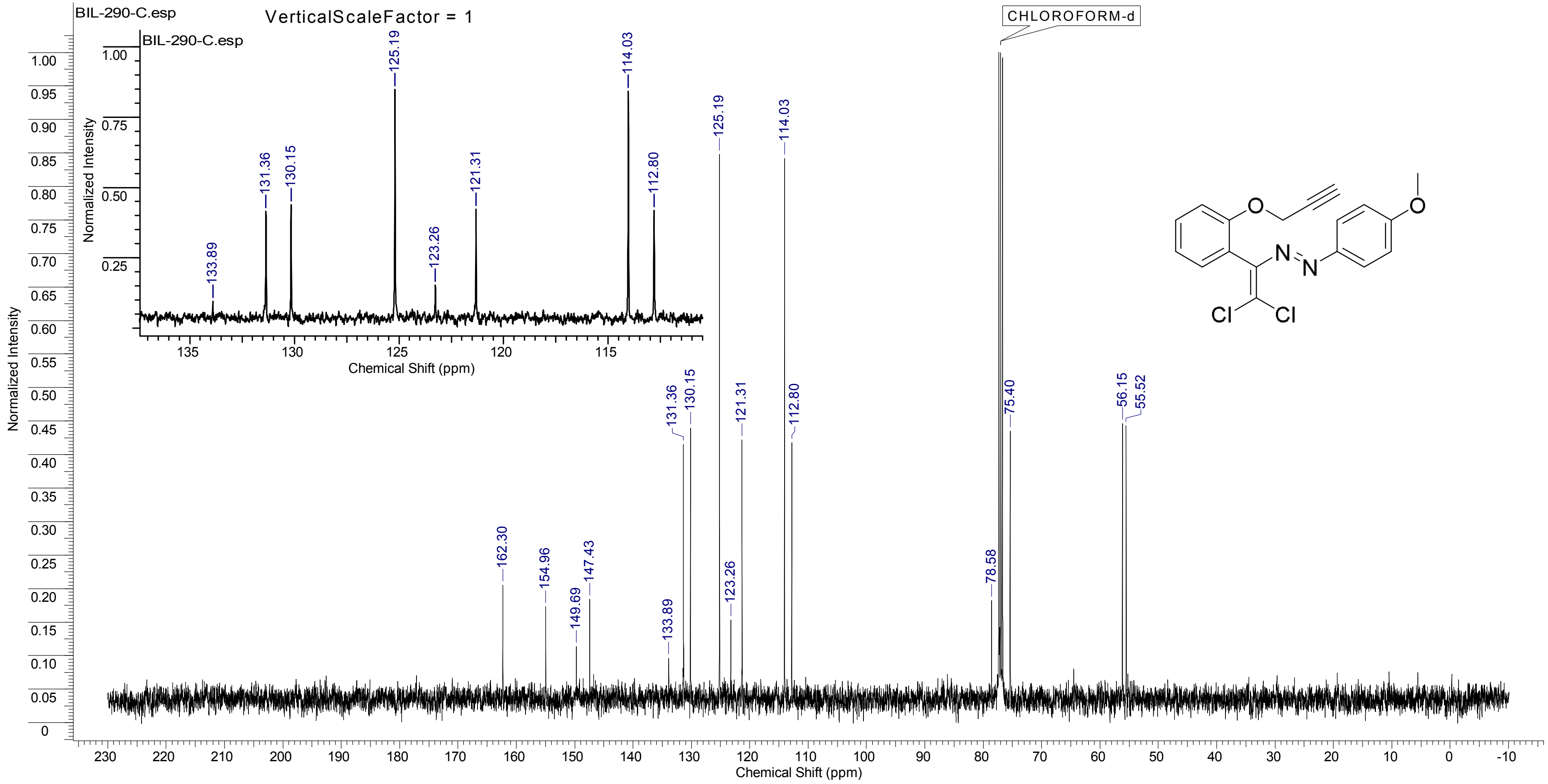

^{13}C NMR spectrum of **2l** (100.6 MHz, $CDCl_3$)

15.05.2019 12:13:45

**Acquisition Time (sec)**	4.0894	**Comment**	5 mm BBO BB-1H/D Z3918/0123			**Date**	05 Feb 2019 11:33:20		
**Date Stamp**	05 Feb 2019 11:33:20			**File Name**				**Frequency (MHz)**	400.13
**Nucleus**	1H	**Number of Transients**	5	**Origin**	spect	**Original Points Count**	32768	**Owner**	root
**Points Count**	131072	**Pulse Sequence**	zg30	**Receiver Gain**	57.00	**SW(cyclical) (Hz)**	8012.82	**Solvent**	CHLOROFORM-d
**Spectrum Offset (Hz)**	2395.8254	**Sweep Width (Hz)**	8012.76	**Temperature (degree C)**	27.000				

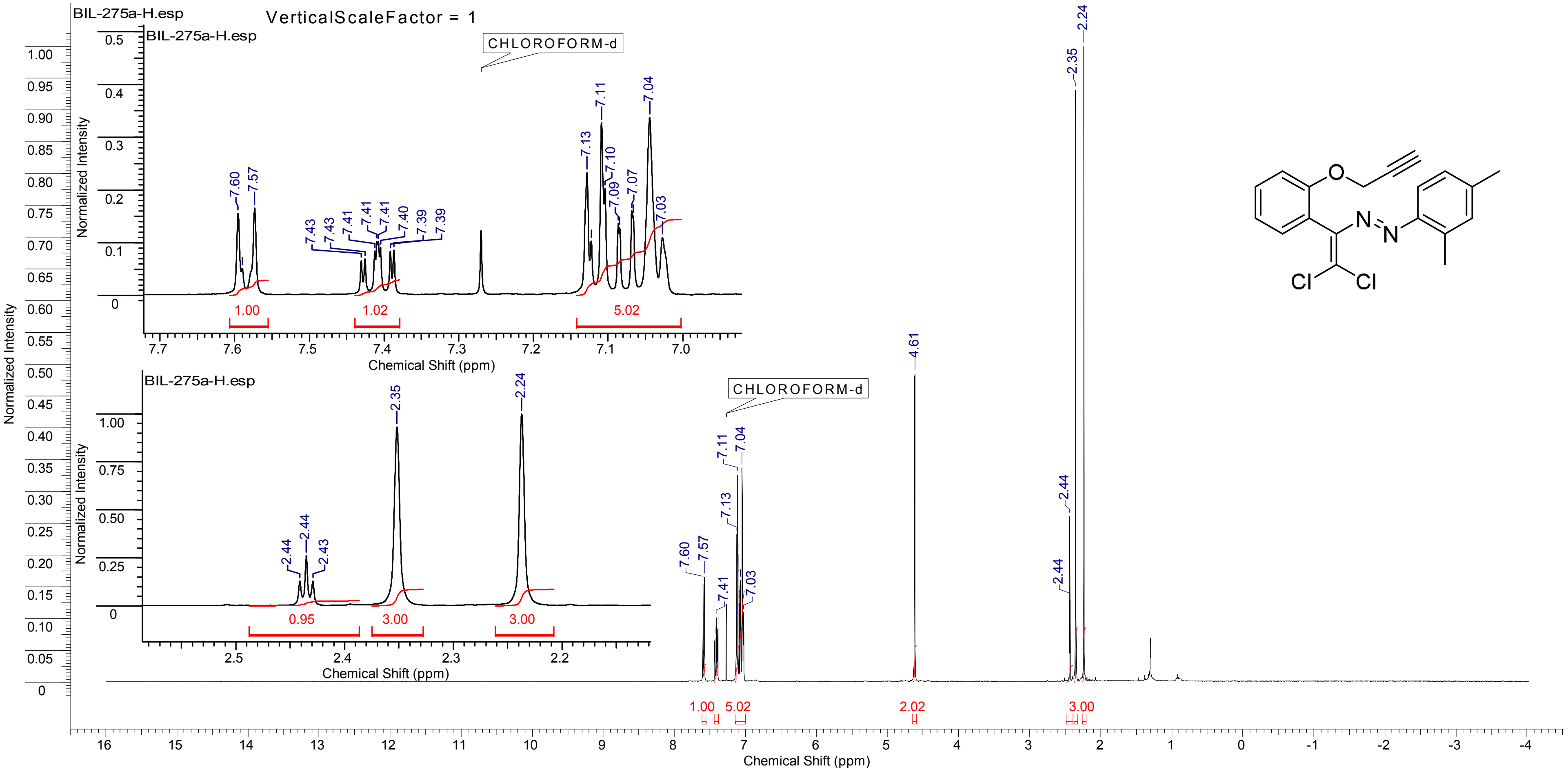

^{1}H NMR spectrum of **2m** (400.1 MHz, $CDCl_3$)

Acquisition Time (sec)	0.6783	***Comment***	5 mm BBO BB-1H/D Z3918/0123			***Date***	05 Feb 2019 11:35:28
Date Stamp	05 Feb 2019 11:35:28						
File Name						***Frequency (MHz)***	100.61
Nucleus	13C	***Number of Transients***	73	***Origin***	spect	***Original Points Count***	16384
Owner	root	***Points Count***	131072	***Pulse Sequence***	zgpg30	***Receiver Gain***	13004.00
SW(cyclical) (Hz)	24154.59	***Solvent***	CHLOROFORM-d	***Spectrum Offset (Hz)***	11060.7207	***Sweep Width (Hz)***	24154.41
Temperature (degree C)	27.000						

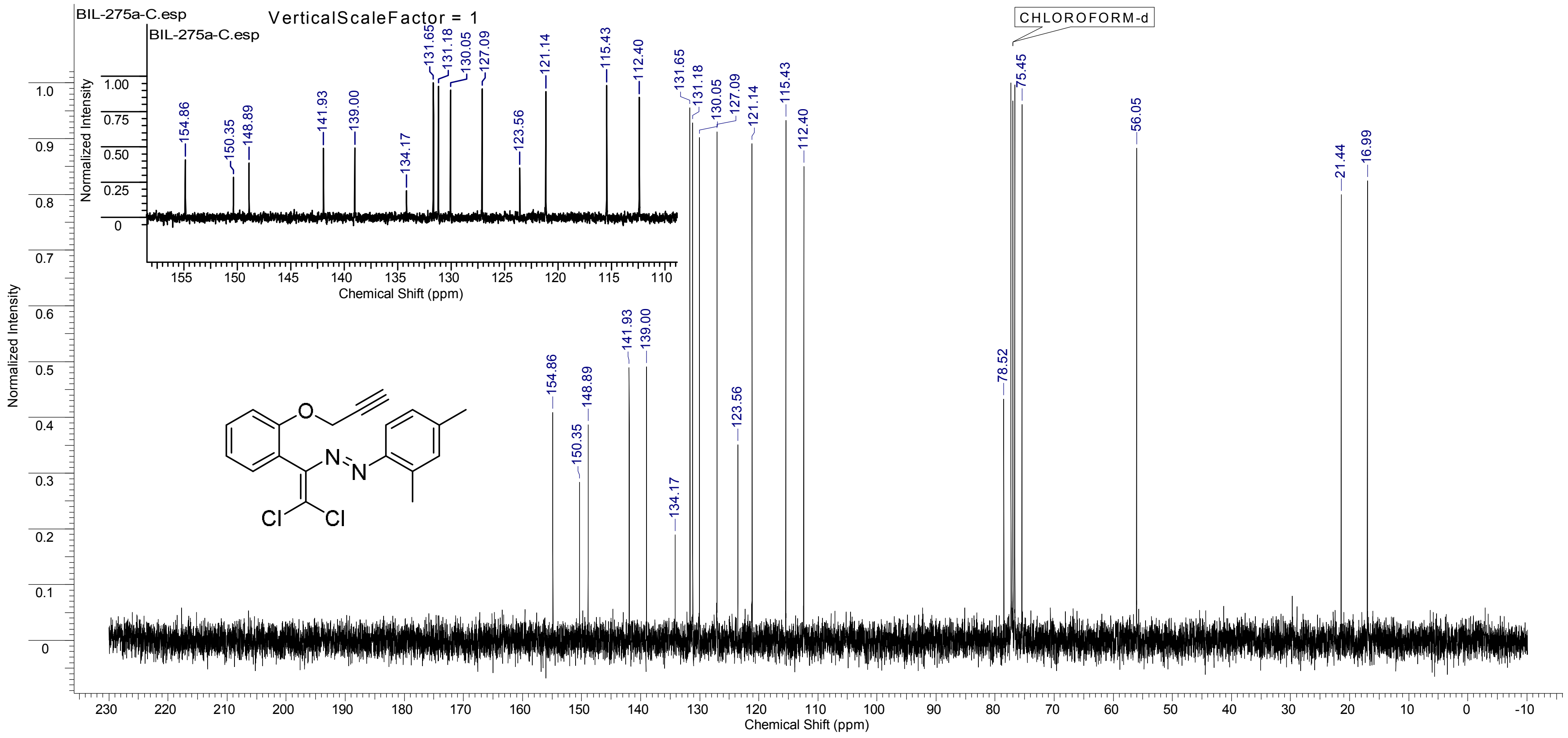

^{13}C NMR spectrum of **2m** (100.6 MHz, $CDCl_3$)

14.05.2019 20:17:46

Acquisition Time (sec)	2.5559	***Comment***	5 mm BBO BB-1H/D Z3918/0123			***Date***	21 Nov 2018 14:30:24		
Date Stamp	21 Nov 2018 14:30:24			***File Name***				***Frequency (MHz)***	400.13
Nucleus	1H	***Number of Transients***	5	***Origin***	spect	***Original Points Count***	16384	***Owner***	root
Points Count	65536	***Pulse Sequence***	zg30	***Receiver Gain***	64.00	***SW(cyclical) (Hz)***	6410.26	***Solvent***	CHLOROFORM-d
Spectrum Offset (Hz)	2595.9934	***Sweep Width (Hz)***	6410.16	***Temperature (degree C)***	27.000				

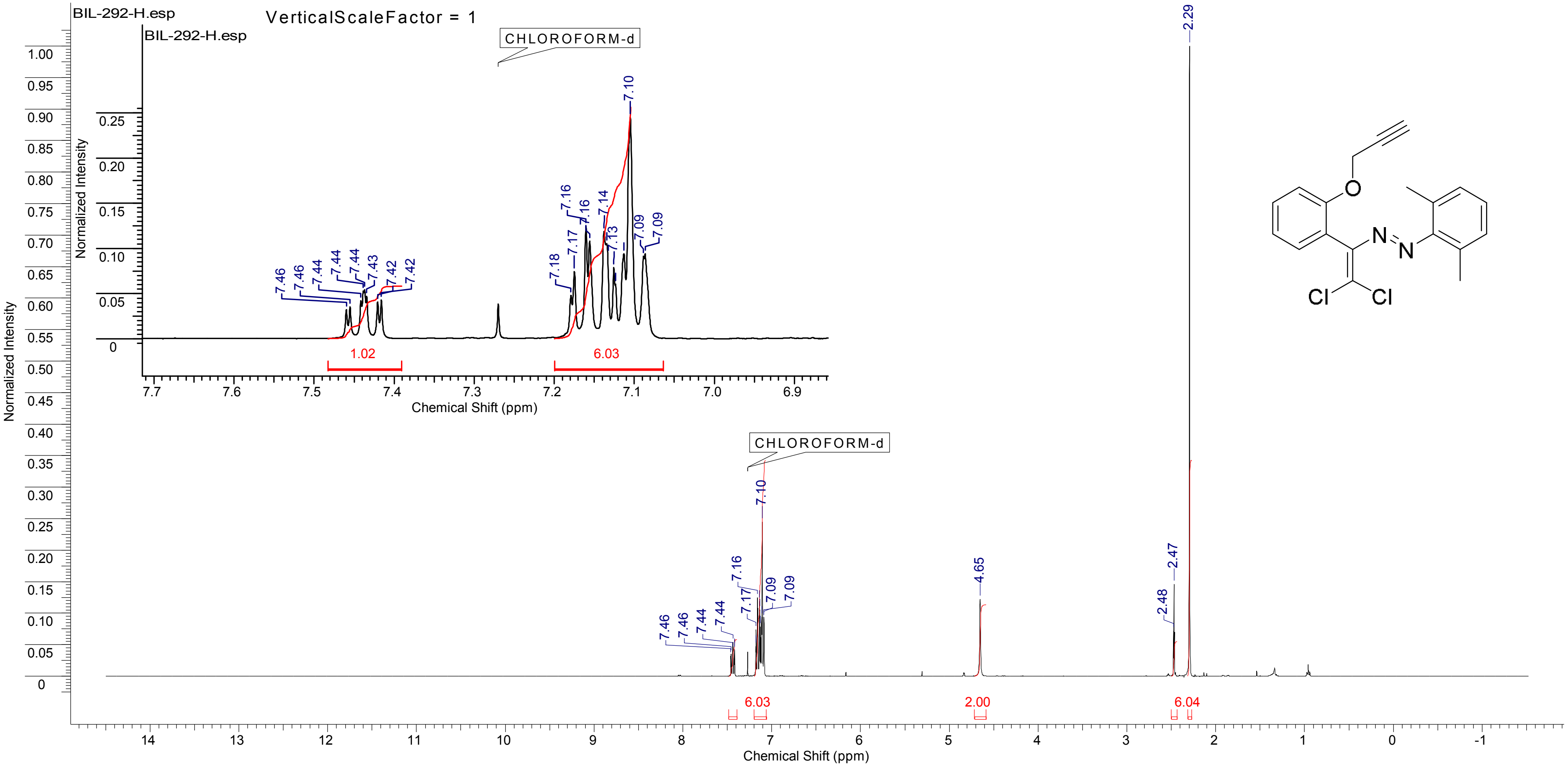

^{1}H NMR spectrum of **2n** (400.1 MHz, $CDCl_3$)

14.05.2019 20:18:43

Acquisition Time (sec)	0.4999	***Comment***	5 mm BBO BB-1H/D Z3918/0123			***Date***	22 Nov 2018 08:10:40		
Date Stamp	22 Nov 2018 08:10:40			***File Name***				***Frequency (MHz)***	100.61
Nucleus	13C	***Number of Transients***	154	***Origin***	spect	***Original Points Count***	12076	***Owner***	root
Points Count	65536	***Pulse Sequence***	zgpg30	***Receiver Gain***	8192.00	***SW(cyclical) (Hz)***	24154.59		
Solvent	CHLOROFORM-d			***Spectrum Offset (Hz)***	11053.8105	***Sweep Width (Hz)***	24154.22	***Temperature (degree C)***	27.000

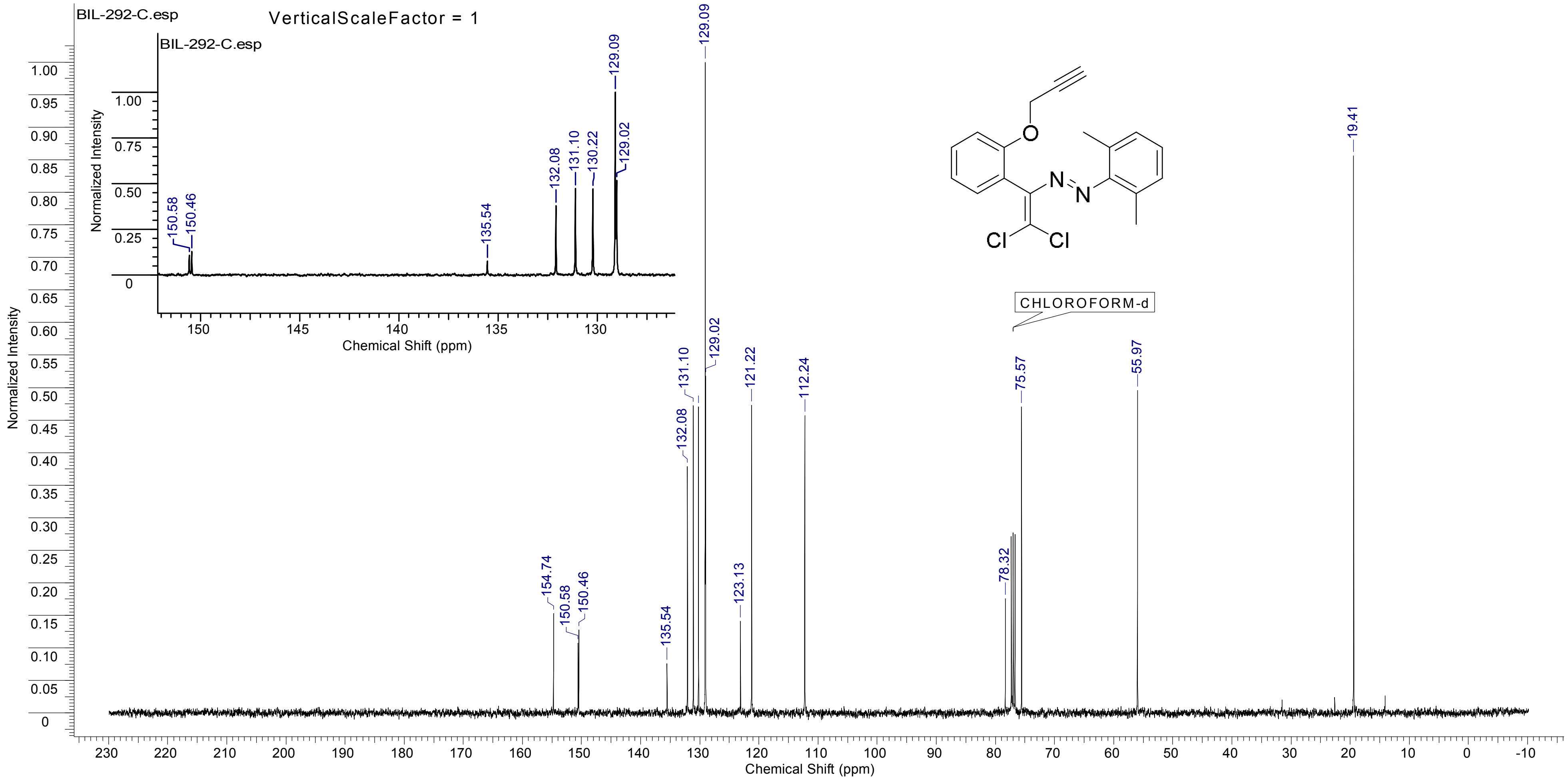

^{13}C NMR spectrum of **2n** (100.6 MHz, $CDCl_3$)

14.05.2019 20:43:48

**Acquisition Time (sec)**	4.0894	**Comment**	5 mm BBO BB-1H/D Z3918/0123			**Date**	05 Mar 2019 11:33:20
**Date Stamp**	05 Mar 2019 11:33:20						
**File Name**						**Frequency (MHz)**	400.13
**Nucleus**	1H	**Number of Transients**	4	**Origin**	spect	**Original Points Count**	32768
**Owner**	root	**Points Count**	131072	**Pulse Sequence**	zg30	**Receiver Gain**	90.50
**SW(cyclical) (Hz)**	8012.82	**Solvent**	CHLOROFORM-d			**Spectrum Offset (Hz)**	2395.8254
**Sweep Width (Hz)**	8012.76	**Temperature (degree C)**	27.000				

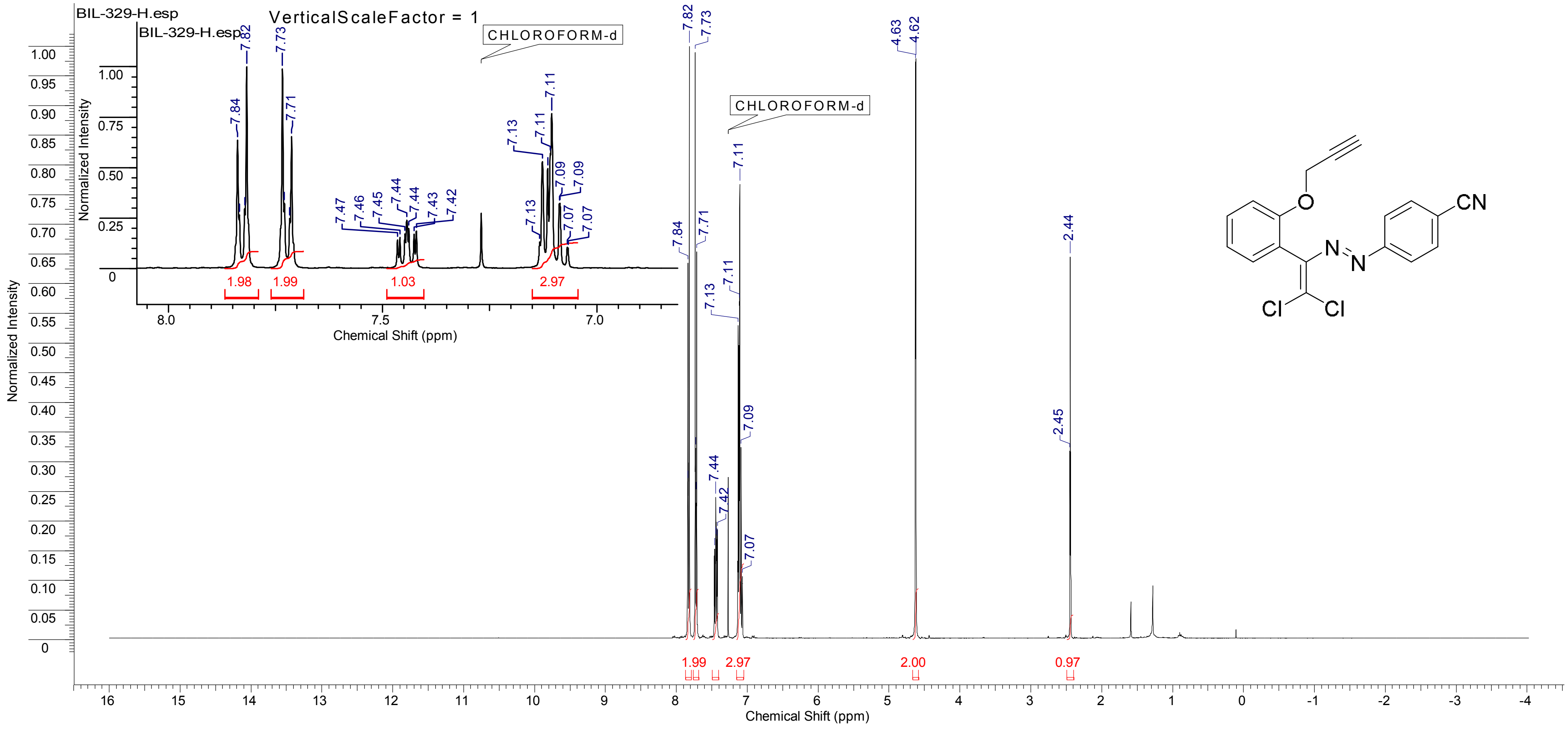

^{1}H NMR spectrum of **2o** (400.1 MHz, $CDCl_3$)

14.05.2019 20:45:16

Acquisition Time (sec)	0.6783	***Comment***	5 mm BBO BB-1H/D Z3918/0123			***Date***	05 Mar 2019 11:35:28
Date Stamp	05 Mar 2019 11:35:28						
File Name						***Frequency (MHz)***	100.61
Nucleus	13C	***Number of Transients***	65	***Origin***	spect	***Original Points Count***	16384
Owner	root	***Points Count***	131072	***Pulse Sequence***	zgpg30	***Receiver Gain***	16384.00
SW(cyclical) (Hz)	24154.59	***Solvent***	CHLOROFORM-d	***Spectrum Offset (Hz)***	11057.0352	***Sweep Width (Hz)***	24154.41
Temperature (degree C)	27.000						

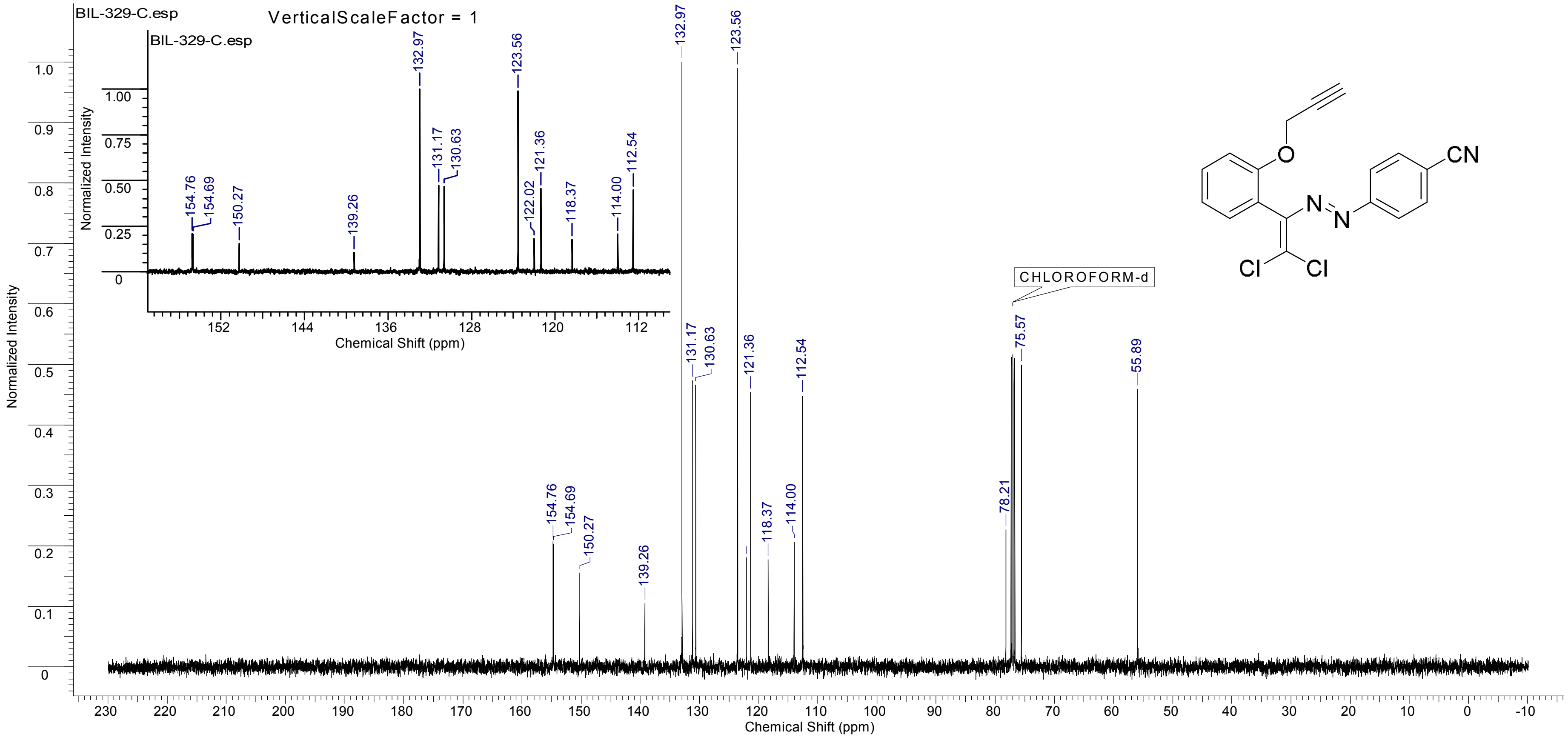

^{13}C NMR spectrum of **2o** (100.6 MHz, $CDCl_3$)

18.04.2019 20:22:01

Acquisition Time (sec)	2.5559	***Comment***	5 mm BBO BB-1H/D Z3918/0123			***Date***	18 Oct 2018 09:16:48		
Date Stamp	18 Oct 2018 09:16:48			***File Name***				***Frequency (MHz)***	400.13
Nucleus	1H	***Number of Transients***	4	***Origin***	spect	***Original Points Count***	16384	***Owner***	root
Points Count	65536	***Pulse Sequence***	zg30	***Receiver Gain***	90.50	***SW(cyclical) (Hz)***	6410.26	***Solvent***	CHLOROFORM-d
Spectrum Offset (Hz)	2595.8953	***Sweep Width (Hz)***	6410.16	***Temperature (degree C)***	27.000				

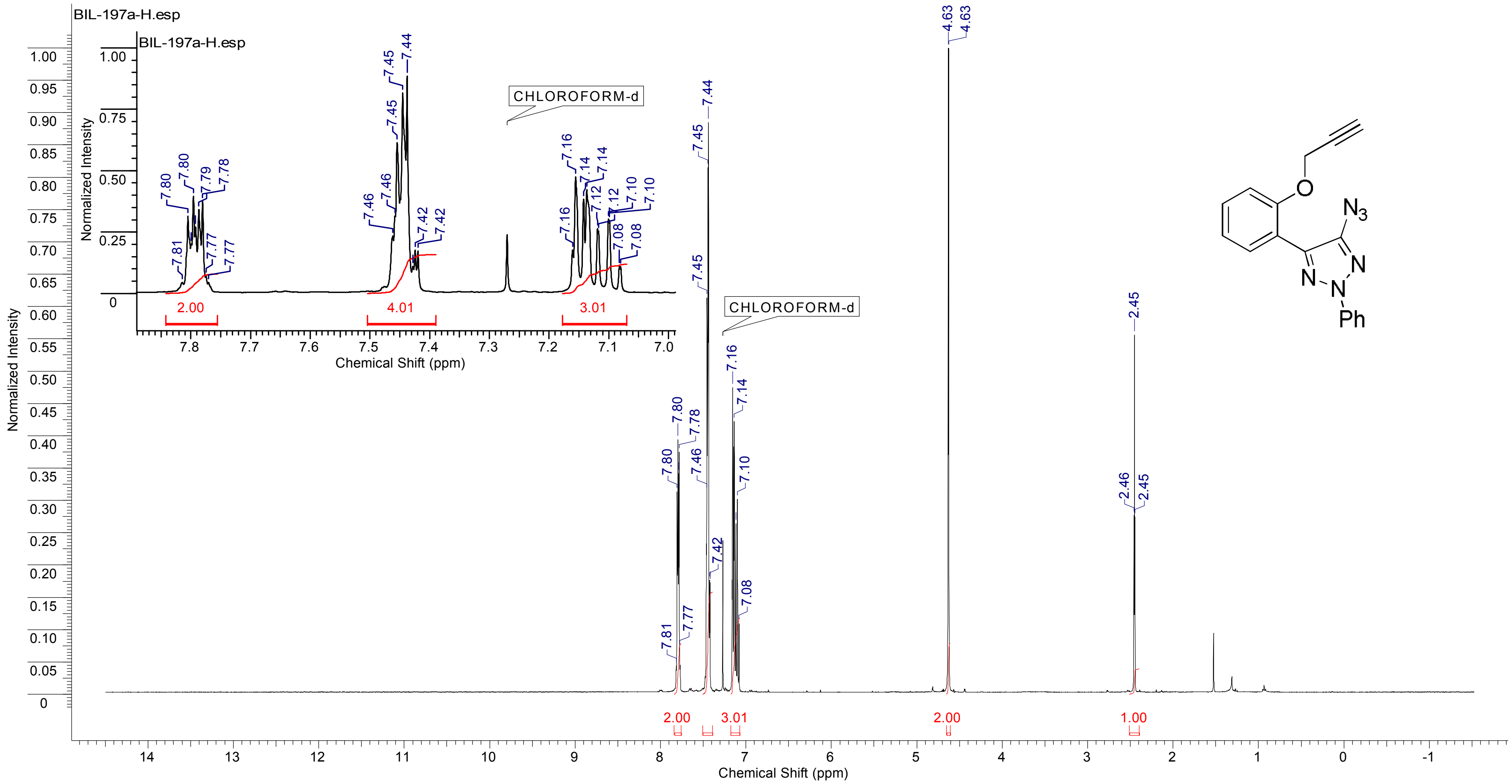

^{1}H NMR spectrum of **3a** (400.1 MHz, $CDCl_3$)

18.04.2019 20:24:13

**Acquisition Time (sec)**	0.4999	**Comment**	5 mm BBO BB-1H/D Z3918/0123			**Date**	18 Oct 2018 09:18:56		
**Date Stamp**	18 Oct 2018 09:18:56			**File Name**				**Frequency (MHz)**	100.61
**Nucleus**	13C	**Number of Transients**	102	**Origin**	spect	**Original Points Count**	12076	**Owner**	root
**Points Count**	65536	**Pulse Sequence**	zgpg30	**Receiver Gain**	10321.30	**SW(cyclical) (Hz)**	24154.59		
**Solvent**	CHLOROFORM-d			**Spectrum Offset (Hz)**	11057.4961	**Sweep Width (Hz)**	24154.22	**Temperature (degree C)**	27.000

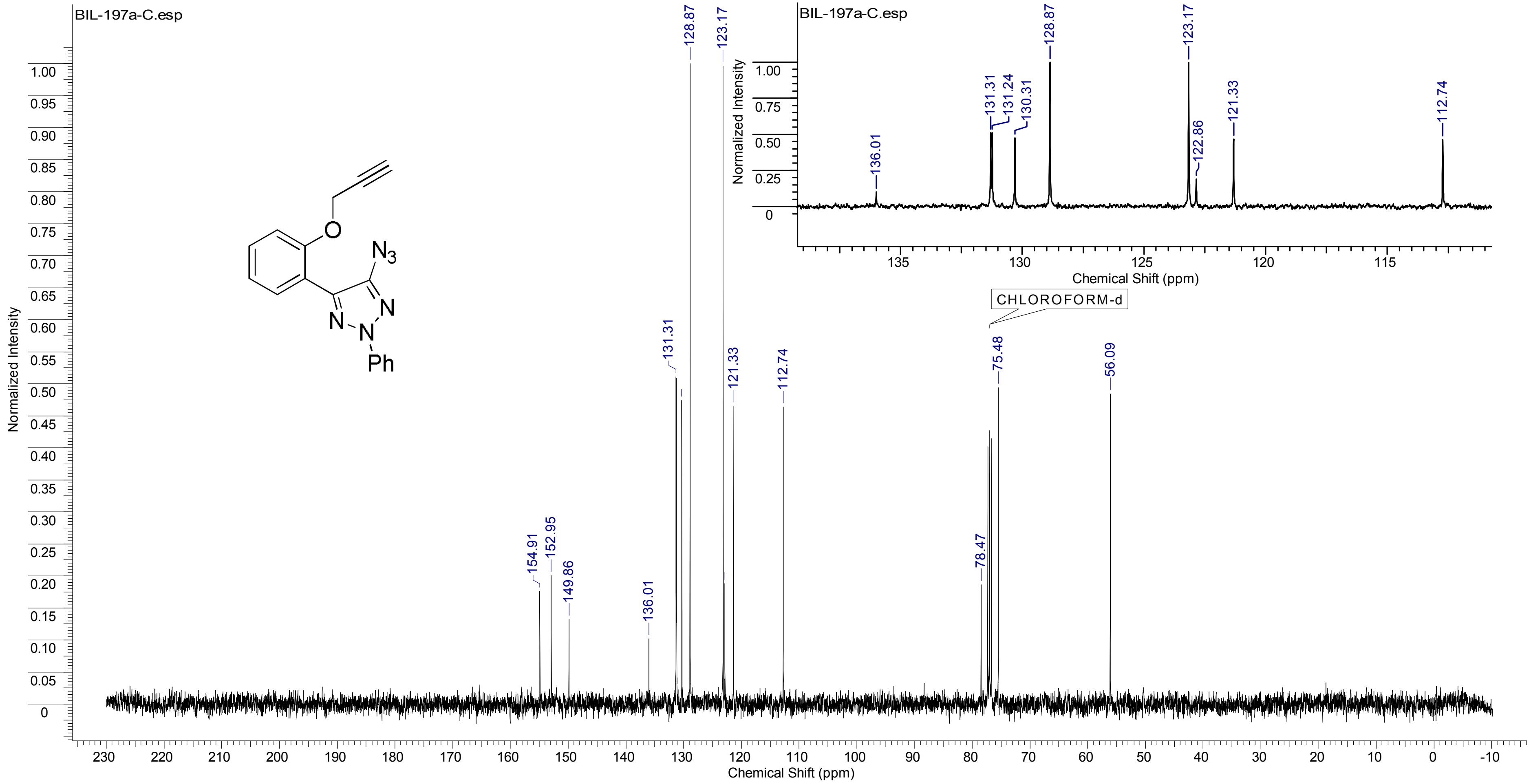

^{13}C NMR spectrum of **3a** (100.6 MHz, $CDCl_3$)

15.05.2019 10:41:18

Acquisition Time (sec)	4.0894	Comment	5 mm BBO BB-1H/D Z3918/0123	Date	07 May 2019 11:29:04
Date Stamp	07 May 2019 11:29:04				
File Name				Frequency (MHz)	400.13
Nucleus	1H	Number of Transients	4	Origin	spect	Original Points Count	32768
Owner	root	Points Count	131072	Pulse Sequence	zg30	Receiver Gain	114.00
SW(cyclical) (Hz)	8012.82	Solvent	CHLOROFORM-d	Spectrum Offset (Hz)	2396.0088
Sweep Width (Hz)	8012.76	Temperature (degree C)	27.000		

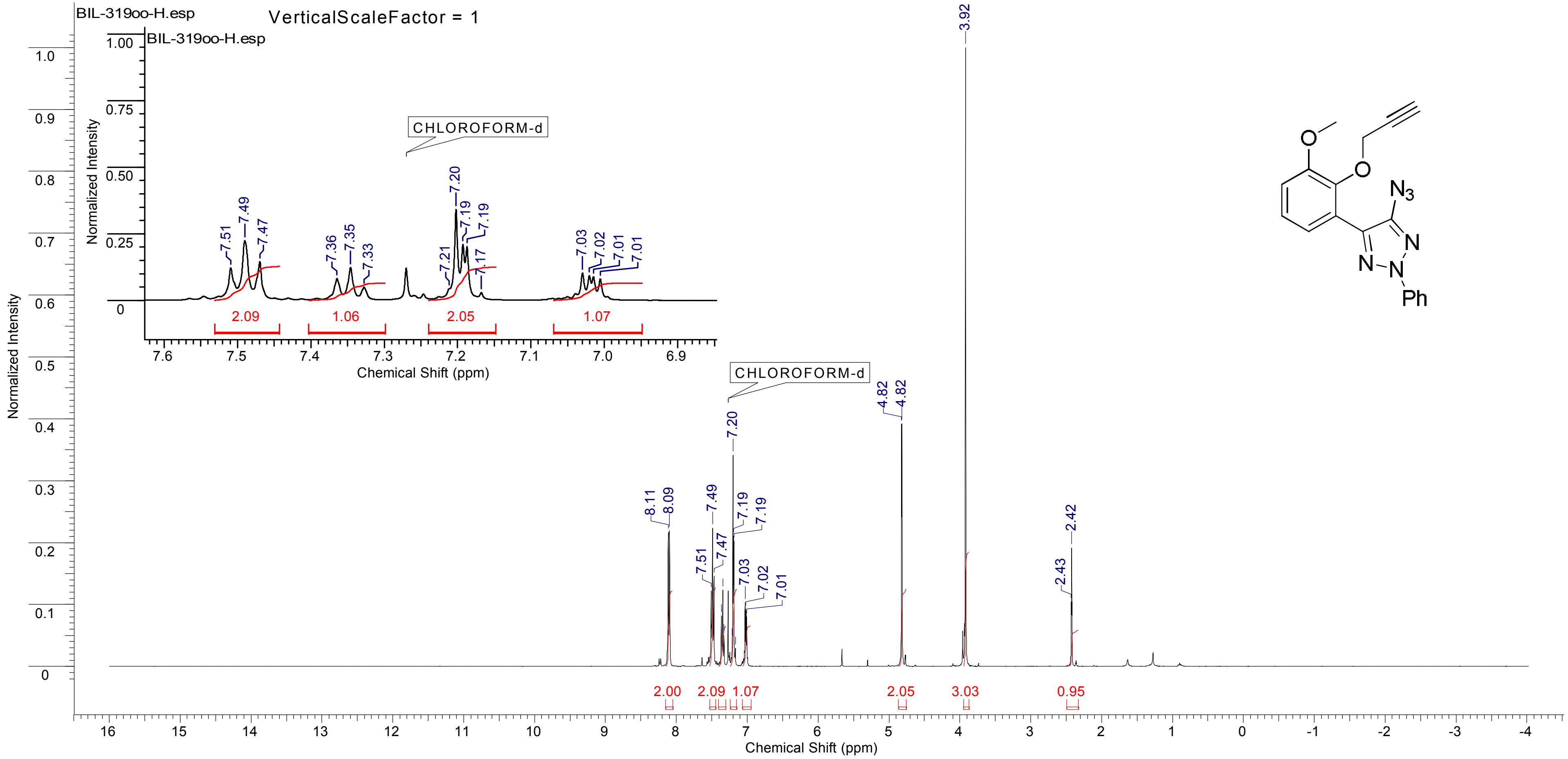

^{1}H NMR spectrum of **3b** (400.1 MHz, $CDCl_3$)

15.05.2019 10:53:28

Acquisition Time (sec)	0.6783	***Comment***	5 mm BBO BB-1H/D Z3918/0123			***Date***	07 May 2019 11:29:04
Date Stamp	07 May 2019 11:29:04						
File Name						***Frequency (MHz)***	100.61
Nucleus	13C	***Number of Transients***	88	***Origin***	spect	***Original Points Count***	16384
Owner	root	***Points Count***	131072	***Pulse Sequence***	zgpg30	***Receiver Gain***	13004.00
SW(cyclical) (Hz)	24154.59	***Solvent***	CHLOROFORM-d	***Spectrum Offset (Hz)***	11059.4307	***Sweep Width (Hz)***	24154.41
Temperature (degree C)	27.000						

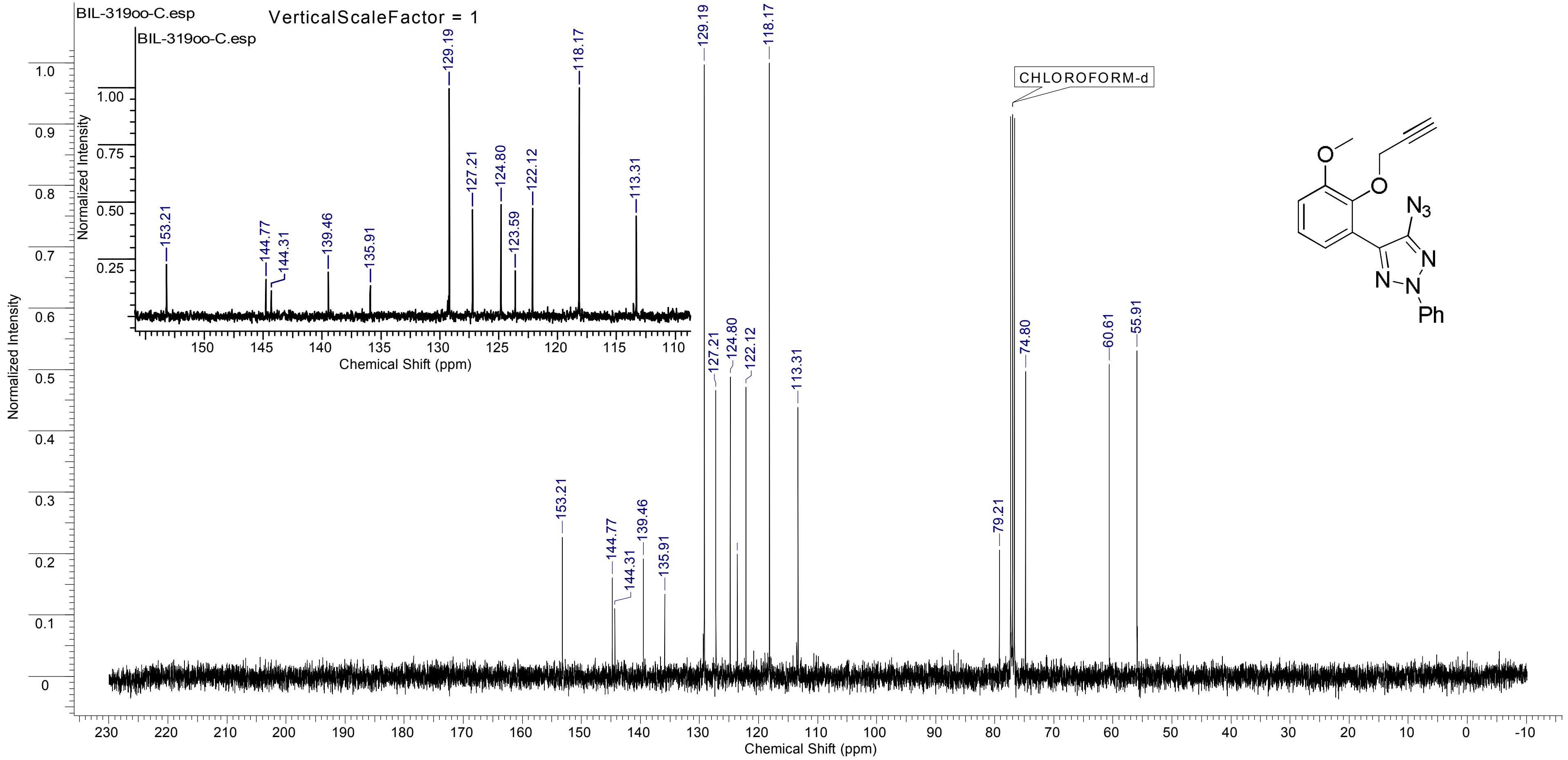

^{13}C NMR spectrum of **3b** (100.6 MHz, $CDCl_3$)

18.04.2019 22:52:08

Acquisition Time (sec)	4.0894	***Comment***	5 mm Dual 13C/1H Z3756/0200			***Date***	22 Feb 2019 12:07:28
Date Stamp	22 Feb 2019 12:07:28						
File Name							
Frequency (MHz)	400.13	***Nucleus***	1H	***Number of Transients***	4	***Origin***	spect
Original Points Count	32768	***Owner***	root	***Points Count***	131072	***Pulse Sequence***	zg30
Receiver Gain	114.00	***SW(cyclical) (Hz)***	8012.82	***Solvent***	CHLOROFORM-d	***Spectrum Offset (Hz)***	2396.3145
Sweep Width (Hz)	8012.76	***Temperature (degree C)***	27.000				

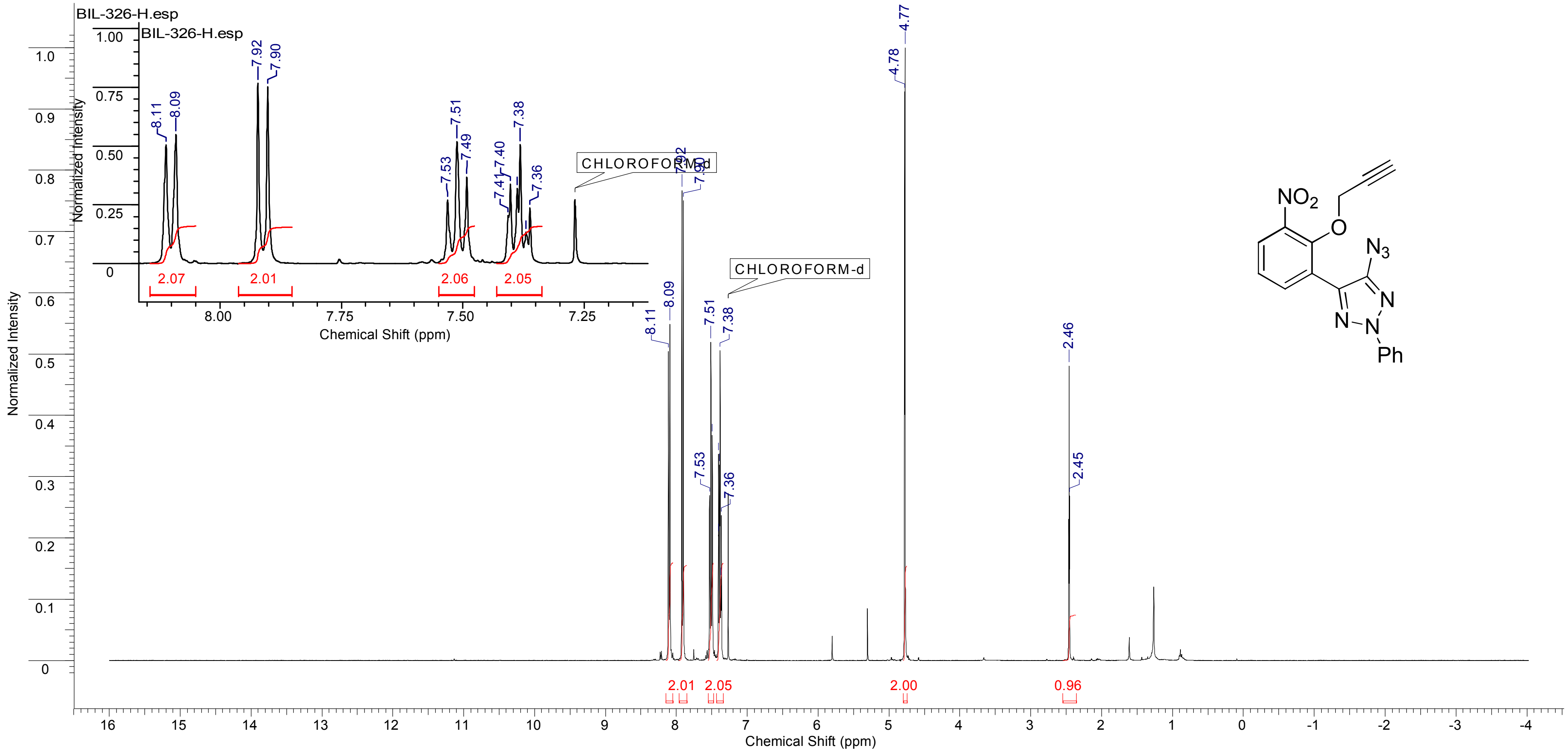

^1H NMR spectrum of **3c** (400.1 MHz, $CDCl_3$)

18.04.2019 22:53:59

**Acquisition Time (sec)**	0.6783	**Comment**	5 mm Dual 13C/1H Z3756/0200			**Date**	22 Feb 2019 12:18:08
**Date Stamp**	22 Feb 2019 12:18:08						
**File Name**							
**Frequency (MHz)**	100.61	**Nucleus**	13C	**Number of Transients**	61	**Origin**	spect
**Original Points Count**	16384	**Owner**	root	**Points Count**	131072	**Pulse Sequence**	zgpg30
**Receiver Gain**	14596.50	**SW(cyclical) (Hz)**	24154.59	**Solvent**	CHLOROFORM-d	**Spectrum Offset (Hz)**	11060.3525
**Sweep Width (Hz)**	24154.41	**Temperature (degree C)**	27.000				

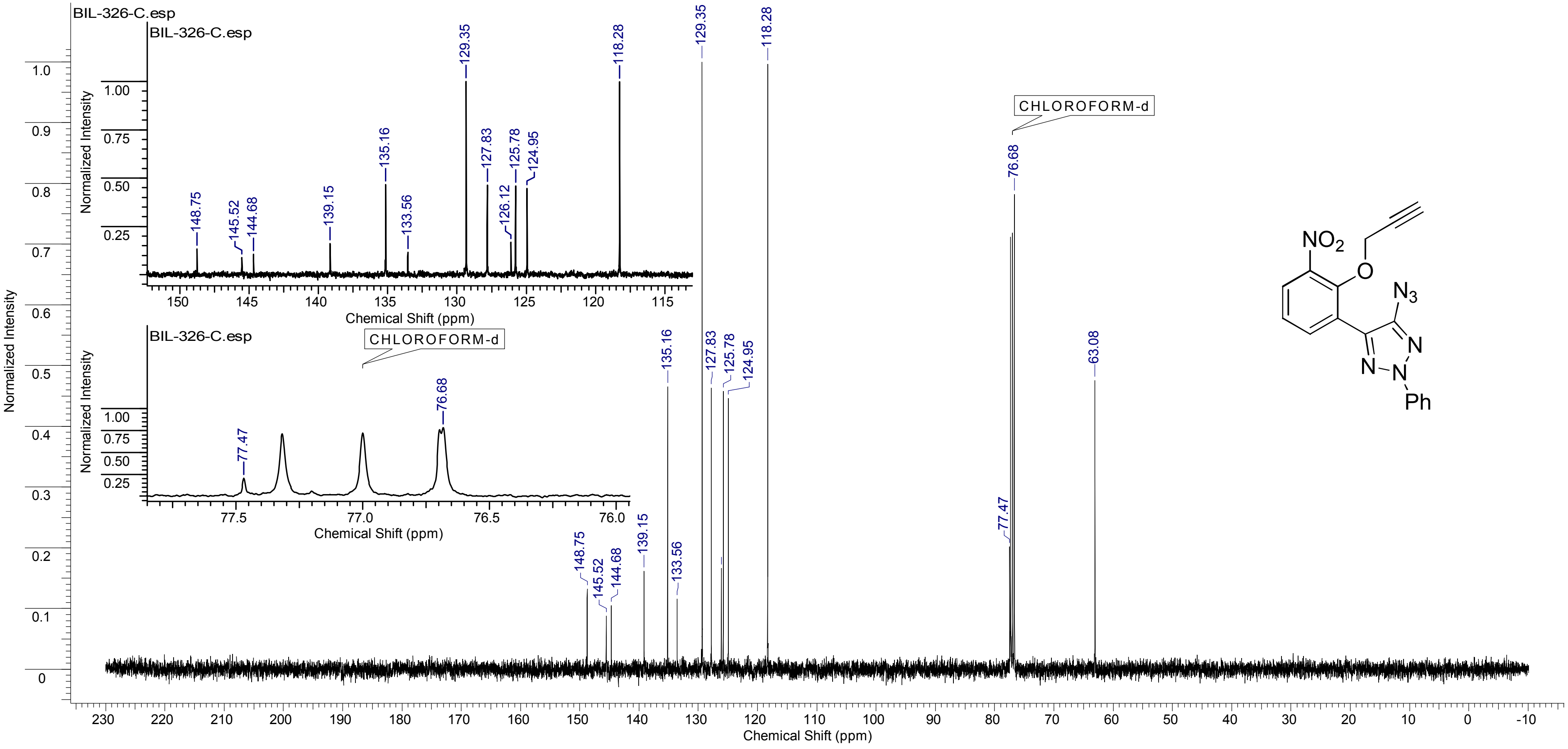

^{13}C NMR spectrum of **3c** (100.6 MHz, $CDCl_3$)

18.04.2019 22:31:10

Acquisition Time (sec)	4.0894	***Comment***	5 mm Dual 13C/1H Z3756/0200			***Date***	19 Feb 2019 12:56:32
Date Stamp	19 Feb 2019 12:56:32						
File Name						***Frequency (MHz)***	400.13
Nucleus	1H	***Number of Transients***	6	***Origin***	spect	***Original Points Count***	32768
Owner	root	***Points Count***	131072	***Pulse Sequence***	zg30	***Receiver Gain***	287.40
SW(cyclical) (Hz)	8012.82	***Solvent***	CHLOROFORM-d			***Spectrum Offset (Hz)***	2398.2097
Sweep Width (Hz)	8012.76	***Temperature (degree C)***	27.000				

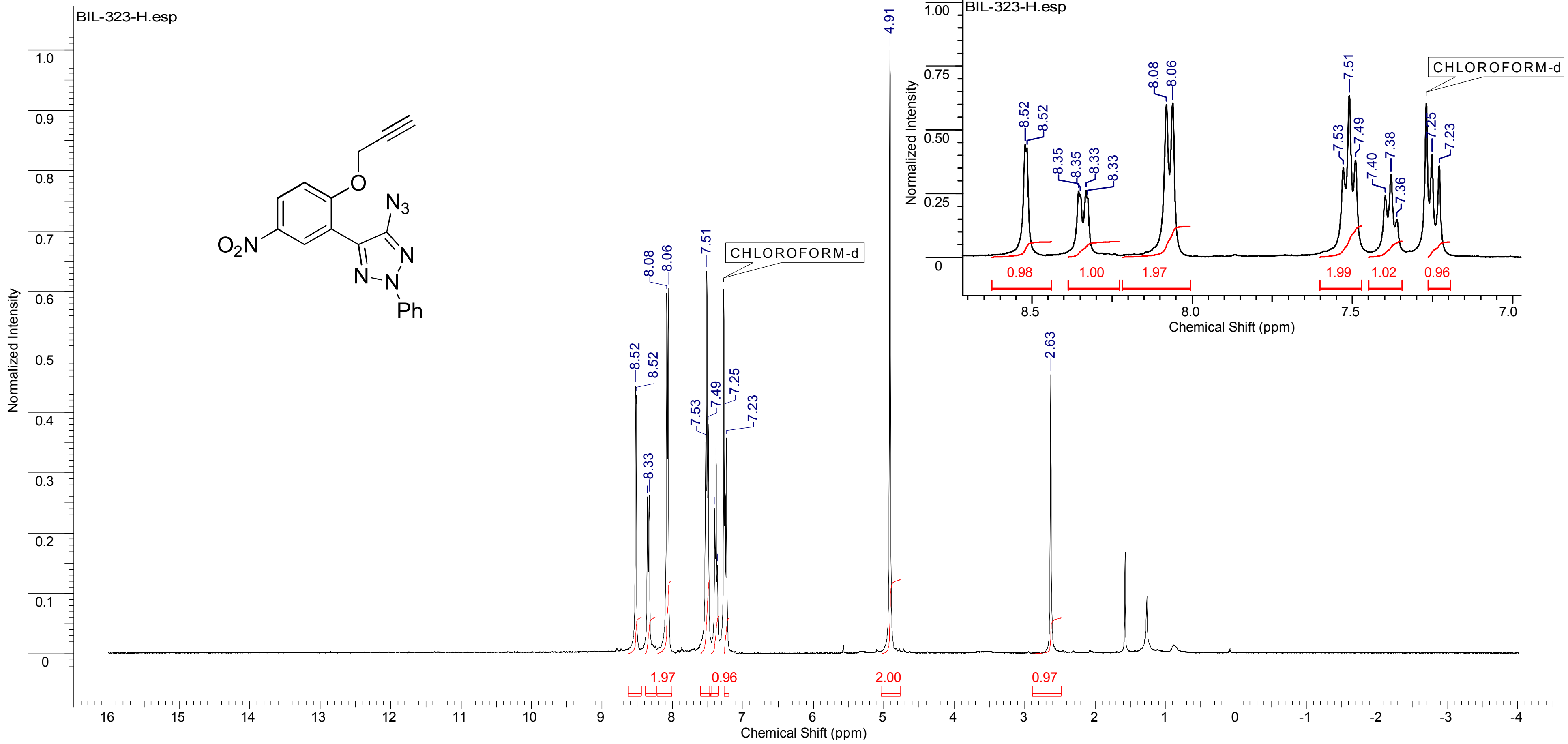

^{1}H NMR spectrum of **3d** (400.1 MHz, $CDCl_3$)

18.04.2019 22:34:09

Acquisition Time (sec)	0.6783	***Comment***	Imported from UXNMR.			***Date***	20 Feb 2019 18:00:04
File Name						***Frequency (MHz)***	100.61
Nucleus	13C	***Number of Transients***	113	***Original Points Count***	16384	***Points Count***	131072
Pulse Sequence	zgpg30	***Solvent***	DMSO-d6	***Spectrum Offset (Hz)***	11021.4902	***Sweep Width (Hz)***	24154.59
Temperature (degree C)	27.000						

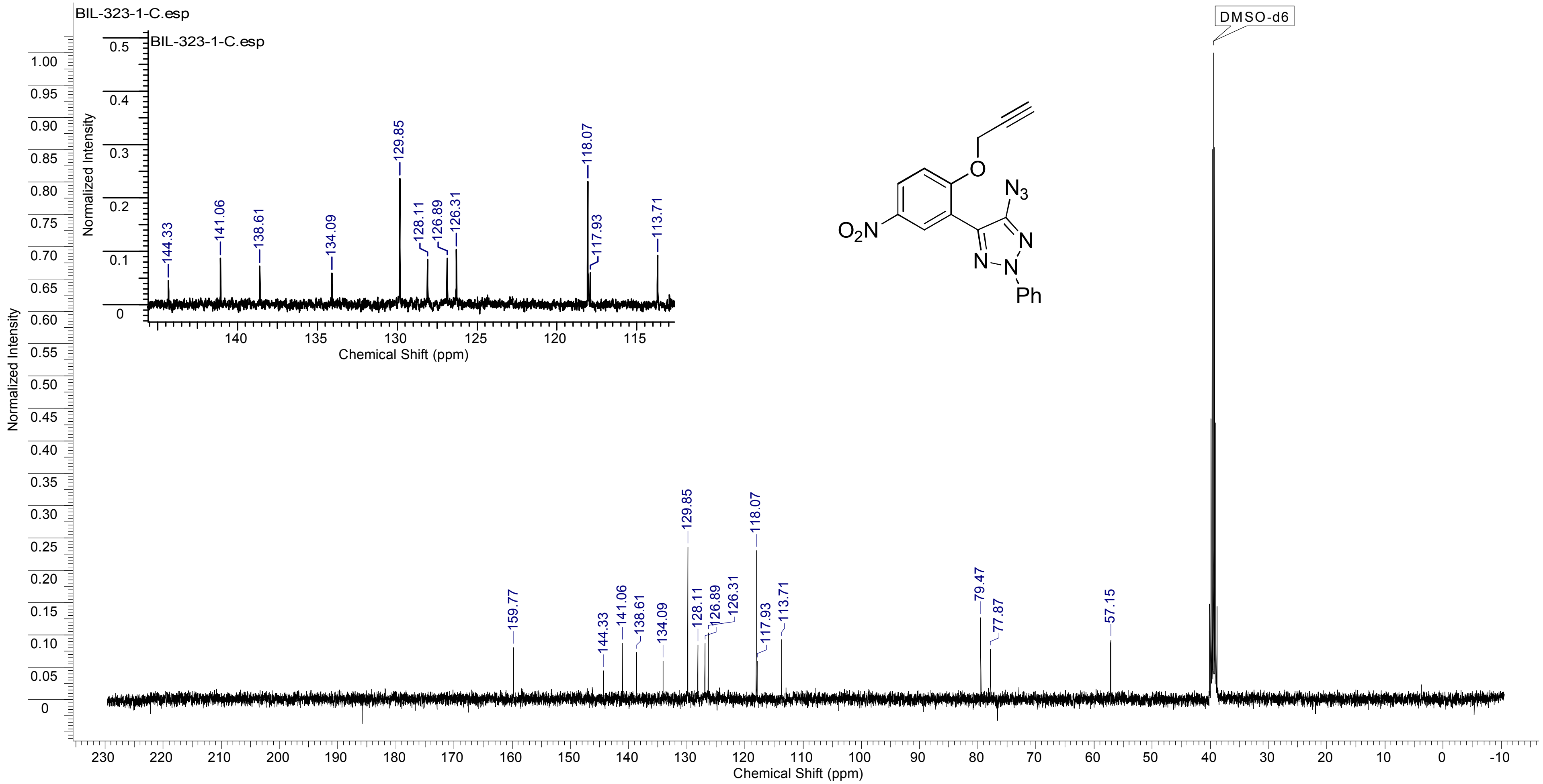

^{13}C NMR spectrum of **3d** (100.6 MHz, $CDCl_3$)

20.11.2019 11:30:06

Acquisition Time (sec)	4.0894	***Comment***	5 mm BBO BB-1H/D Z3918/0123	***Date***	04 Feb 2019 12:33:04
Date Stamp	04 Feb 2019 12:33:04				
File Name				***Frequency (MHz)***	400.13
Nucleus	1H	***Number of Transients***	5	***Origin***	spect
Original Points Count	32768				
Owner	root	***Points Count***	131072	***Pulse Sequence***	zg30
Receiver Gain	128.00				
SW(cyclical) (Hz)	8012.82	***Solvent***	CHLOROFORM-d	***Spectrum Offset (Hz)***	2395.7644
Sweep Width (Hz)	8012.76	***Temperature (degree C)***	27.000		

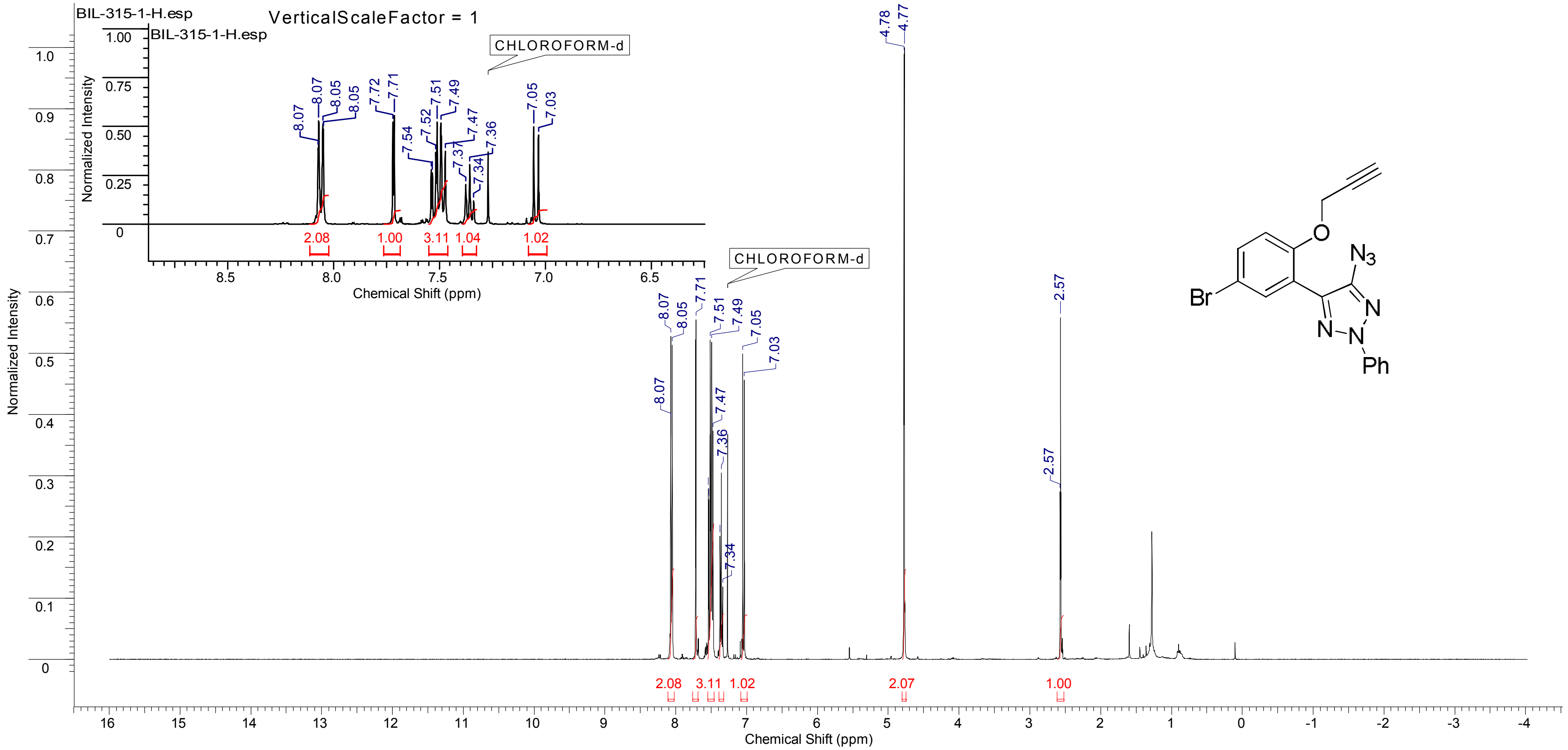

^{1}H NMR spectrum of **3e** (400.1 MHz, CDCl$_3$)

18.04.2019 22:27:20

Acquisition Time (sec)	0.6783	***Comment***	Imported from UXNMR.				***Date***	04 Feb 2019 15:36:32
File Name							***Frequency (MHz)***	100.61
Nucleus	13C	***Number of Transients***	54	***Original Points Count***	16384		***Points Count***	131072
Pulse Sequence	zgpg30	***Solvent***	CHLOROFORM-D	***Spectrum Offset (Hz)***	11060.0088		***Sweep Width (Hz)***	24154.59
Temperature (degree C)	27.000							

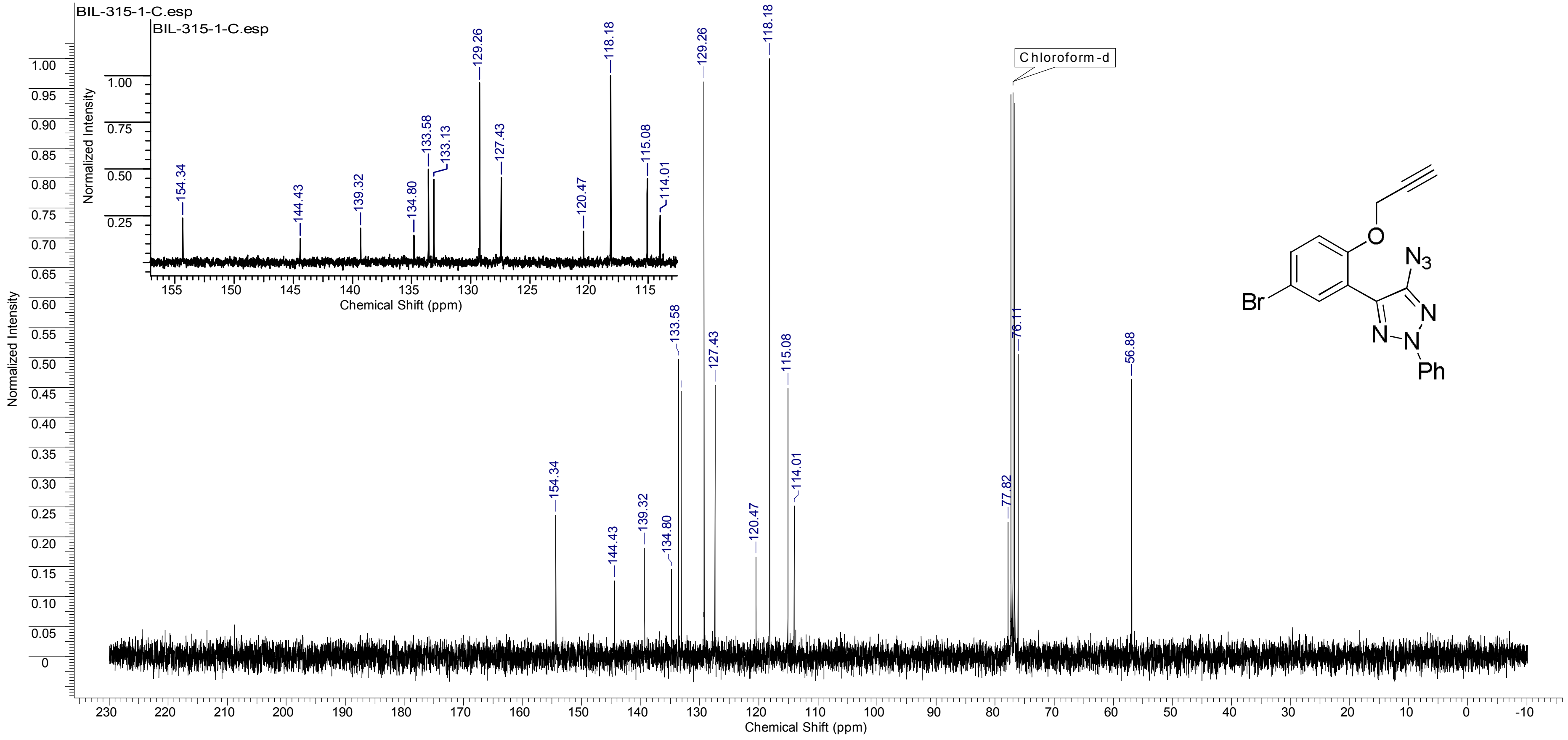

^{13}C NMR spectrum of **3e** (100.6 MHz, $CDCl_3$)

**Acquisition Time (sec)**	2.5559	**Comment**	Imported from UXNMR.			**Date**	07 Apr 2018 13:21:14
**File Name**						**Frequency (MHz)**	400.13
**Nucleus**	1H	**Number of Transients**	4	**Original Points Count**	16384	**Points Count**	65536
**Pulse Sequence**	zg30	**Solvent**	CHLOROFORM-D			**Sweep Width (Hz)**	6410.26
**Temperature (degree C)**	27.000						

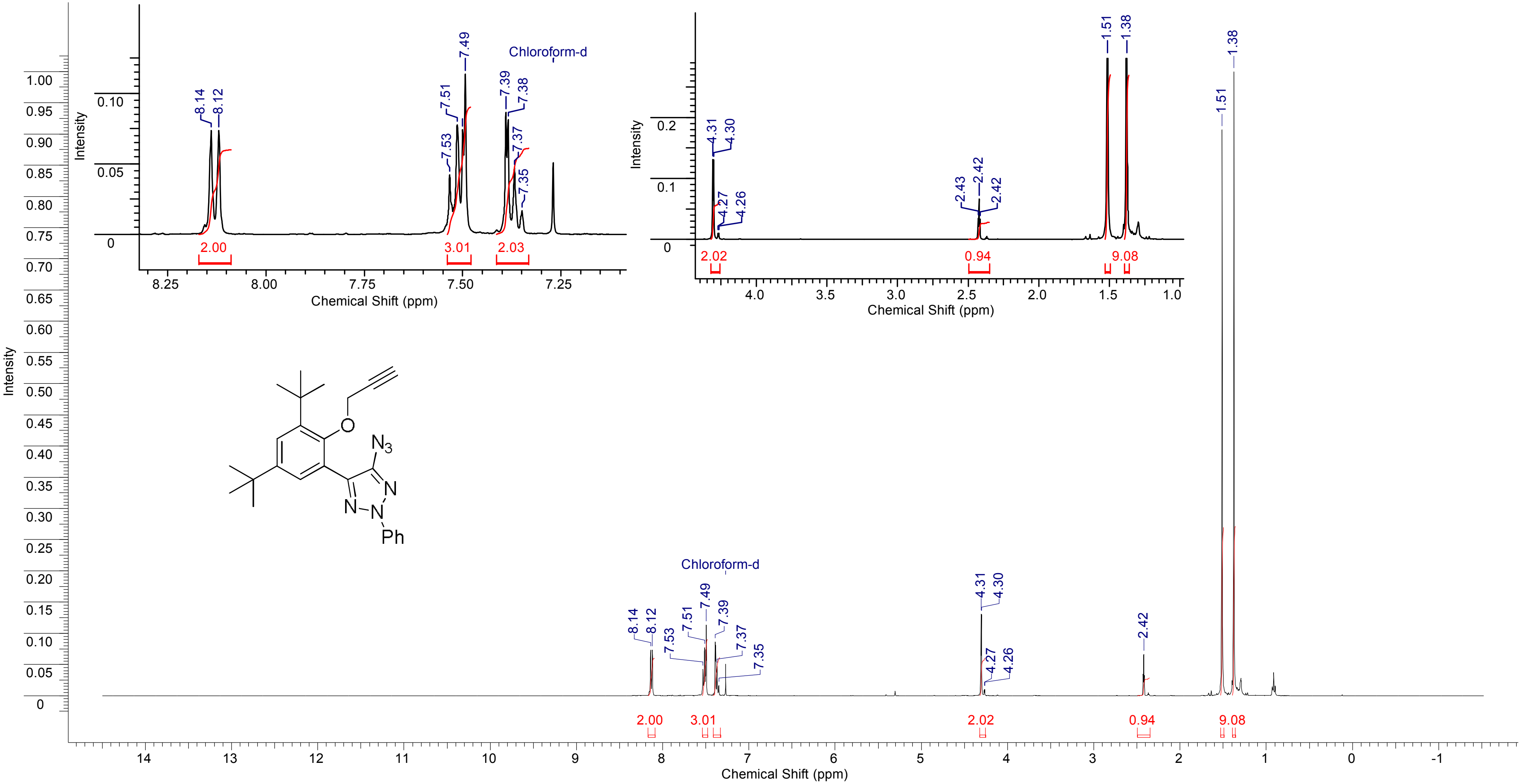

^{1}H NMR spectrum of **3f** (400.1 MHz, $CDCl_3$)

19.04.2019 0:24:36

Acquisition Time (sec)	0.4999	***Comment***	5 mm BBO BB-1H/D Z3918/0123			***Date***	07 Apr 2018 10:22:56
Date Stamp	07 Apr 2018 10:22:56						
File Name						***Frequency (MHz)***	100.61
Nucleus	13C	***Number of Transients***	58	***Origin***	spect	***Original Points Count***	12076
Owner	root	***Points Count***	65536	***Pulse Sequence***	zgpg30	***Receiver Gain***	13004.00
SW(cyclical) (Hz)	24154.59	***Solvent***	CHLOROFORM-d	***Spectrum Offset (Hz)***	11060.0762	***Sweep Width (Hz)***	24154.22
Temperature (degree C)	27.000						

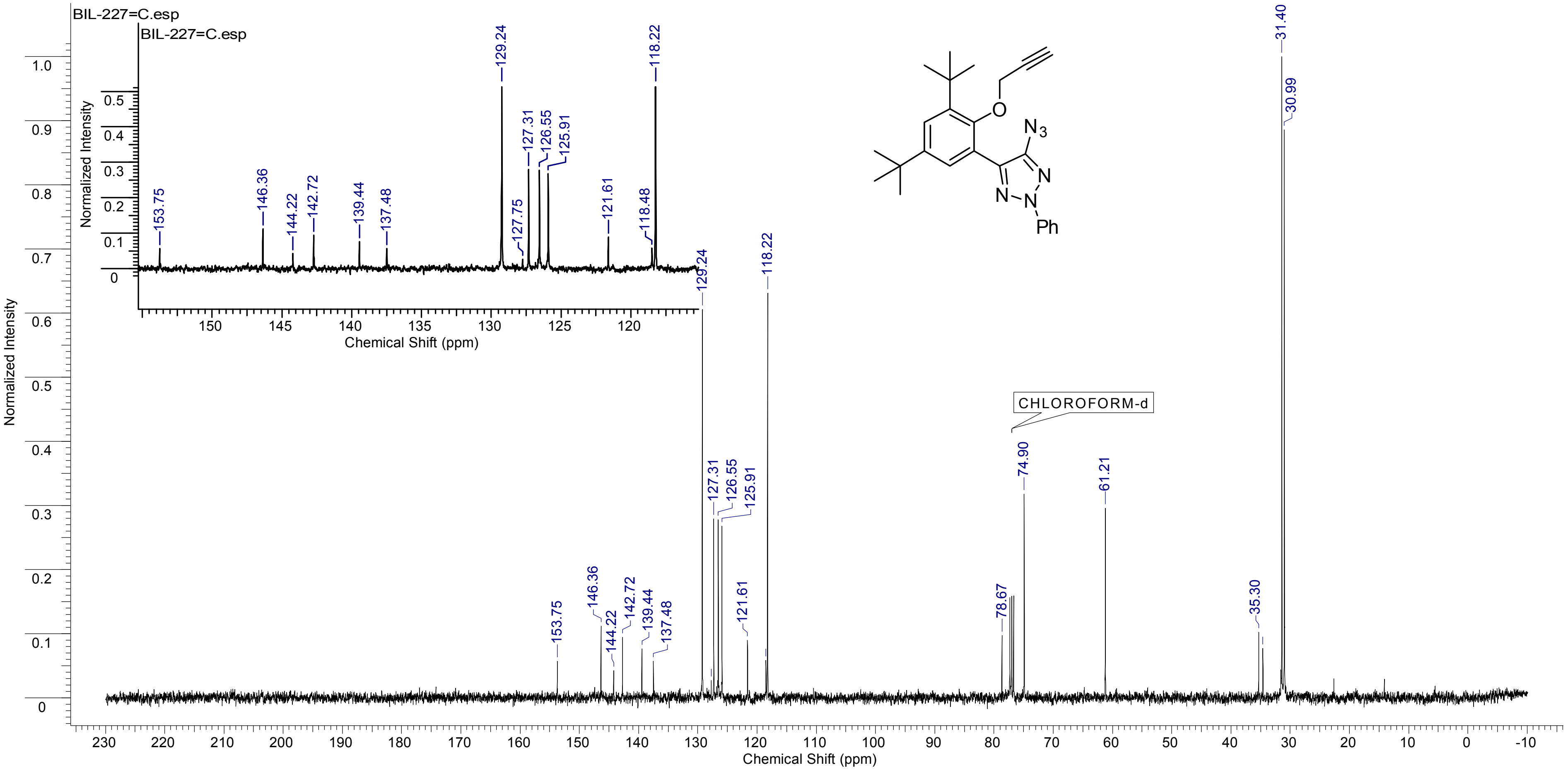

^{13}C NMR spectrum of **3f** (100.6 MHz, $CDCl_3$)

16.03.2019 13:48:46

Acquisition Time (sec)	3.4079	***Date***	Mar 13 2019	***Date Stamp***	Mar 13 2019		
File Name							
Frequency (MHz)	399.96	***Nucleus***	1H	***Number of Transients***	8	***Original Points Count***	16384
Points Count	16384	***Pulse Sequence***	s2pul	***Receiver Gain***	42.00	***Solvent***	CHLOROFORM-d
Spectrum Offset (Hz)	2010.4938	***Sweep Width (Hz)***	4807.69	***Temperature (degree C)***	22.000		

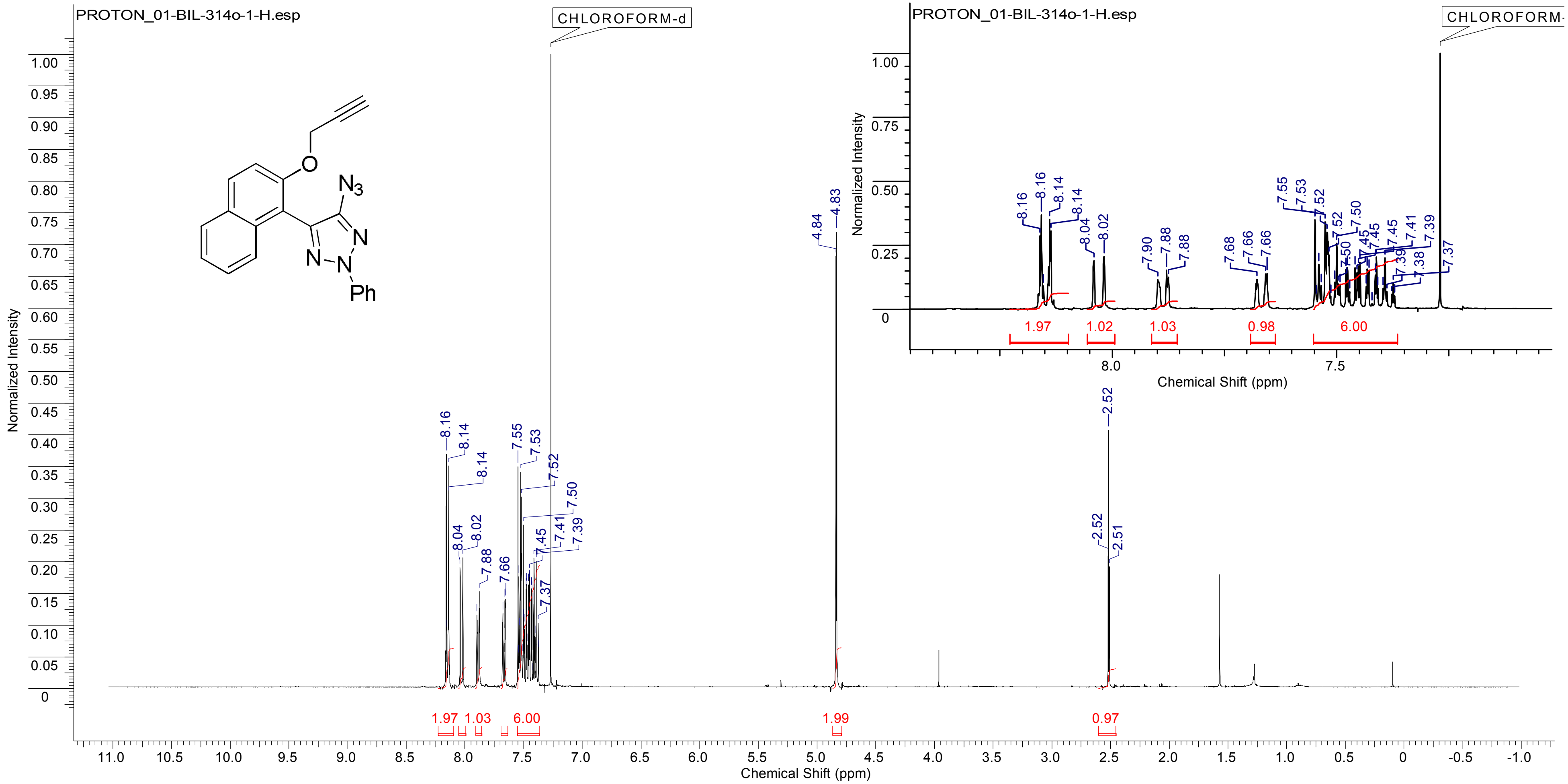

^{1}H NMR spectrum of **3g** (400.1 MHz, $CDCl_3$)

16.03.2019 13:53:21

Acquisition Time (sec)	1.5466	Date	Mar 13 2019	Date Stamp	Mar 13 2019		
File Name							
Frequency (MHz)	100.58	Nucleus	13C	Number of Transients	5000	Original Points Count	32768
Points Count	32768	Pulse Sequence	s2pul	Receiver Gain	60.00	Solvent	CHLOROFORM-d
Spectrum Offset (Hz)	9554.1104	Sweep Width (Hz)	21186.44	Temperature (degree C)	22.000		

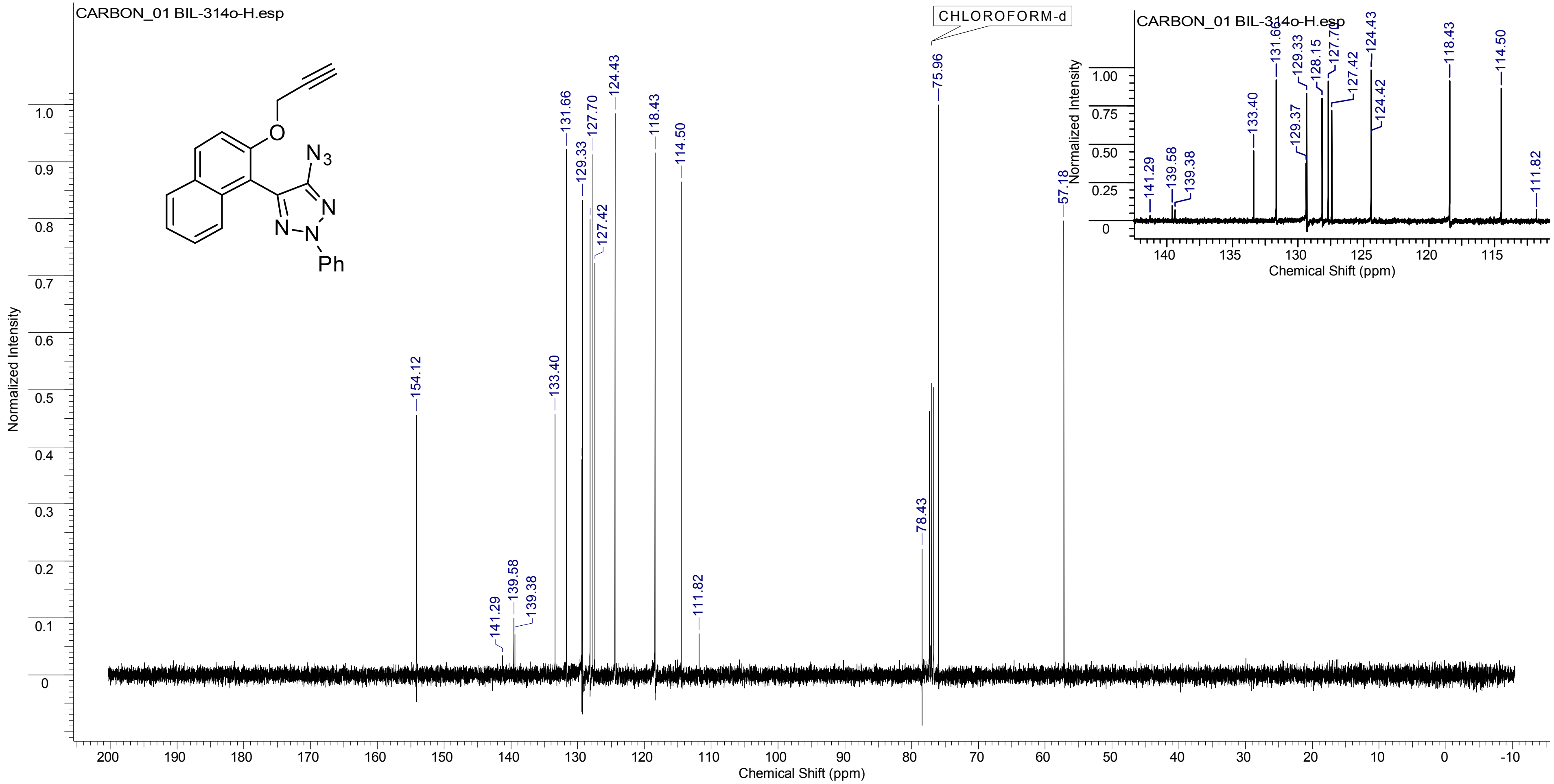

^{13}C NMR spectrum of **3g** (100.6 MHz, $CDCl_3$)

18.06.2019 20:01:10

Acquisition Time (sec)	4.0894	***Comment***	5 mm BBO BB-1H/D Z3918/0123			***Date***	14 May 2019 11:09:52
Date Stamp	14 May 2019 11:09:52						
File Name						***Frequency (MHz)***	400.13
Nucleus	1H	***Number of Transients***	4	***Origin***	spect	***Original Points Count***	32768
Owner	root	***Points Count***	131072	***Pulse Sequence***	zg30	***Receiver Gain***	45.30
SW(cyclical) (Hz)	8012.82	***Solvent***	CHLOROFORM-d			***Spectrum Offset (Hz)***	2396.0703
Sweep Width (Hz)	8012.76	***Temperature (degree C)***	27.000				

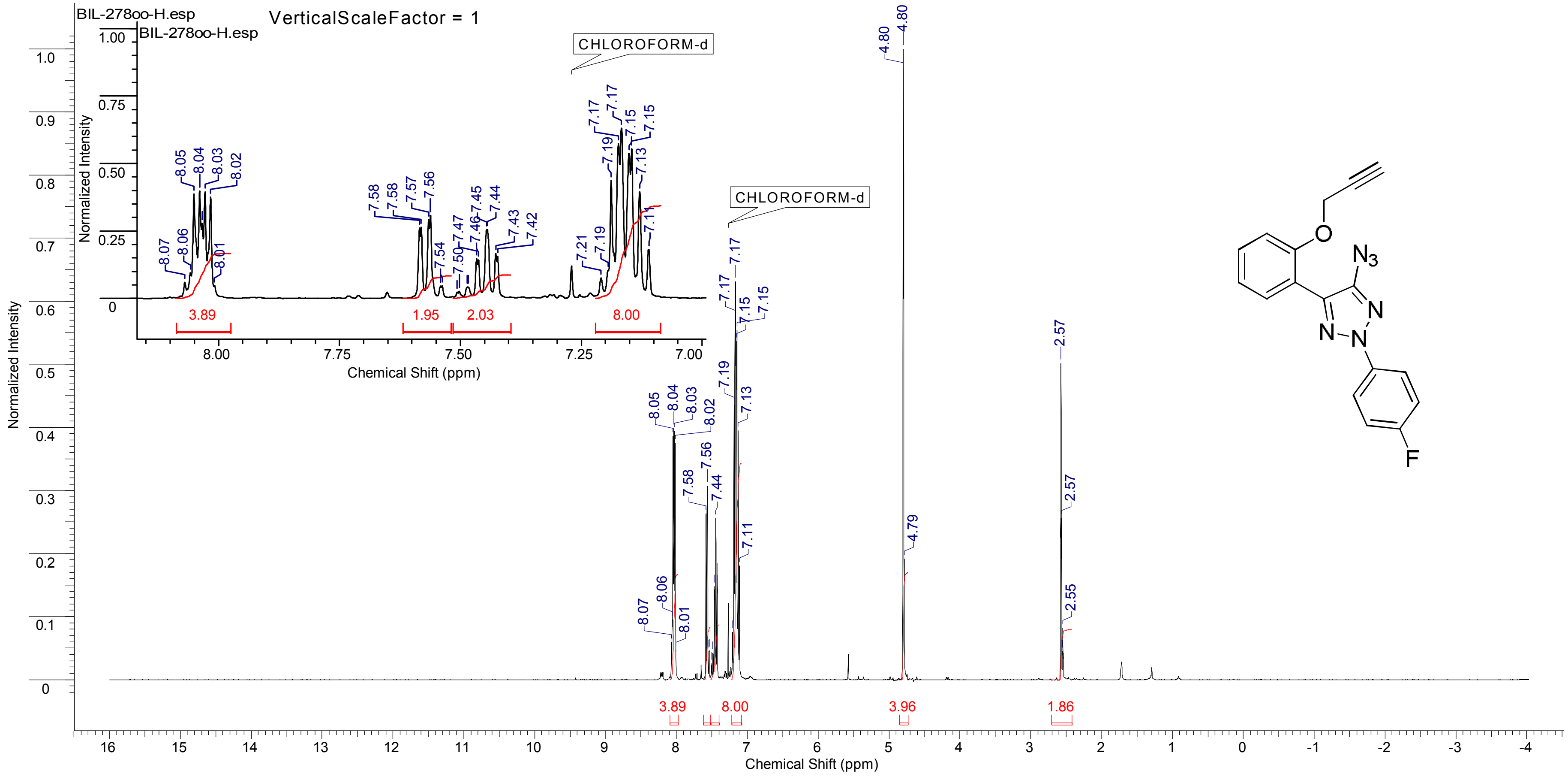

^{1}H NMR spectrum of **3h** (400.1 MHz, $CDCl_3$)

18.06.2019 14:59:11

Acquisition Time (sec)	0.6783	***Comment***	5 mm BBO BB-1H/D Z3918/0123			***Date***	14 May 2019 11:14:08
Date Stamp	14 May 2019 11:14:08						
File Name						***Frequency (MHz)***	100.61
Nucleus	13C	***Number of Transients***	233	***Origin***	spect	***Original Points Count***	16384
Owner	root	***Points Count***	131072	***Pulse Sequence***	zgpg30	***Receiver Gain***	13004.00
SW(cyclical) (Hz)	24154.59	***Solvent***	CHLOROFORM-d	***Spectrum Offset (Hz)***	11053.9023	***Sweep Width (Hz)***	24154.41
Temperature (degree C)	27.000						

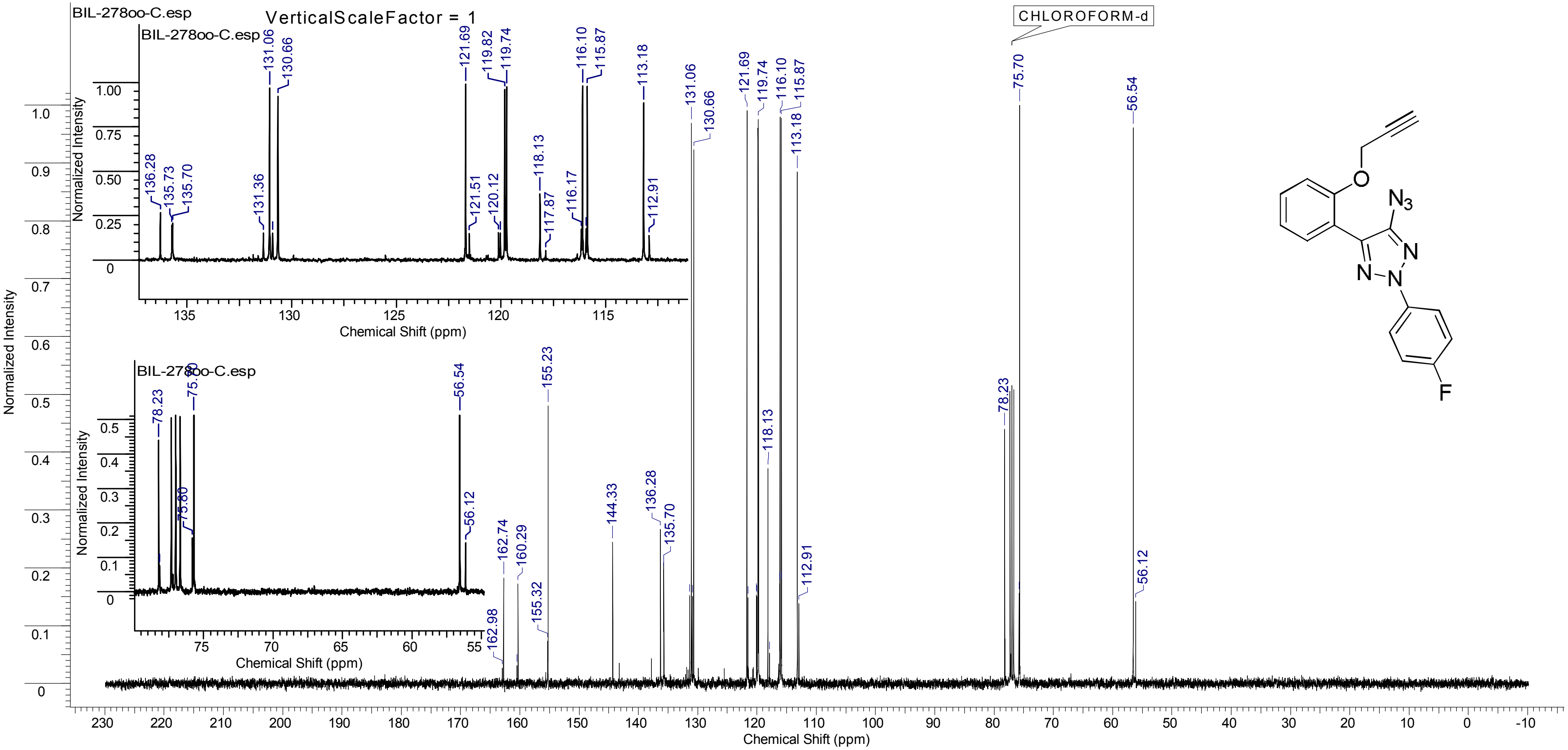

^{13}C NMR spectrum of **3h** (100.6 MHz, $CDCl_3$)

19.04.2019 17:11:30

Acquisition Time (sec)	4.0894	***Comment***	5 mm BBO BB-1H/D Z3918/0123			***Date***	27 Mar 2019 14:28:16
Date Stamp	27 Mar 2019 14:28:16						
File Name						***Frequency (MHz)***	400.13
Nucleus	1H	***Number of Transients***	4	***Origin***	spect	***Original Points Count***	32768
Owner	root	***Points Count***	131072	***Pulse Sequence***	zg30	***Receiver Gain***	228.10
SW(cyclical) (Hz)	8012.82	***Solvent***	CHLOROFORM-d			***Spectrum Offset (Hz)***	2395.7644
Sweep Width (Hz)	8012.76	***Temperature (degree C)***	27.000				

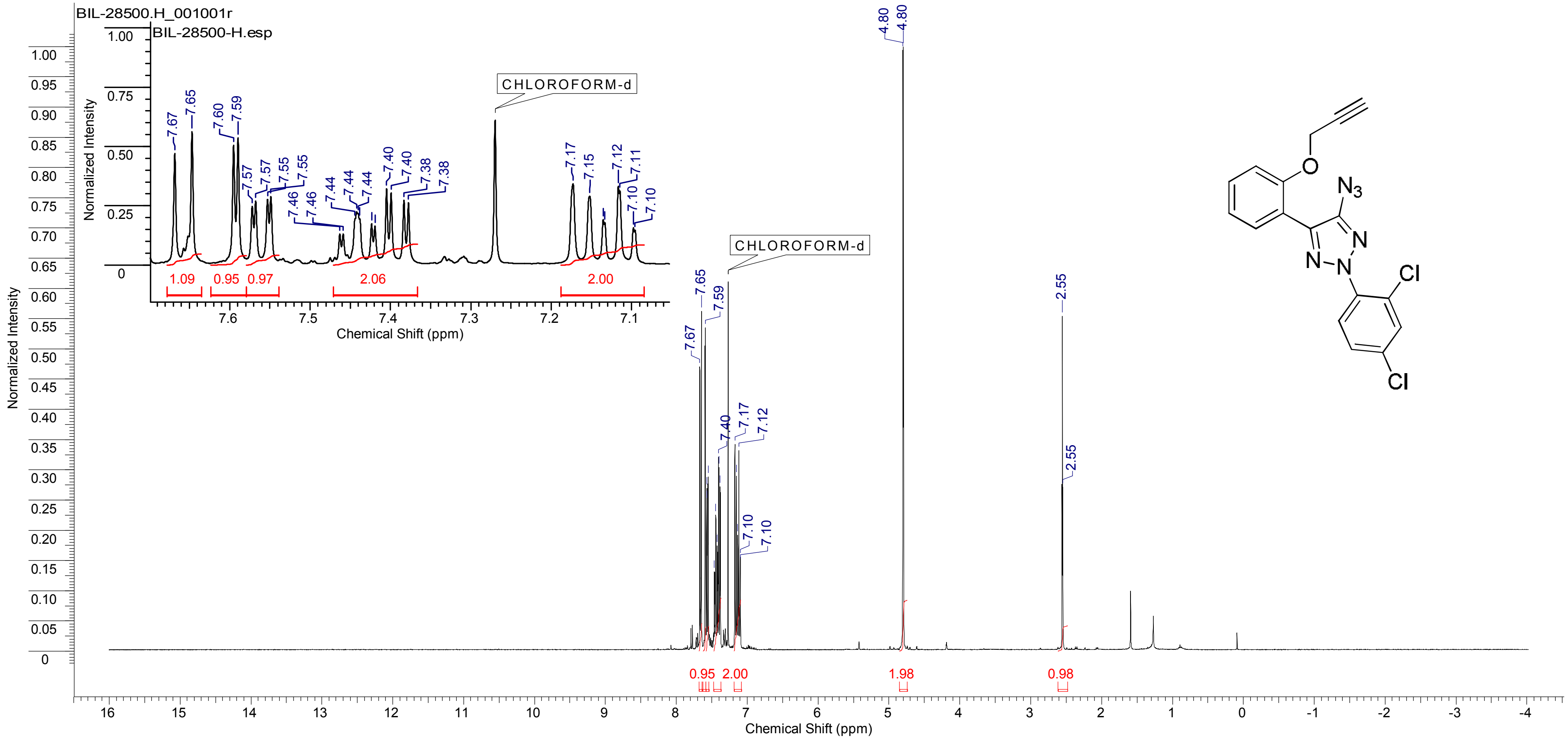

^{1}H NMR spectrum of **3i** (400.1 MHz, $CDCl_3$)

19.04.2019 17:12:45

**Acquisition Time (sec)**	0.6783	**Comment**	5 mm BBO BB-1H/D Z3918/0123			**Date**	27 Mar 2019 14:30:24
**Date Stamp**	27 Mar 2019 14:30:24						
**File Name**						**Frequency (MHz)**	100.61
**Nucleus**	13C	**Number of Transients**	234	**Origin**	spect	**Original Points Count**	16384
**Owner**	root	**Points Count**	131072	**Pulse Sequence**	zgpg30	**Receiver Gain**	16384.00
**SW(cyclical) (Hz)**	24154.59	**Solvent**	CHLOROFORM-d	**Spectrum Offset (Hz)**	11062.5635	**Sweep Width (Hz)**	24154.41
**Temperature (degree C)**	27.000						

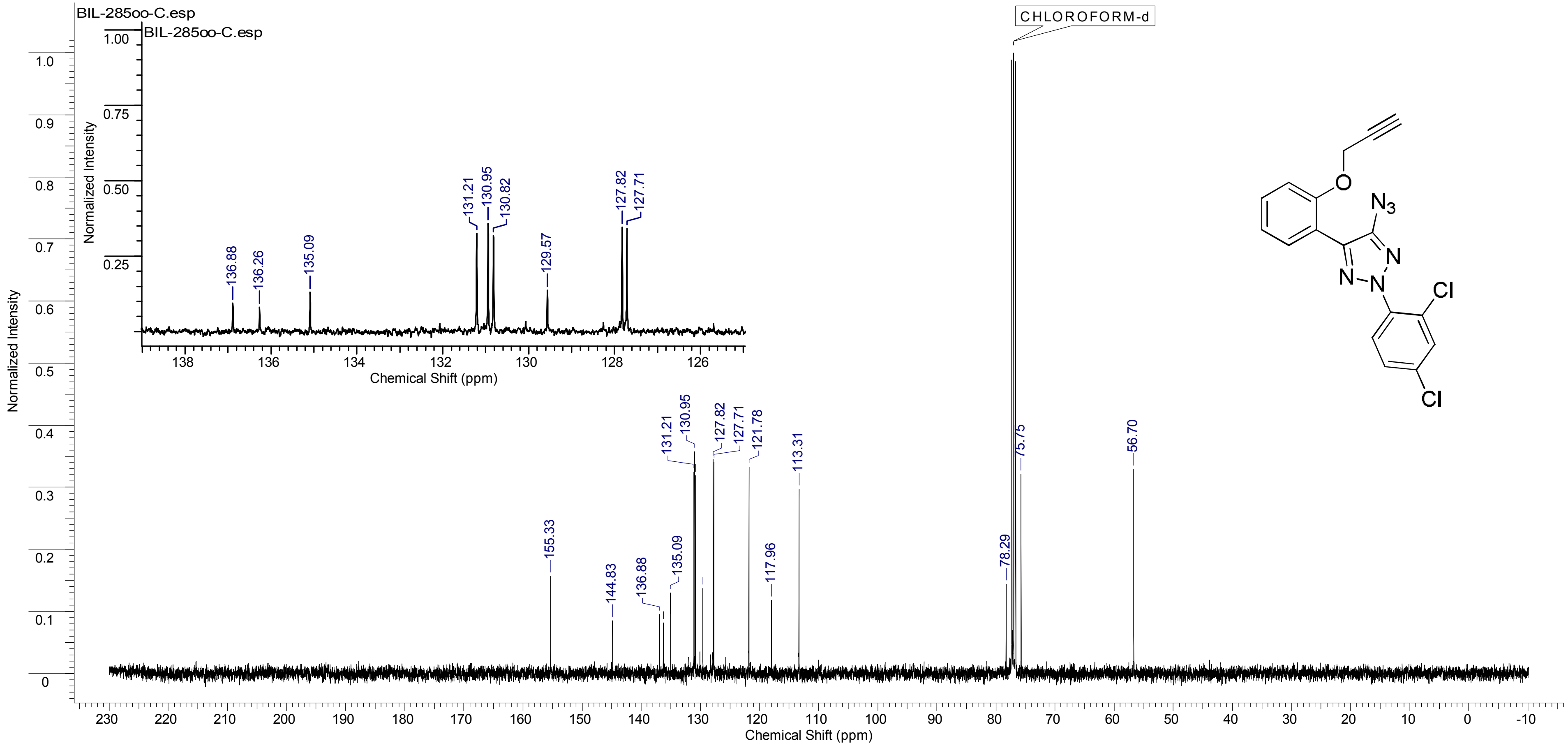

^{13}C NMR spectrum of **3i** (100.6 MHz, $CDCl_3$)

21.04.2019 17:12:41

Acquisition Time (sec)	4.0894	***Comment***	Imported from UXNMR.			***Date***	09 Apr 2019 14:04:20
File Name						***Frequency (MHz)***	400.13
Nucleus	1H	***Number of Transients***	4	***Original Points Count***	32768	***Points Count***	131072
Pulse Sequence	zg30	***Solvent***	CHLOROFORM-d			***Spectrum Offset (Hz)***	2395.8215
Sweep Width (Hz)	8012.82	***Temperature (degree C)***	27.000				

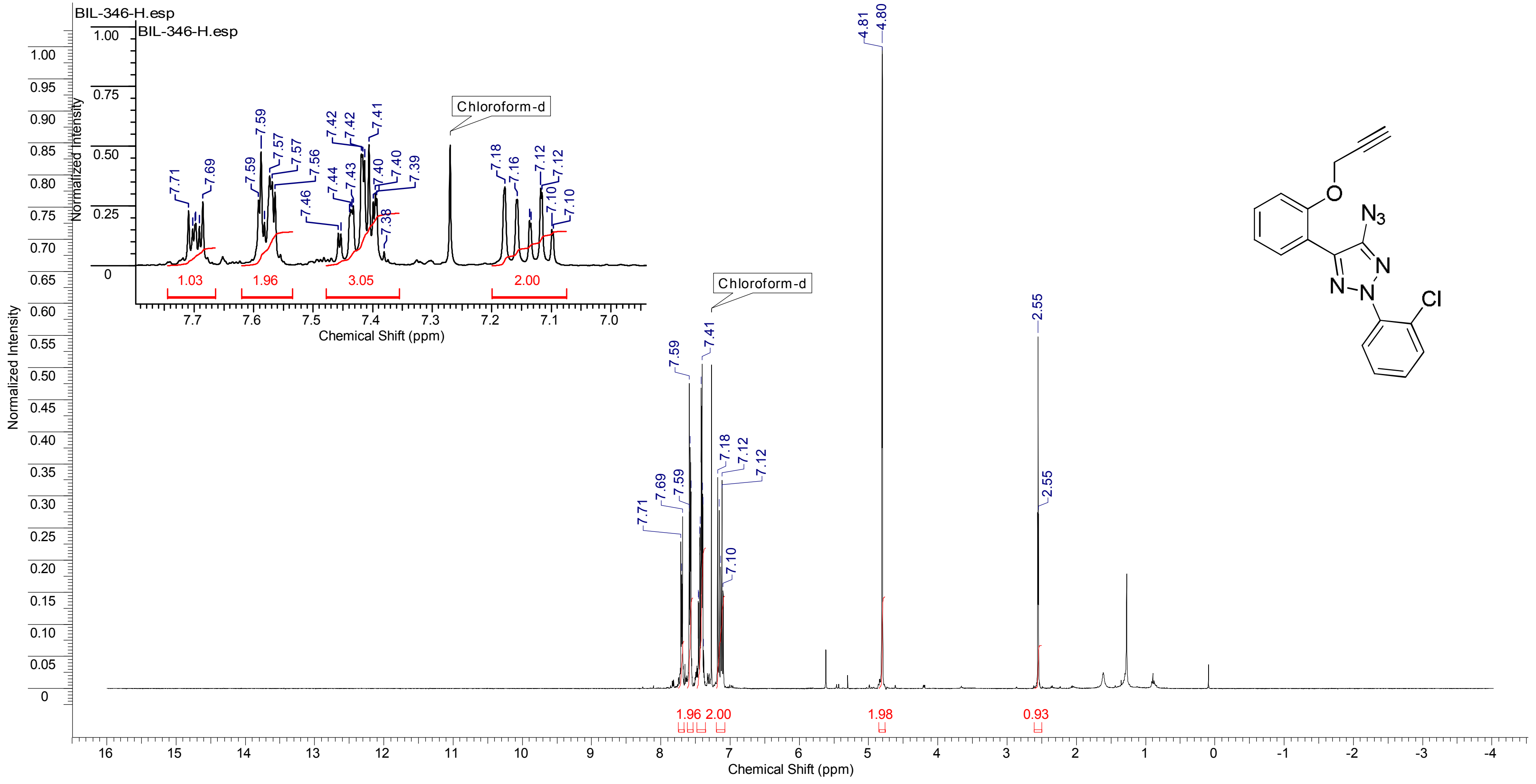

^{1}H NMR spectrum of **3j** (400.1 MHz, $CDCl_3$)

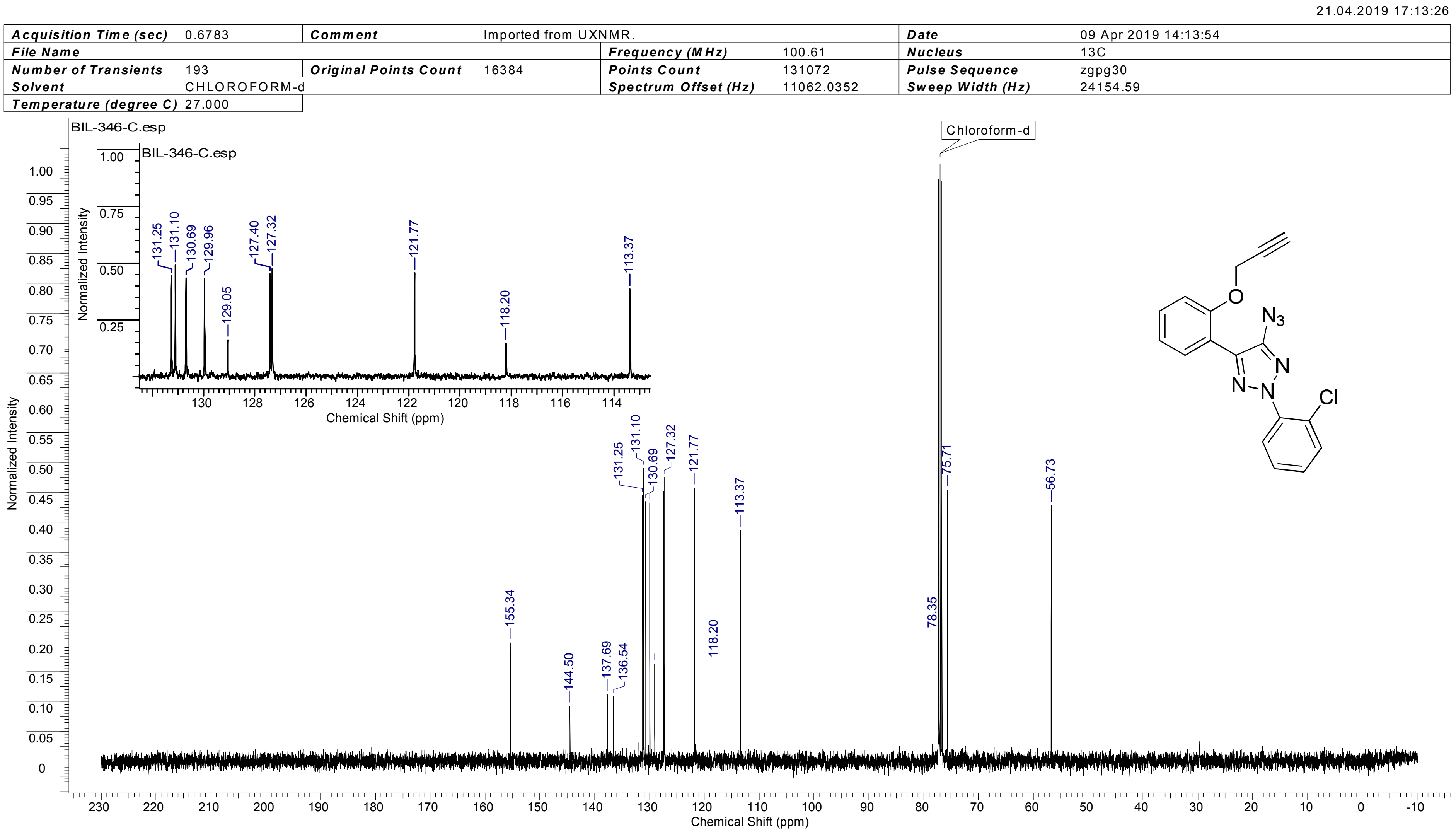

^{13}C NMR spectrum of **3j** (100.6 MHz, $CDCl_3$)

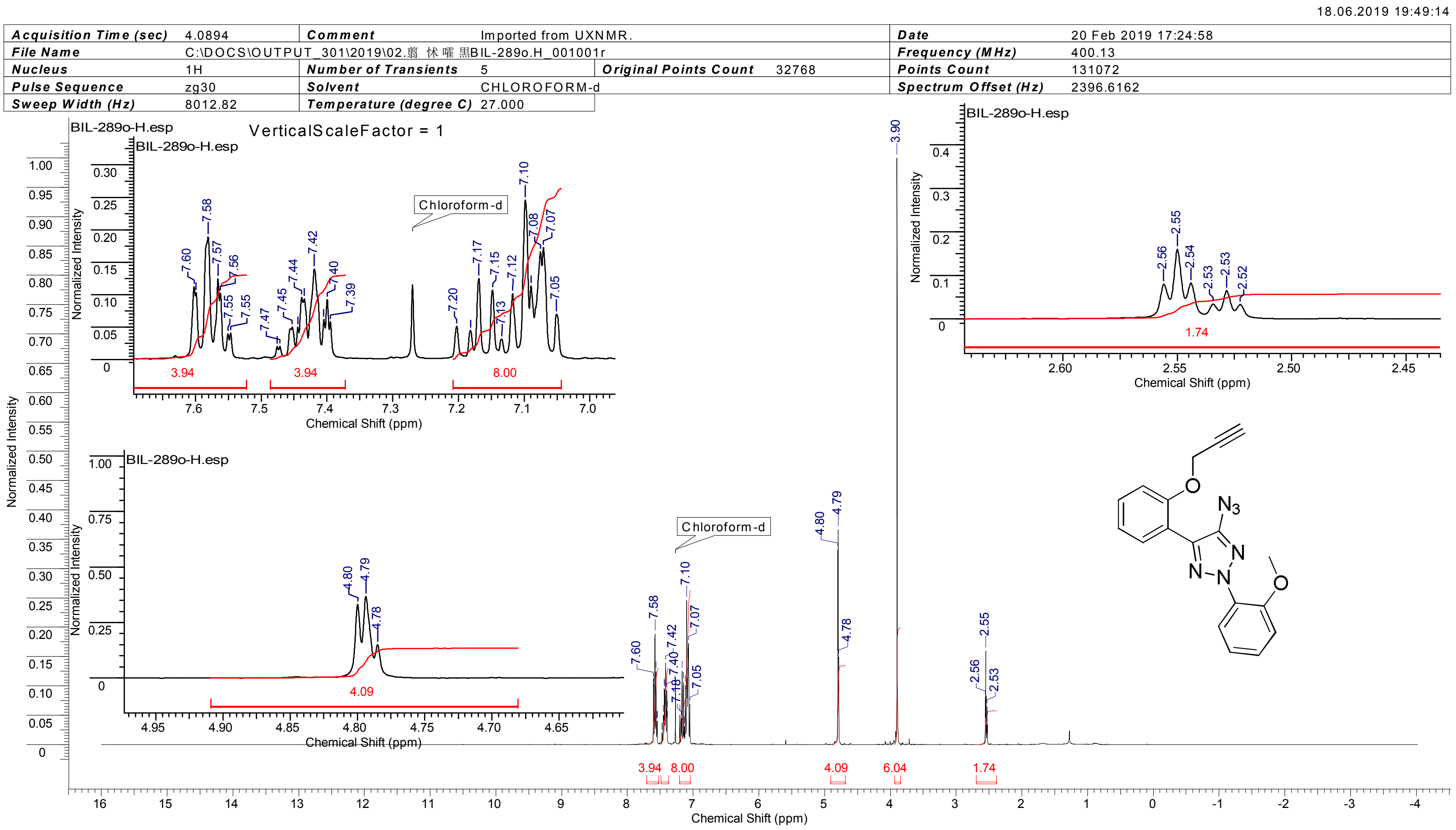

^1H NMR spectrum of **3k** (400.1 MHz, $CDCl_3$)

18.06.2019 16:31:06

Acquisition Time (sec)	0.6783	***Comment***	5 mm Dual 13C/1H Z3756/0200			***Date***	20 Feb 2019 14:26:08
Date Stamp	20 Feb 2019 14:26:08						
File Name						***Frequency (MHz)***	100.61
Nucleus	13C	***Number of Transients***	121	***Origin***	spect	***Original Points Count***	16384
Owner	root	***Points Count***	131072	***Pulse Sequence***	zgpg30	***Receiver Gain***	14596.50
SW(cyclical) (Hz)	24154.59	***Solvent***	CHLOROFORM-d	***Spectrum Offset (Hz)***	11059.0625	***Sweep Width (Hz)***	24154.41
Temperature (degree C)	27.000						

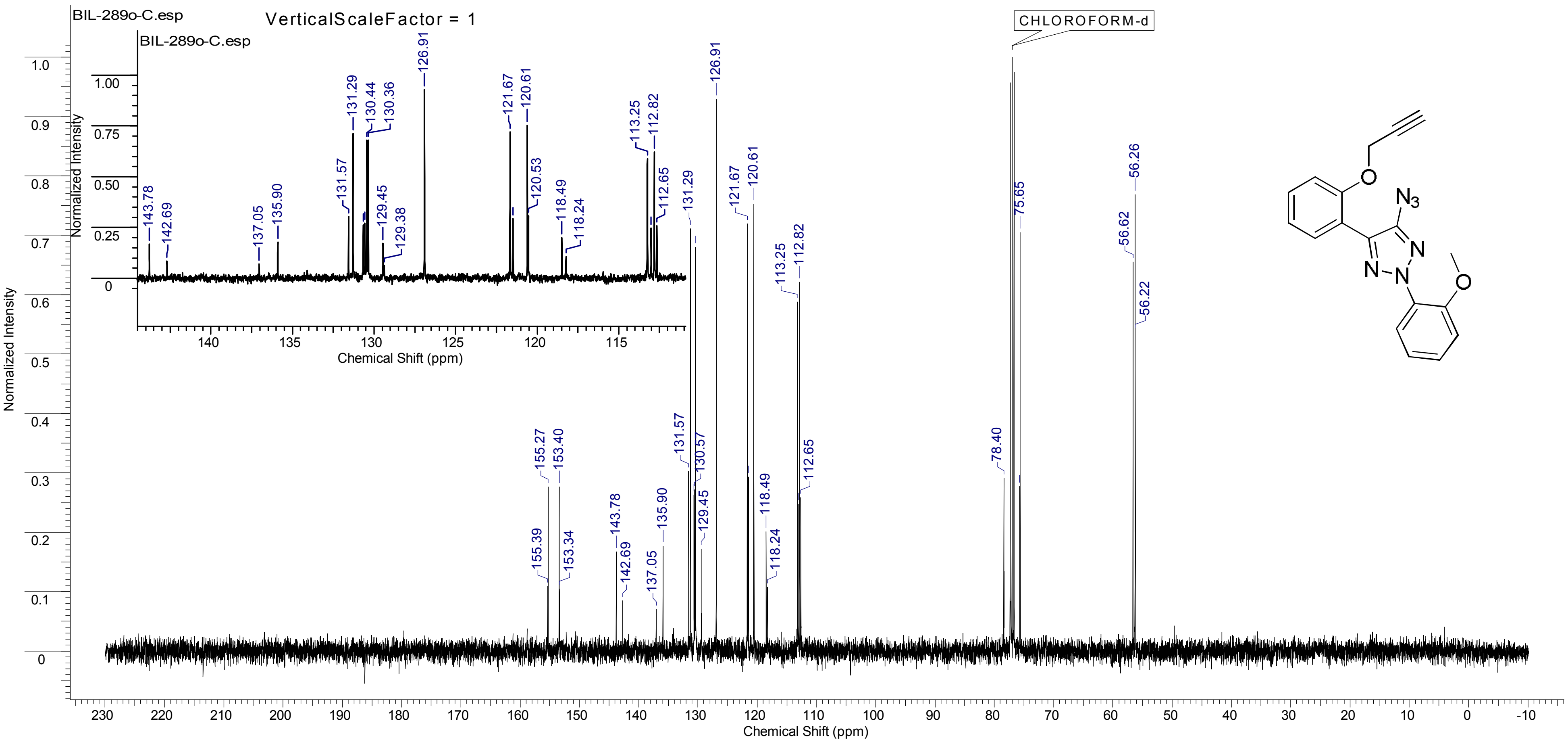

^{13}C NMR spectrum of **3k** (100.6 MHz, $CDCl_3$)

18.06.2019 20:26:37

Acquisition Time (sec)	4.0894	***Comment***	5 mm Dual 13C/1H Z3756/0200			***Date***	28 Feb 2019 08:06:24
Date Stamp	28 Feb 2019 08:06:24						
File Name						***Frequency (MHz)***	400.13
Nucleus	1H	***Number of Transients***	4	***Origin***	spect	***Original Points Count***	32768
Owner	root	***Points Count***	131072	***Pulse Sequence***	zg30	***Receiver Gain***	28.50
SW(cyclical) (Hz)	8012.82	***Solvent***	CHLOROFORM-d			***Spectrum Offset (Hz)***	2397.4763
Sweep Width (Hz)	8012.76	***Temperature (degree C)***	27.000				

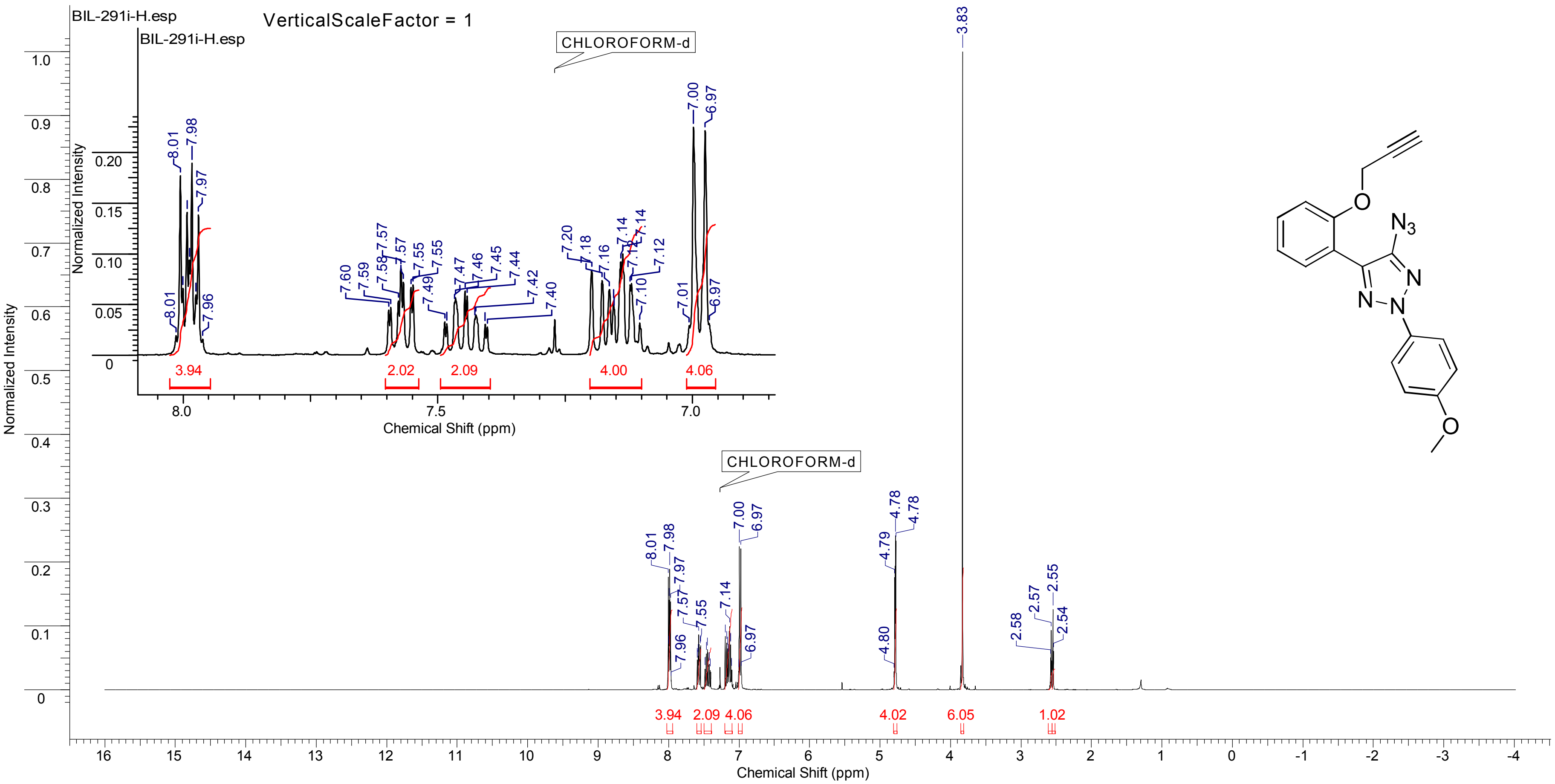

^{1}H NMR spectrum of **3l** (400.1 MHz, $CDCl_3$)

18.06.2019 20:35:34

Acquisition Time (sec)	0.6783	***Comment***	5 mm Dual 13C/1H Z3756/0200			***Date***	28 Feb 2019 08:08:32
Date Stamp	28 Feb 2019 08:08:32						
File Name						***Frequency (MHz)***	100.61
Nucleus	13C	***Number of Transients***	64	***Origin***	spect	***Original Points Count***	16384
Owner	root	***Points Count***	131072	***Pulse Sequence***	zgpg30	***Receiver Gain***	7298.20
SW(cyclical) (Hz)	24154.59	***Solvent***	CHLOROFORM-d	***Spectrum Offset (Hz)***	11047.6367	***Sweep Width (Hz)***	24154.41
Temperature (degree C)	27.000						

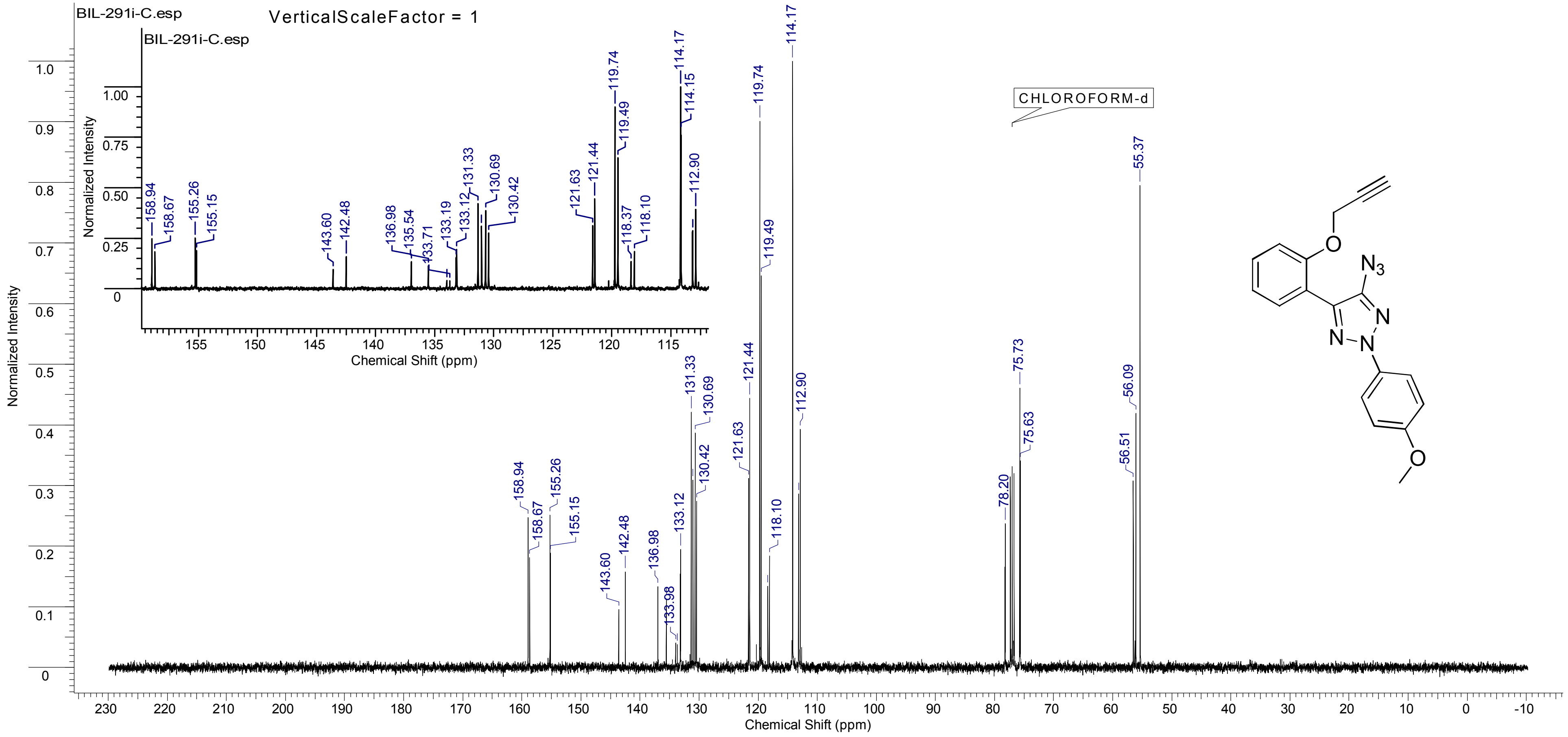

^{13}C NMR spectrum of **3l** (100.6 MHz, $CDCl_3$)

**Acquisition Time (sec)**	4.0894	**Comment**	Imported from UXNMR.			**Date**	06 Feb 2019 17:09:16
**File Name**						**Frequency (MHz)**	400.13
**Nucleus**	1H	**Number of Transients**	5	**Original Points Count**	32768	**Points Count**	131072
**Pulse Sequence**	zg30	**Solvent**	CHLOROFORM-d			**Spectrum Offset (Hz)**	2395.8215
**Sweep Width (Hz)**	8012.82	**Temperature (degree C)**	27.000				

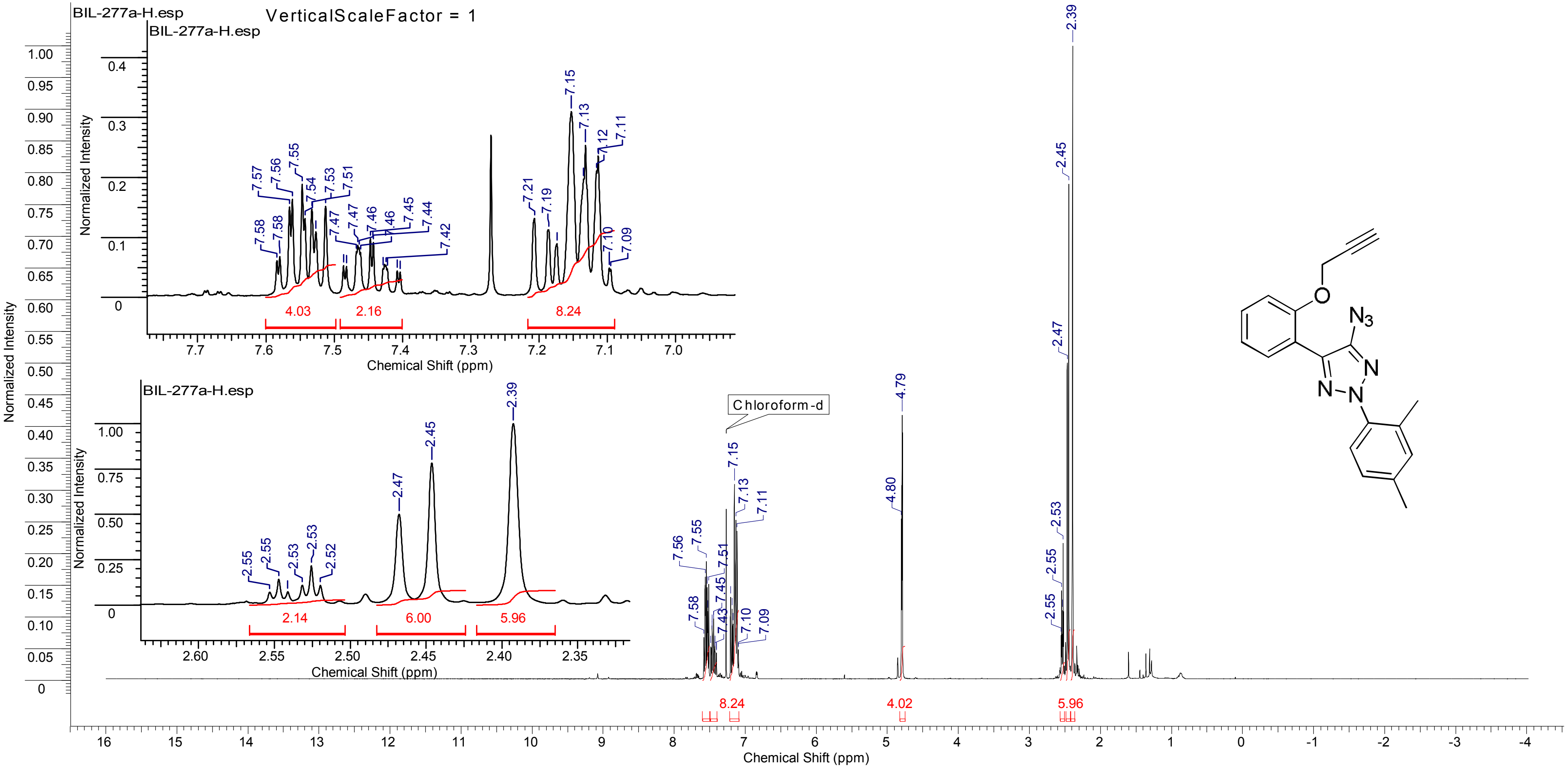

^{1}H NMR spectrum of **3m** (400.1 MHz, $CDCl_3$)

18.06.2019 22:09:45

Acquisition Time (sec)	0.6783	***Comment***	5 mm BBO BB-1H/D Z3918/0123			***Date***	06 Feb 2019 14:15:28		
Date Stamp	06 Feb 2019 14:15:28			***File Name***				***Frequency (MHz)***	100.61
Nucleus	13C	***Number of Transients***	124	***Origin***	spect	***Original Points Count***	16384	***Owner***	root
Points Count	131072	***Pulse Sequence***	zgpg30	***Receiver Gain***	13004.00	***SW(cyclical) (Hz)***	24154.59		
Solvent	CHLOROFORM-d			***Spectrum Offset (Hz)***	11061.0898	***Sweep Width (Hz)***	24154.41	***Temperature (degree C)***	27.000

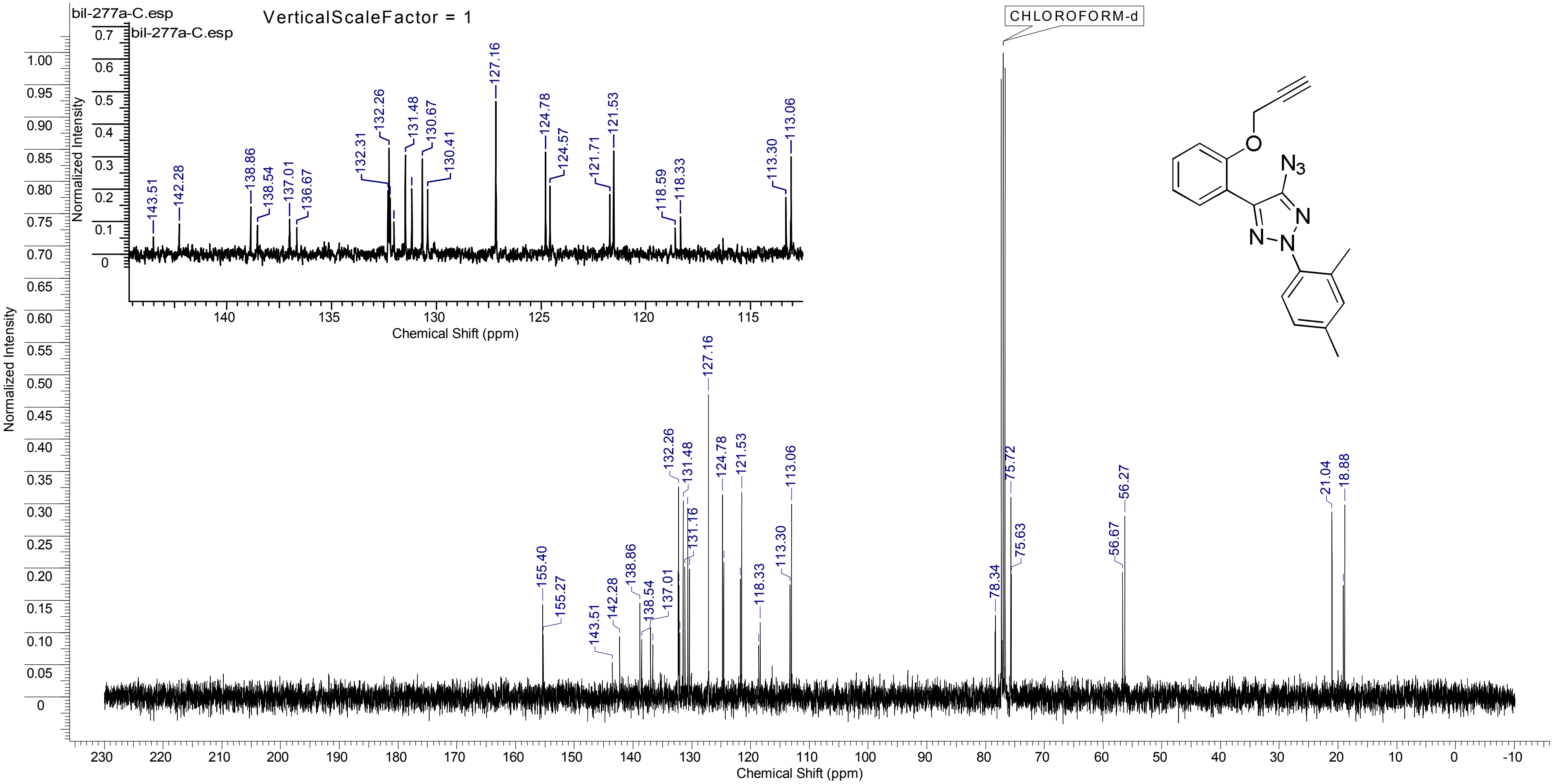

^{13}C NMR spectrum of **3m** (100.6 MHz, $CDCl_3$)

17.06.2019 17:02:28

Acquisition Time (sec)	4.0894	Comment	5 mm BBO BB-1H/D Z3918/0123	Date	13 May 2019 12:18:08
Date Stamp	13 May 2019 12:18:08				
File Name				Frequency (MHz)	400.13
Nucleus	1H	Number of Transients	4	Origin: spect	Original Points Count: 32768
Owner	root	Points Count	131072	Pulse Sequence: zg30	Receiver Gain: 114.00
SW(cyclical) (Hz)	8012.82	Solvent	CHLOROFORM-d	Spectrum Offset (Hz)	2395.8254
Sweep Width (Hz)	8012.76	Temperature (degree C)	27.000		

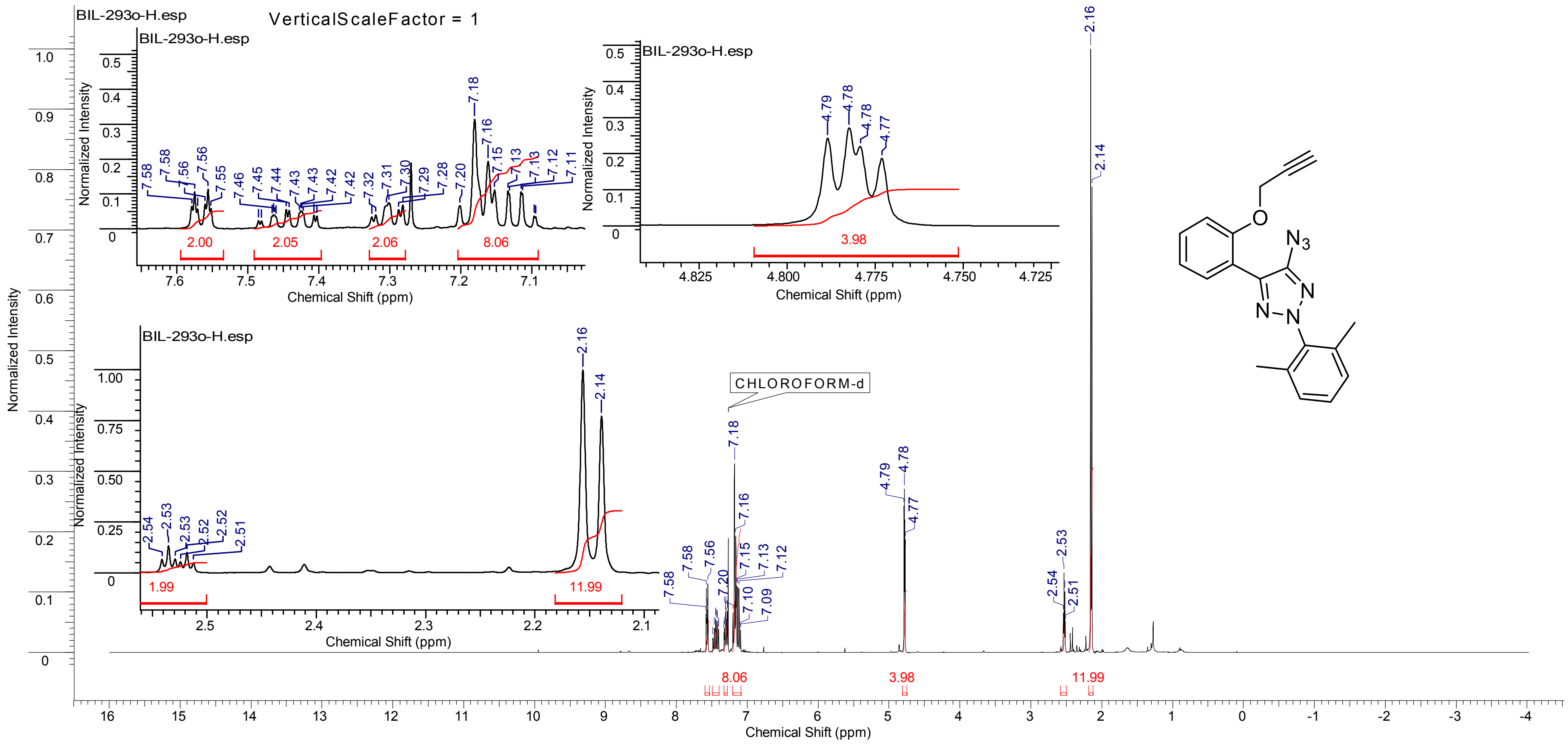

^{1}H NMR spectrum of **3n** (400.1 MHz, $CDCl_3$)

17.06.2019 17:11:26

**Acquisition Time (sec)**	0.6783	**Comment**	5 mm BBO BB-1H/D Z3918/0123			**Date**	13 May 2019 12:20:16
**Date Stamp**	13 May 2019 12:20:16						
**File Name**						**Frequency (MHz)**	100.61
**Nucleus**	13C	**Number of Transients**	217	**Origin**	spect	**Original Points Count**	16384
**Owner**	root	**Points Count**	131072	**Pulse Sequence**	zgpg30	**Receiver Gain**	13004.00
**SW(cyclical) (Hz)**	24154.59	**Solvent**	CHLOROFORM-d	**Spectrum Offset (Hz)**	11060.1680	**Sweep Width (Hz)**	24154.41
**Temperature (degree C)**	27.000						

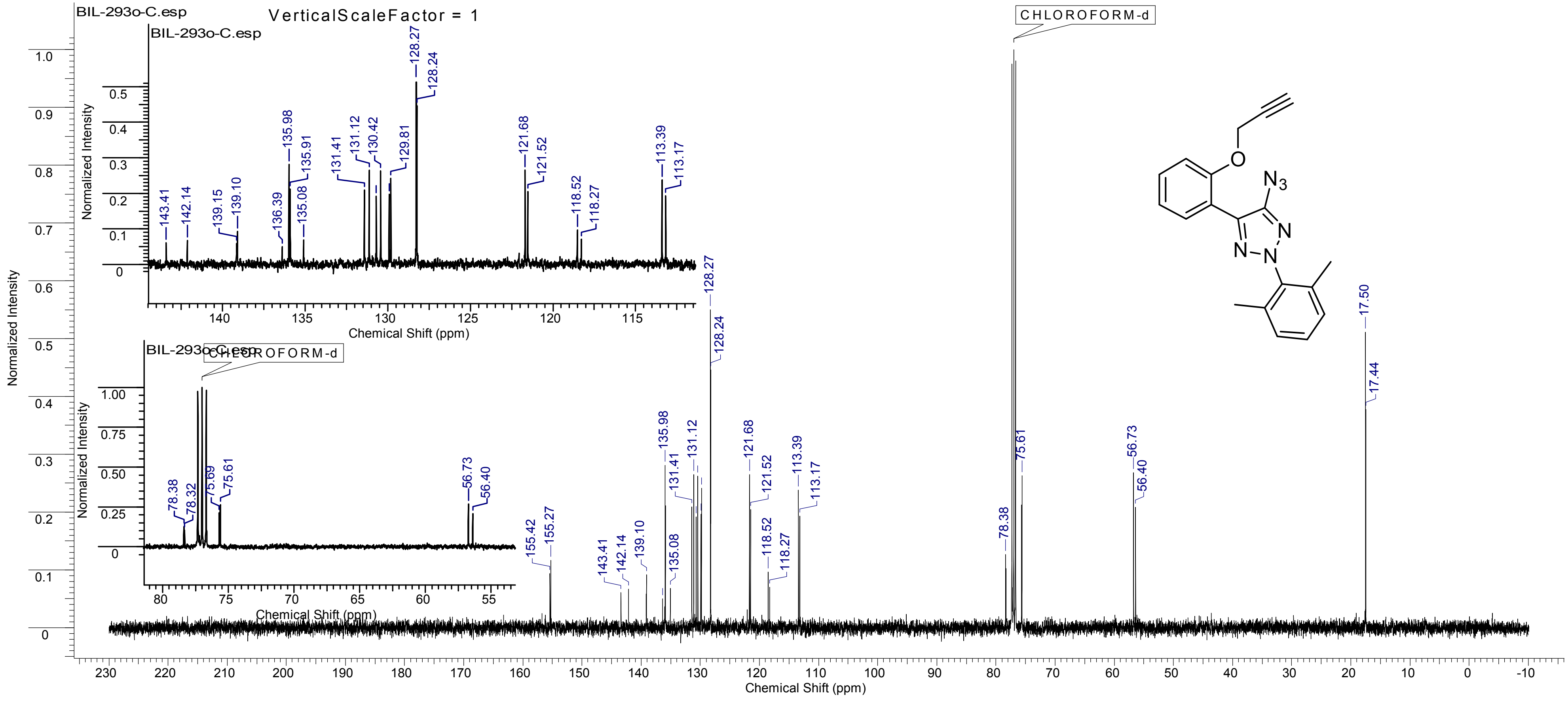

^{13}C NMR spectrum of **3n** (100.6 MHz, $CDCl_3$)

22.04.2019 0:37:08

**Acquisition Time (sec)**	4.0894	**Comment**	5 mm BBO BB-1H/D Z3918/0123			**Date**	06 Mar 2019 14:24:00		
**Date Stamp**	06 Mar 2019 14:24:00			**File Name**				**Frequency (MHz)**	400.13
**Nucleus**	1H	**Number of Transients**	4	**Origin**	spect	**Original Points Count**	32768	**Owner**	root
**Points Count**	131072	**Pulse Sequence**	zg30	**Receiver Gain**	203.20	**SW(cyclical) (Hz)**	8012.82	**Solvent**	CHLOROFORM-d
**Spectrum Offset (Hz)**	2395.8254	**Sweep Width (Hz)**	8012.76	**Temperature (degree C)**	27.000				

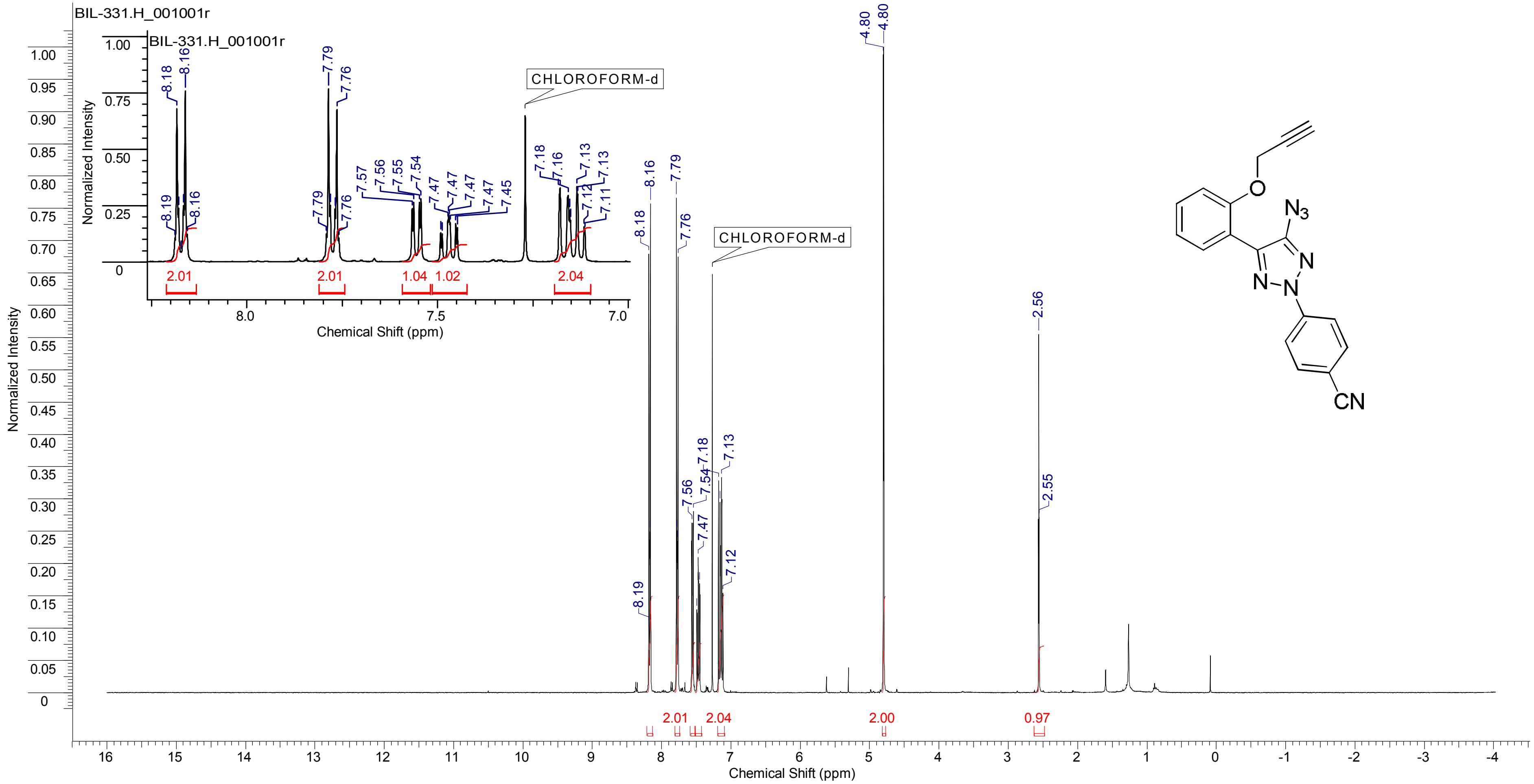

^{1}H NMR spectrum of **3o** (400.1 MHz, $CDCl_3$)

22.04.2019 0:38:16

Acquisition Time (sec)	0.6783	***Comment***	5 mm BBO BB-1H/D Z3918/0123			***Date***	06 Mar 2019 14:26:08		
Date Stamp	06 Mar 2019 14:26:08			***File Name***				***Frequency (MHz)***	100.61
Nucleus	13C	***Number of Transients***	212	***Origin***	spect	***Original Points Count***	16384	***Owner***	root
Points Count	131072	***Pulse Sequence***	zgpg30	***Receiver Gain***	16384.00	***SW(cyclical) (Hz)***	24154.59	***Solvent***	CHLOROFORM-d
Spectrum Offset (Hz)	11062.1953	***Sweep Width (Hz)***	24154.41	***Temperature (degree C)***	27.000				

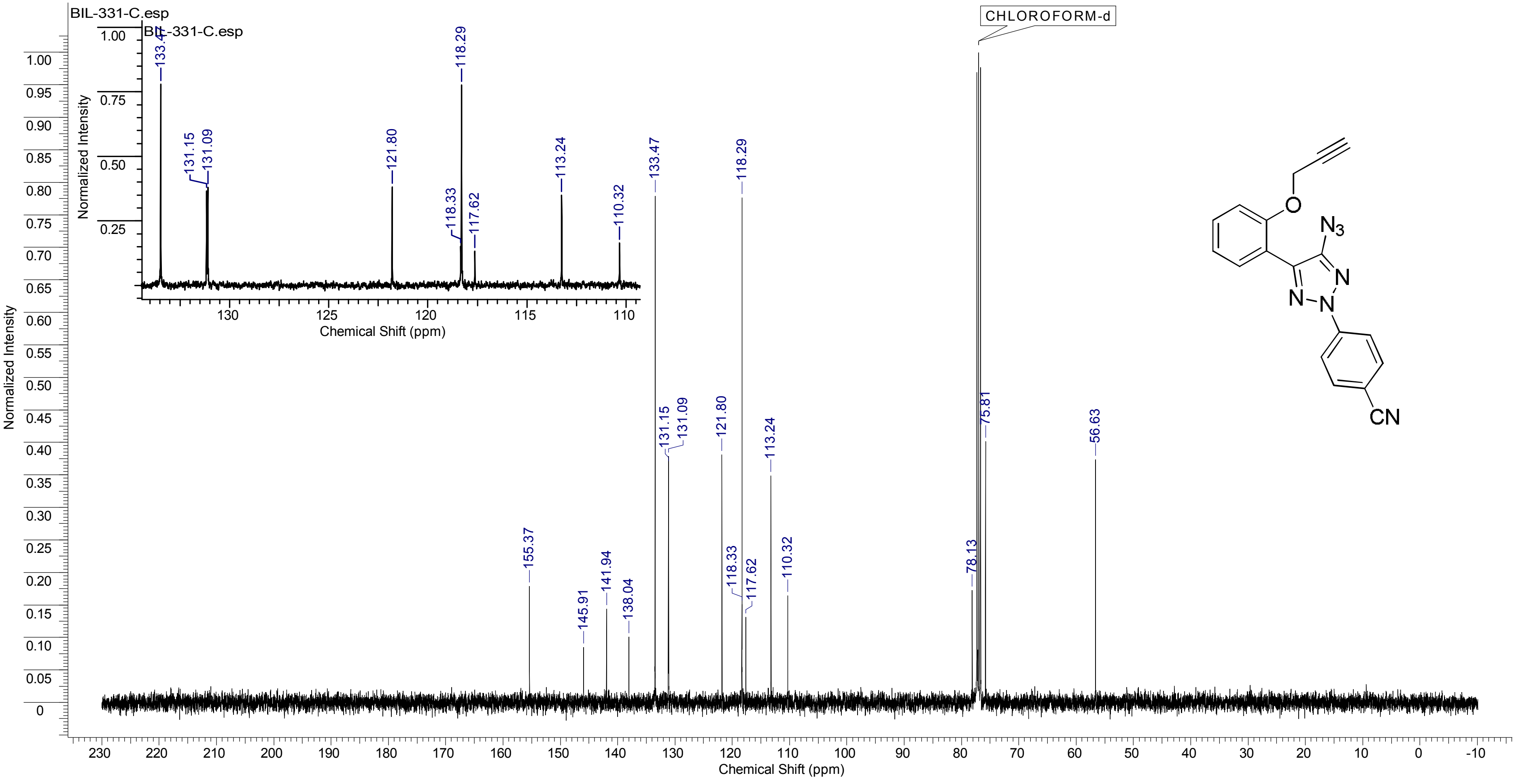

^{13}C NMR spectrum of **3o** (100.6 MHz, $CDCl_3$)

15.05.2019 16:35:04

**Acquisition Time (sec)**	2.5559	**Comment**	5 mm BBO BB-1H/D Z3918/0123			**Date**	25 Oct 2018 09:31:44
**Date Stamp**	25 Oct 2018 09:31:44						
**File Name**						**Frequency (MHz)**	400.13
**Nucleus**	1H	**Number of Transients**	4	**Origin**	spect	**Original Points Count**	16384
**Owner**	root	**Points Count**	65536	**Pulse Sequence**	zg30	**Receiver Gain**	90.50
**SW(cyclical) (Hz)**	6410.26	**Solvent**	CHLOROFORM-d			**Spectrum Offset (Hz)**	2595.9934
**Sweep Width (Hz)**	6410.16	**Temperature (degree C)**	27.000				

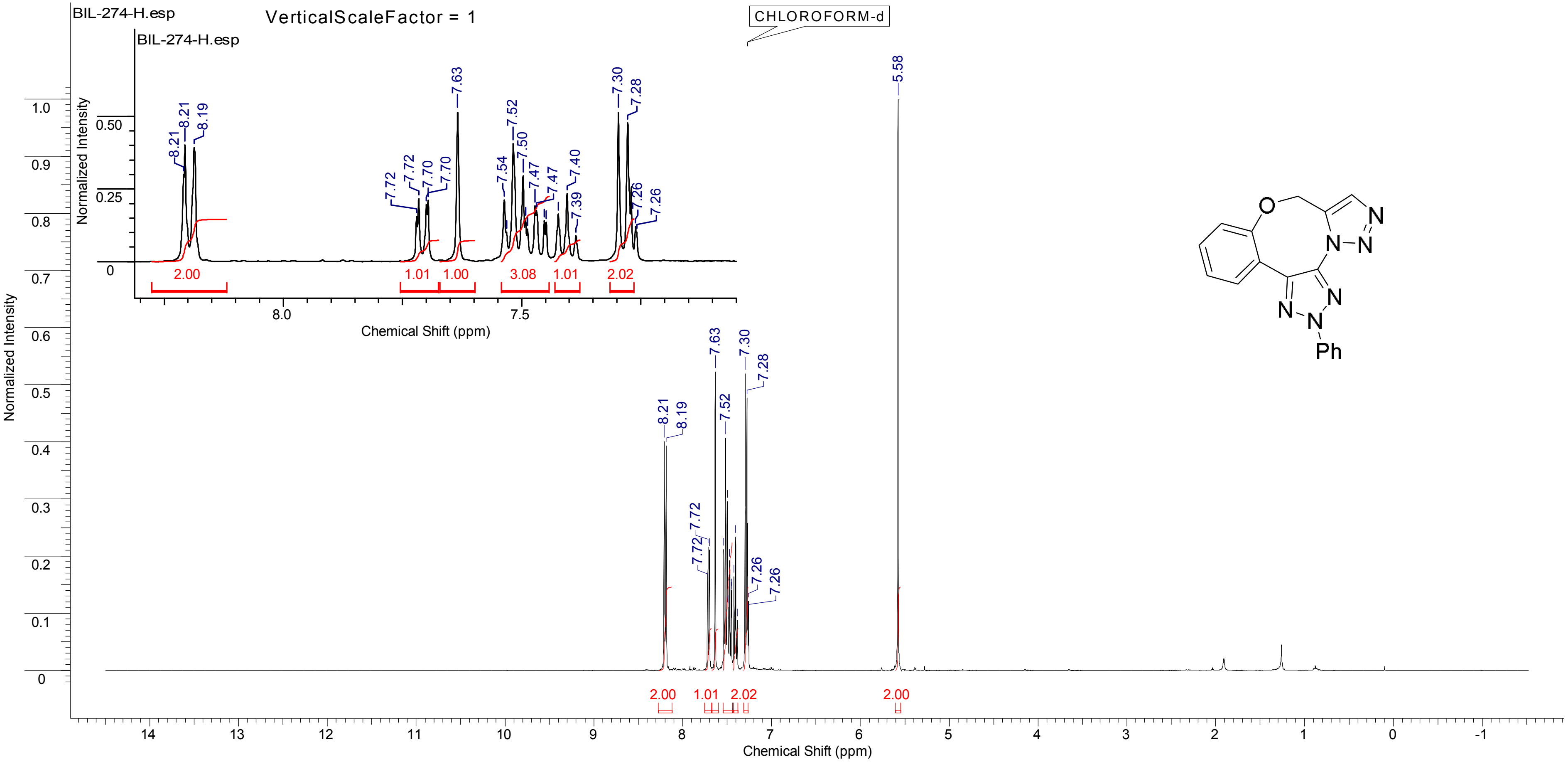

^{1}H NMR spectrum of **4a** (400.1 MHz, $CDCl_3$)

15.05.2019 16:35:59

Acquisition Time (sec)	0.4999	Comment	5 mm BBO BB-1H/D Z3918/0123			Date	25 Oct 2018 09:33:52
Date Stamp	25 Oct 2018 09:33:52						
File Name						Frequency (MHz)	100.61
Nucleus	13C	Number of Transients	127	Origin	spect	Original Points Count	12076
Owner	root	Points Count	65536	Pulse Sequence	zgpg30	Receiver Gain	8192.00
SW(cyclical) (Hz)	24154.59	Solvent	CHLOROFORM-d	Spectrum Offset (Hz)	11052.3359	Sweep Width (Hz)	24154.22
Temperature (degree C)	27.000						

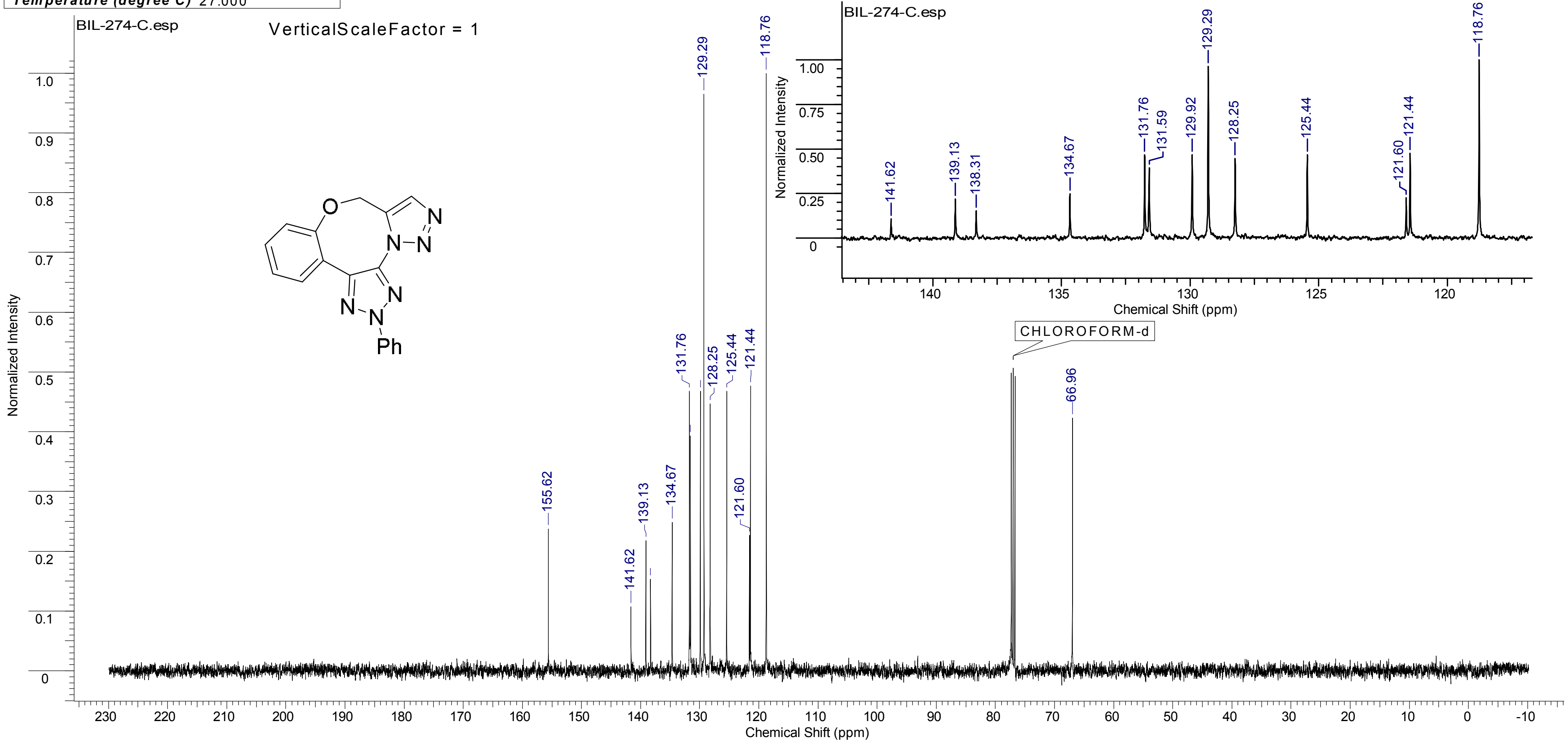

^{13}C NMR spectrum of **4a** (100.6 MHz, $CDCl_3$)

18.05.2019 12:24:45

Acquisition Time (sec)	2.5559	Comment	5 mm BBO BB-1H/D Z3918/0123			Date	12 Nov 2018 12:41:36
Date Stamp	12 Nov 2018 12:41:36						
File Name						Frequency (MHz)	400.13
Nucleus	1H	Number of Transients	4	Origin	spect	Original Points Count	16384
Owner	root	Points Count	65536	Pulse Sequence	zg30	Receiver Gain	161.30
SW(cyclical) (Hz)	6410.26	Solvent	CHLOROFORM-d			Spectrum Offset (Hz)	2595.8953
Sweep Width (Hz)	6410.16	Temperature (degree C)	27.000				

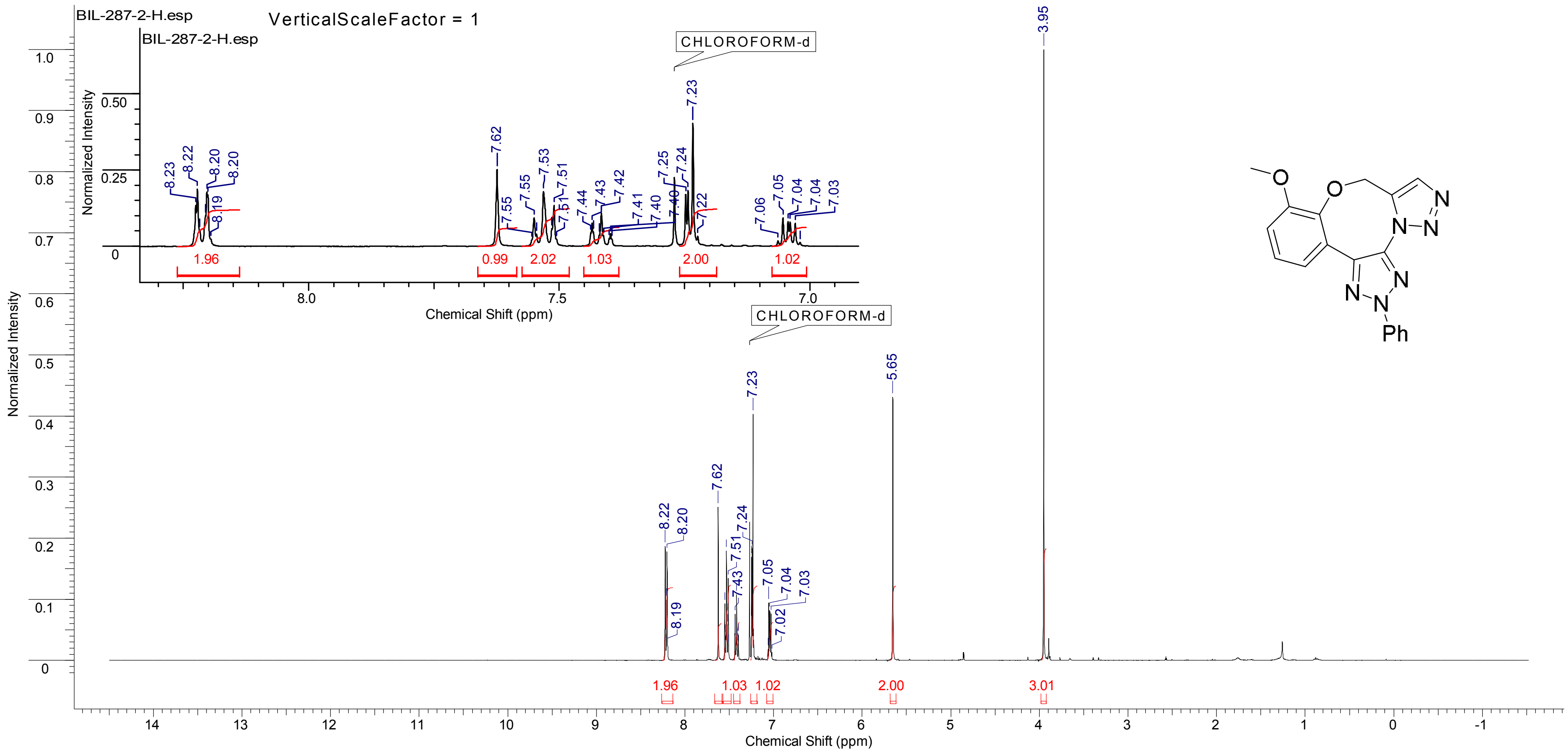

^{1}H NMR spectrum of **4b** (400.1 MHz, $CDCl_3$)

18.05.2019 12:37:50

Acquisition Time (sec)	0.4999	***Comment***	5 mm BBO BB-1H/D Z3918/0123			***Date***	12 Nov 2018 12:43:44
Date Stamp	12 Nov 2018 12:43:44						
File Name						***Frequency (MHz)***	100.61
Nucleus	13C	***Number of Transients***	165	***Origin***	spect	***Original Points Count***	12076
Owner	root	***Points Count***	65536	***Pulse Sequence***	zgpg30	***Receiver Gain***	8192.00
SW(cyclical) (Hz)	24154.59	***Solvent***	CHLOROFORM-d	***Spectrum Offset (Hz)***	11058.9697	***Sweep Width (Hz)***	24154.22
Temperature (degree C)	27.000						

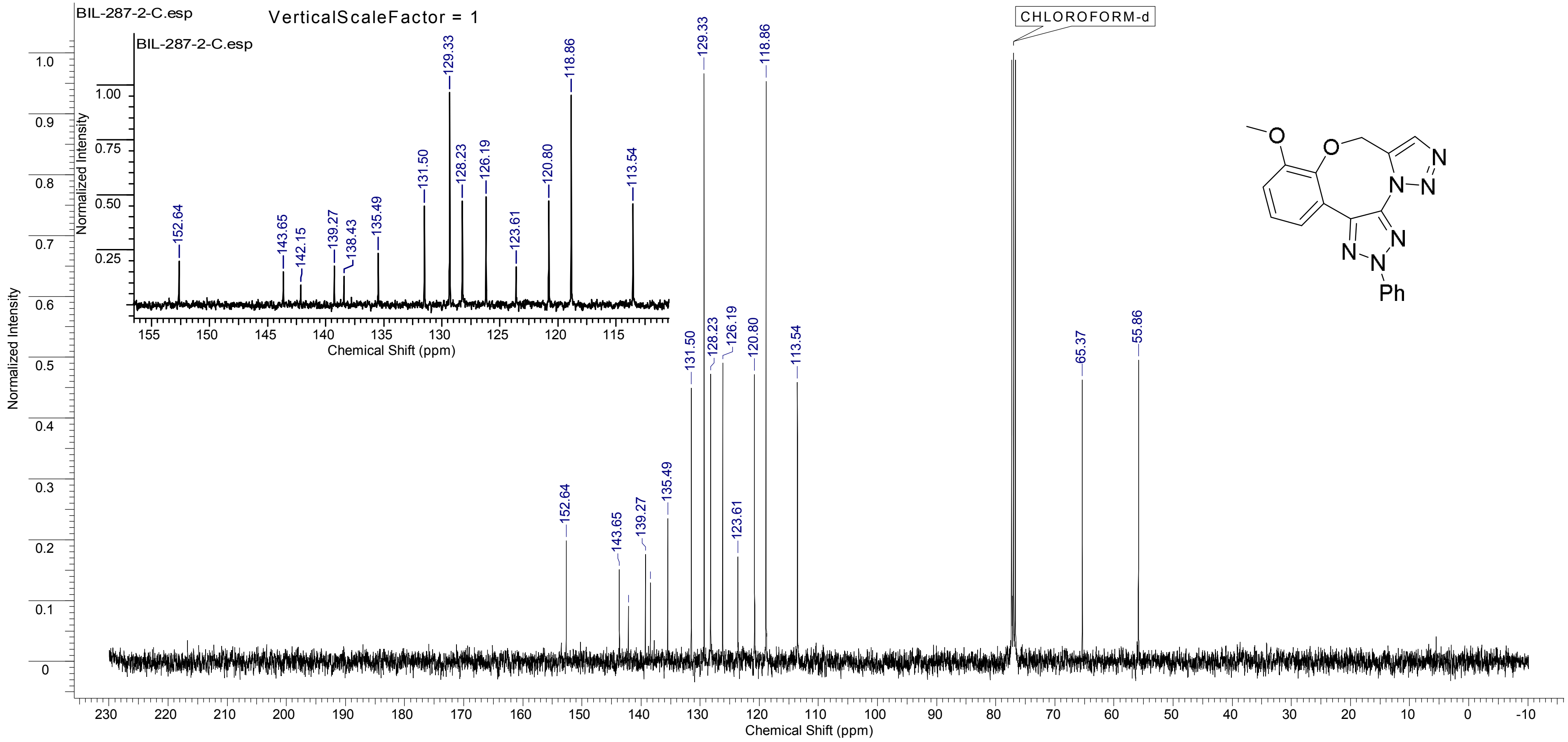

^{13}C NMR spectrum of **4b** (100.6 MHz, $CDCl_3$)

20.11.2019 12:14:02

Acquisition Time (sec)	4.0894	***Comment***	5 mm BBO BB-1H/D Z3918/0123			***Date***	21 May 2019 12:39:28
Date Stamp	21 May 2019 12:39:28						
File Name							
Frequency (MHz)	400.13	***Nucleus***	1H	***Number of Transients***	4	***Origin***	spect
Original Points Count	32768	***Owner***	root	***Points Count***	131072	***Pulse Sequence***	zg30
Receiver Gain	287.40	***SW(cyclical) (Hz)***	8012.82	***Solvent***	DMSO-d6	***Spectrum Offset (Hz)***	2399.2002
Sweep Width (Hz)	8012.76	***Temperature (degree C)***	27.000				

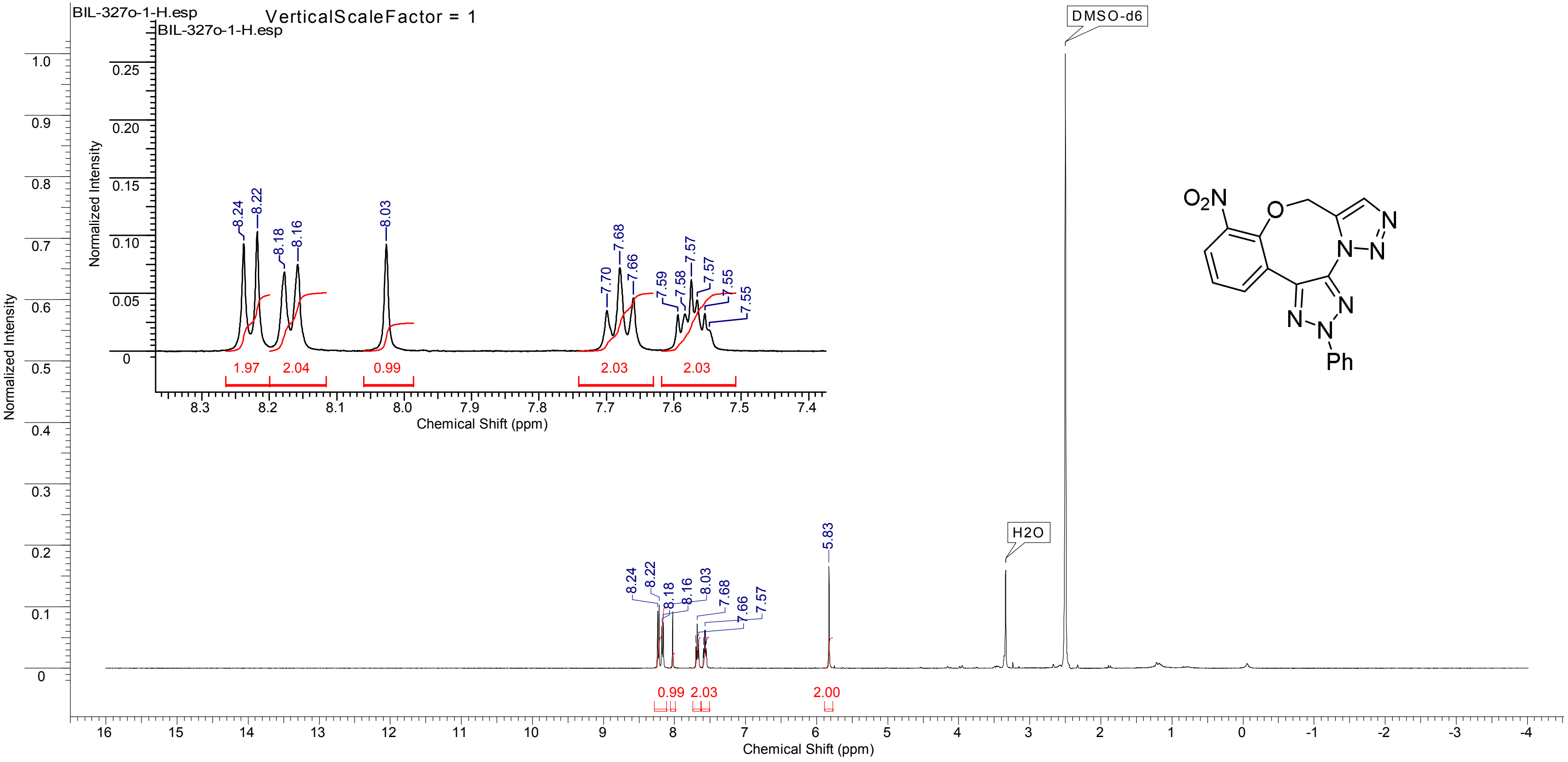

^{1}H NMR spectrum of **4c** (400.1 MHz, $CDCl_3$)

20.11.2019 12:15:34

**Acquisition Time (sec)**	0.6783	**Comment**	5 mm BBO BB-1H/D Z3918/0123			**Date**	21 May 2019 12:41:36
**Date Stamp**	21 May 2019 12:41:36						
**File Name**						**Frequency (MHz)**	100.61
**Nucleus**	13C	**Number of Transients**	538	**Origin**	spect	**Original Points Count**	16384
**Owner**	root	**Points Count**	131072	**Pulse Sequence**	zgpg30	**Receiver Gain**	13004.00
**SW(cyclical) (Hz)**	24154.59	**Solvent**	DMSO-d6	**Spectrum Offset (Hz)**	11025.6758	**Sweep Width (Hz)**	24154.41
**Temperature (degree C)**	27.000						

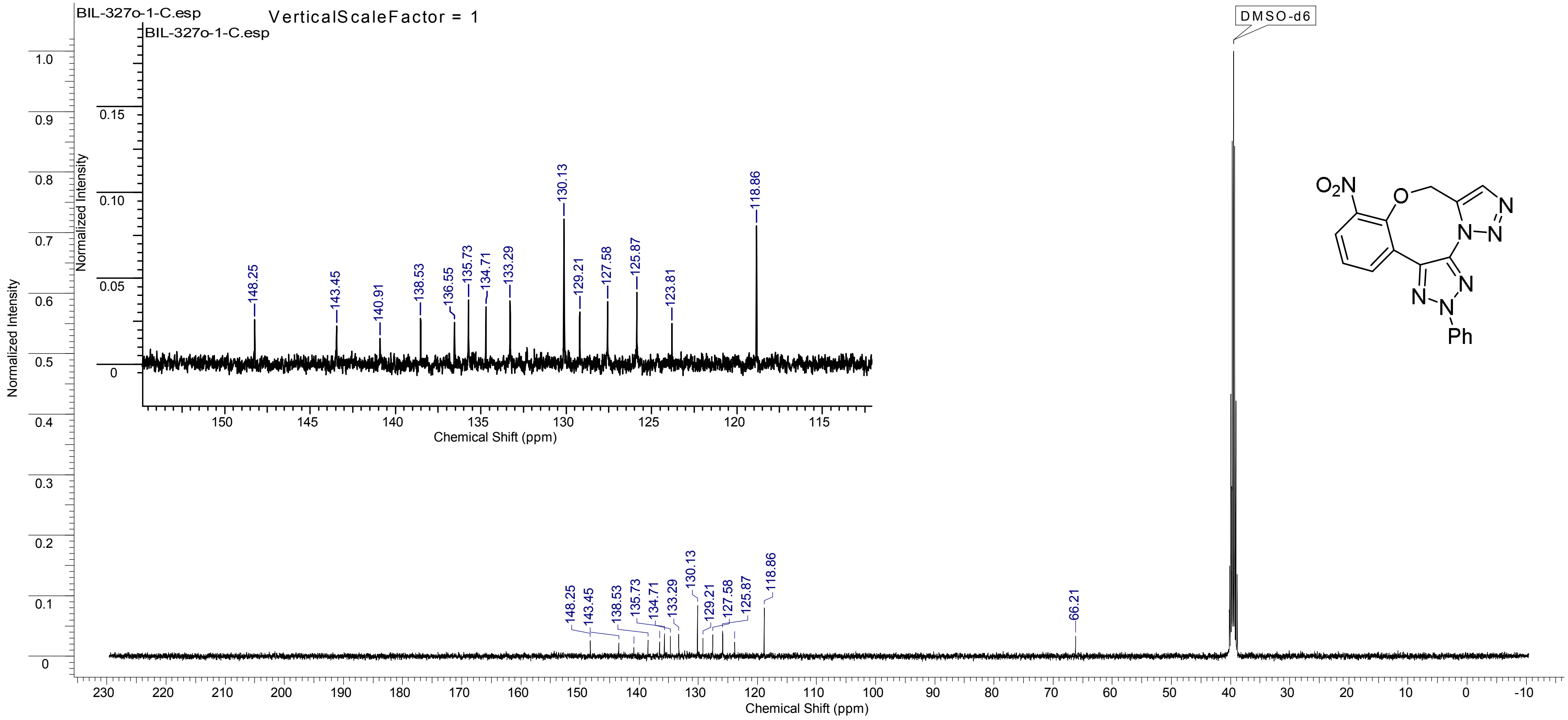

^{13}C NMR spectrum of **4c** (100.6 MHz, $CDCl_3$)

04.12.2019 1:04:03

Acquisition Time (sec)	4.0894	***Comment***	5 mm QNP 1H/13C/31P/19F Z3182/0165			***Date***	26 Nov 2019 10:50:40
Date Stamp	26 Nov 2019 10:50:40						
File Name						***Frequency (MHz)***	400.13
Nucleus	1H	***Number of Transients***	4	***Origin***	spect	***Original Points Count***	32768
Owner	root	***Points Count***	131072	***Pulse Sequence***	zg30	***Receiver Gain***	1290.20
SW(cyclical) (Hz)	8012.82	***Solvent***	CHLOROFORM-d	***Spectrum Offset (Hz)***	3595.9880	***Sweep Width (Hz)***	8012.76
Temperature (degree C)	27.000						

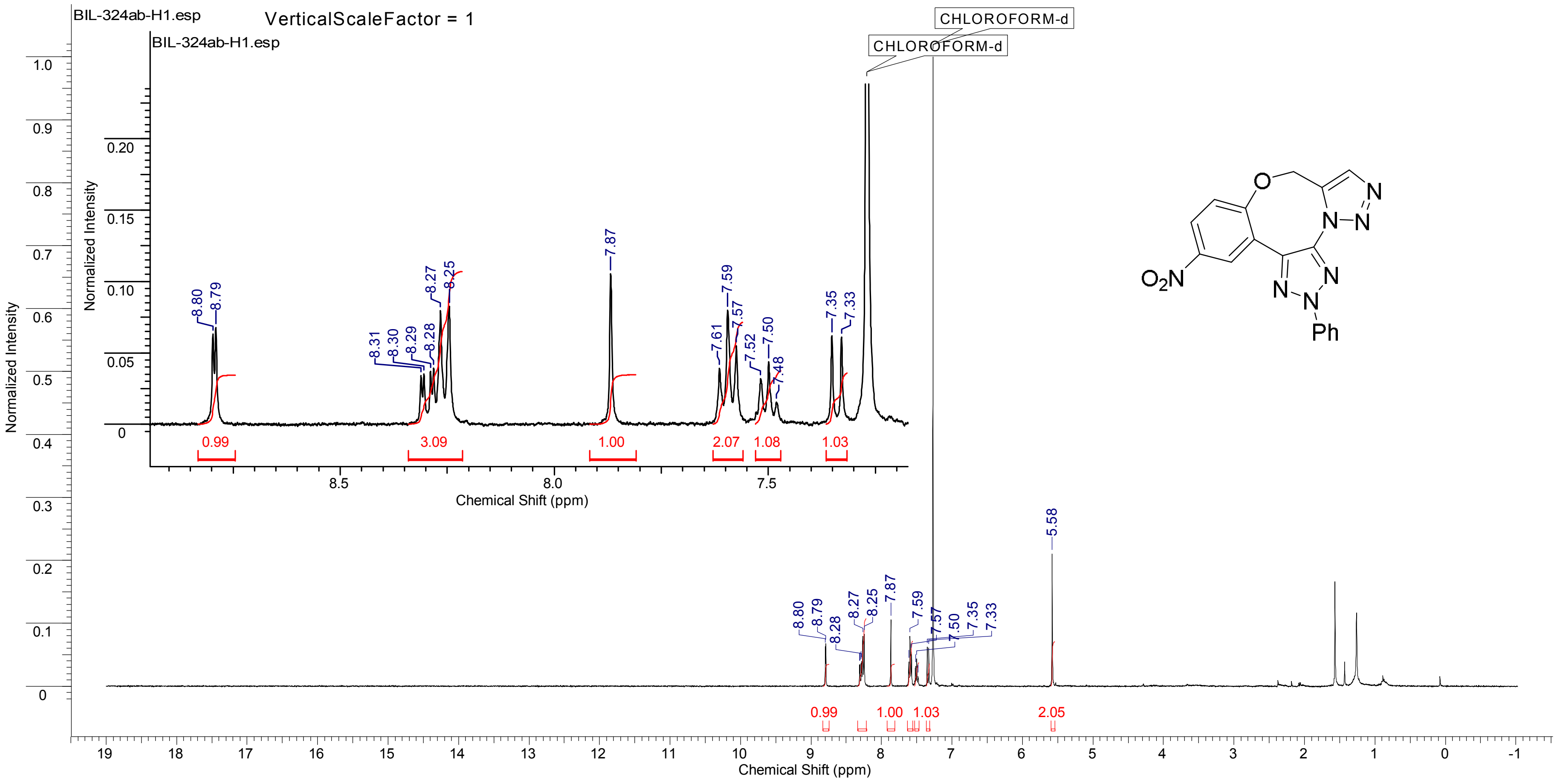

^{1}H NMR spectrum of **4d** (400.1 MHz, $CDCl_3$)

Acquisition Time (sec)	0.6783	***Comment***	Imported from UXNMR.			***Date***	20 Feb 2019 17:49:58
File Name						***Frequency (MHz)***	100.61
Nucleus	13C	***Number of Transients***	217	***Original Points Count***	16384	***Points Count***	131072
Pulse Sequence	zgpg30	***Solvent***	Chloroform-d	***Spectrum Offset (Hz)***	11063.8789	***Sweep Width (Hz)***	24154.59
Temperature (degree C)	27.000						

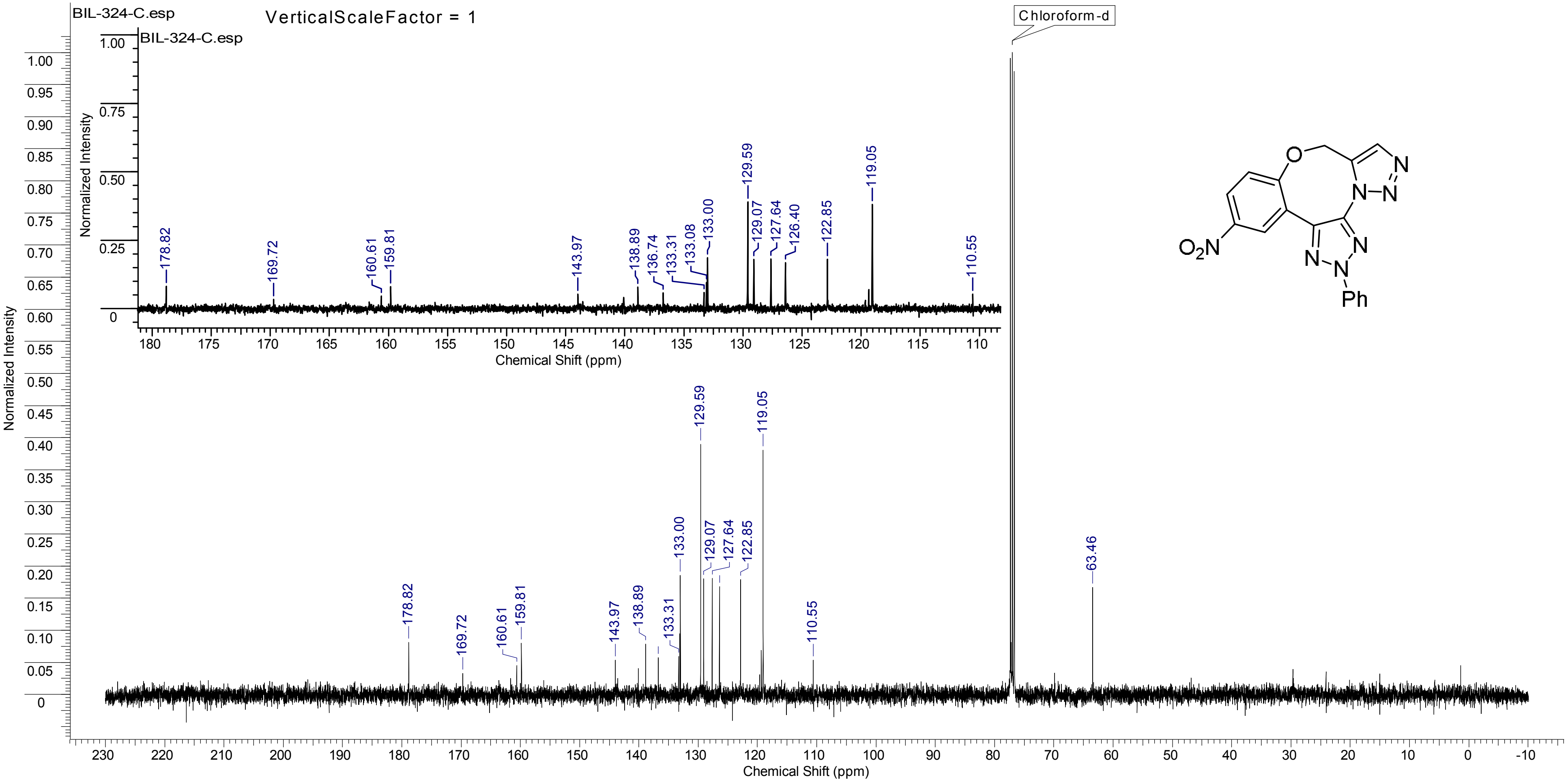

^{13}C NMR spectrum of **4d** (100.6 MHz, $CDCl_3$)

18.05.2019 13:59:21

Acquisition Time (sec)	4.0894	***Comment***	5 mm BBO BB-1H/D Z3918/0123			***Date***	12 Feb 2019 11:44:00
Date Stamp	12 Feb 2019 11:44:00						
File Name						***Frequency (MHz)***	400.13
Nucleus	1H	***Number of Transients***	4	***Origin***	spect	***Original Points Count***	32768
Owner	root	***Points Count***	131072	***Pulse Sequence***	zg30	***Receiver Gain***	203.20
SW(cyclical) (Hz)	8012.82	***Solvent***	CHLOROFORM-d			***Spectrum Offset (Hz)***	2395.8254
Sweep Width (Hz)	8012.76	***Temperature (degree C)***	27.000				

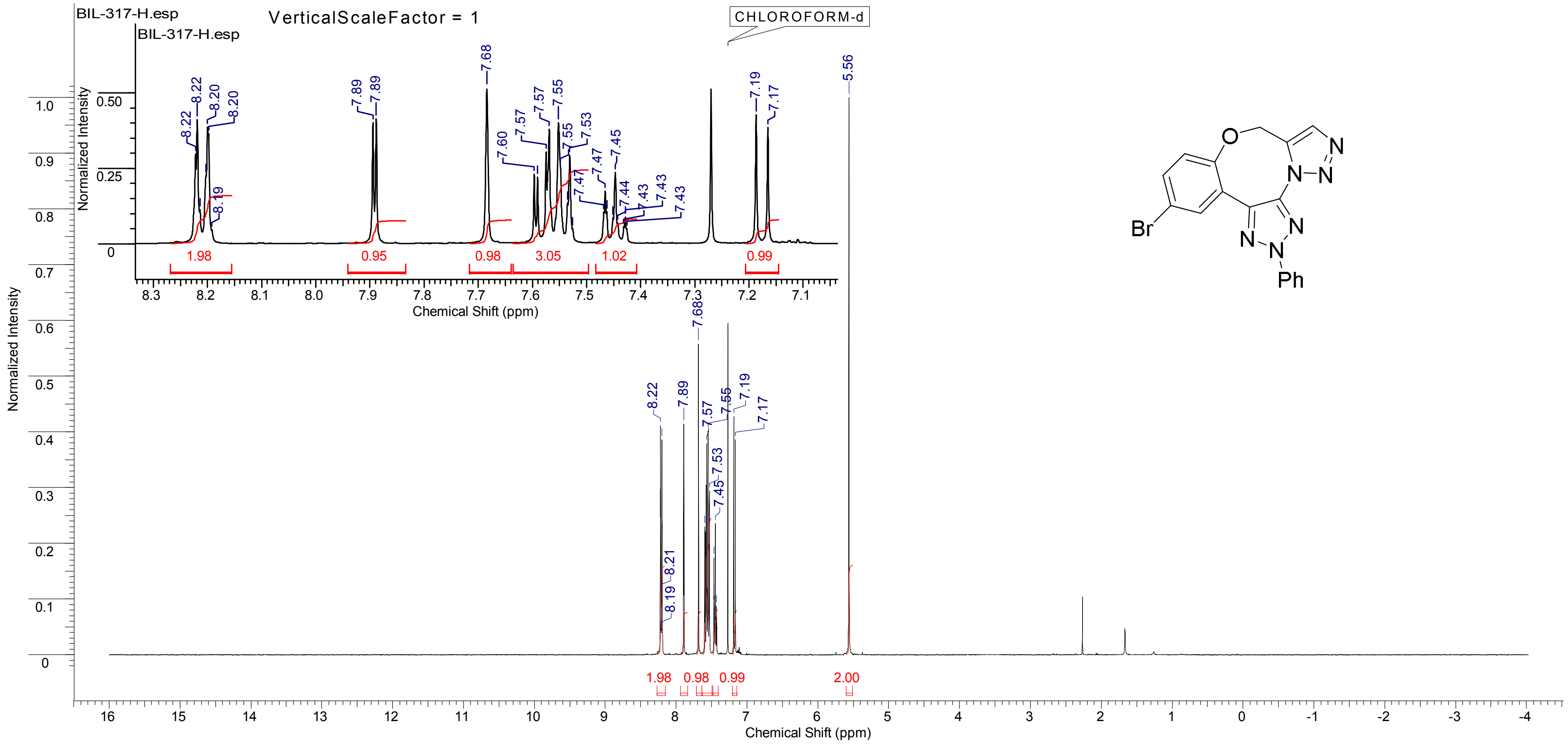

^{1}H NMR spectrum of **4e** (400.1 MHz, $CDCl_3$)

18.05.2019 14:00:38

**Acquisition Time (sec)**	0.6783	**Comment**	5 mm BBO BB-1H/D Z3918/0123			**Date**	12 Feb 2019 11:46:08
**Date Stamp**	12 Feb 2019 11:46:08						
**File Name**						**Frequency (MHz)**	100.61
**Nucleus**	13C	**Number of Transients**	123	**Origin**	spect	**Original Points Count**	16384
**Owner**	root	**Points Count**	131072	**Pulse Sequence**	zgpg30	**Receiver Gain**	14596.50
**SW(cyclical) (Hz)**	24154.59	**Solvent**	CHLOROFORM-d	**Spectrum Offset (Hz)**	11061.0898	**Sweep Width (Hz)**	24154.41
**Temperature (degree C)**	27.000						

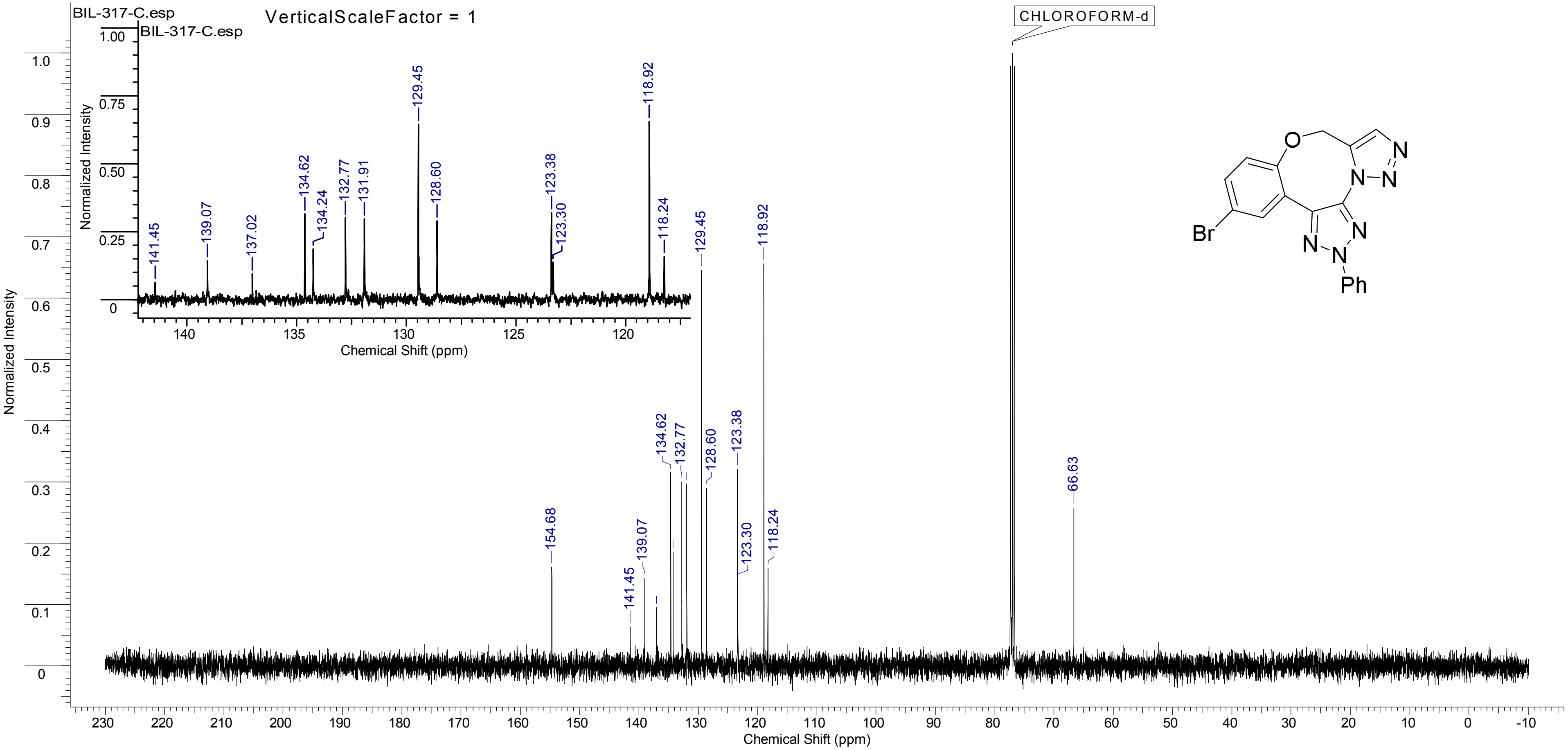

^{13}C NMR spectrum of **4e** (100.6 MHz, $CDCl_3$)

21.06.2019 18:44:51

Acquisition Time (sec)	4.0894	Comment	Imported from UXNMR.		Date	21 Jun 2019 15:46:48		
File Name			Frequency (MHz)	400.13	Nucleus	1H	Number of Transients	4
Original Points Count	32768	Points Count	131072	Pulse Sequence	zg30	Solvent	CHLOROFORM-d	
Spectrum Offset (Hz)	2395.8828	Sweep Width (Hz)	8012.82	Temperature (degree C)	27.000			

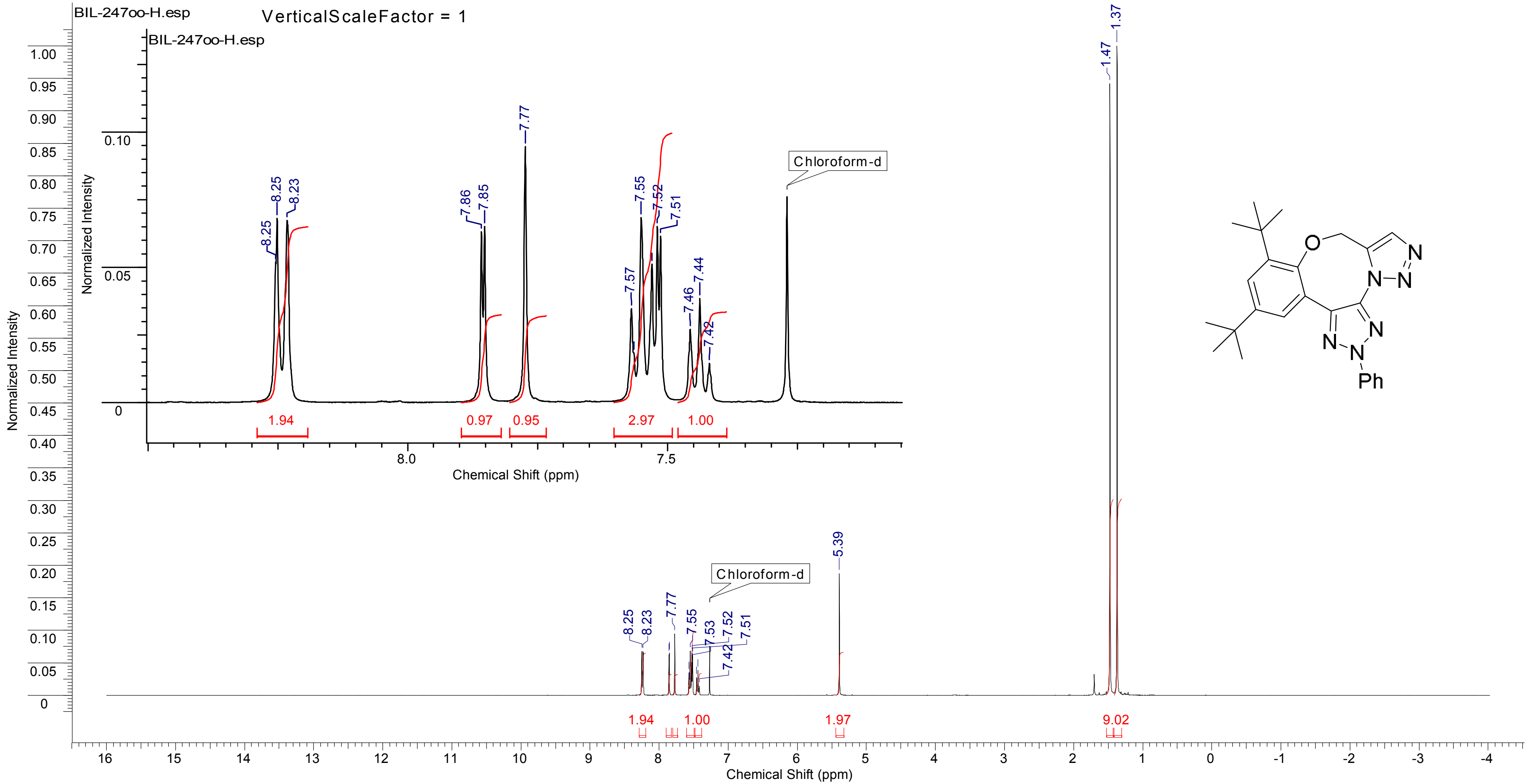

^{1}H NMR spectrum of **4f** (400.1 MHz, $CDCl_3$)

21.06.2019 18:48:01

Acquisition Time (sec)	0.6783	Comment	Imported from UXNMR.			Date	21 Jun 2019 15:54:22		
File Name				Frequency (MHz)	100.61	Nucleus	13C	Number of Transients	185
Original Points Count	16384	Points Count	131072	Pulse Sequence	zgpg30	Solvent	Chloroform-d	Spectrum Offset (Hz)	11062.0352
Sweep Width (Hz)	24154.59	Temperature (degree C)	27.000						

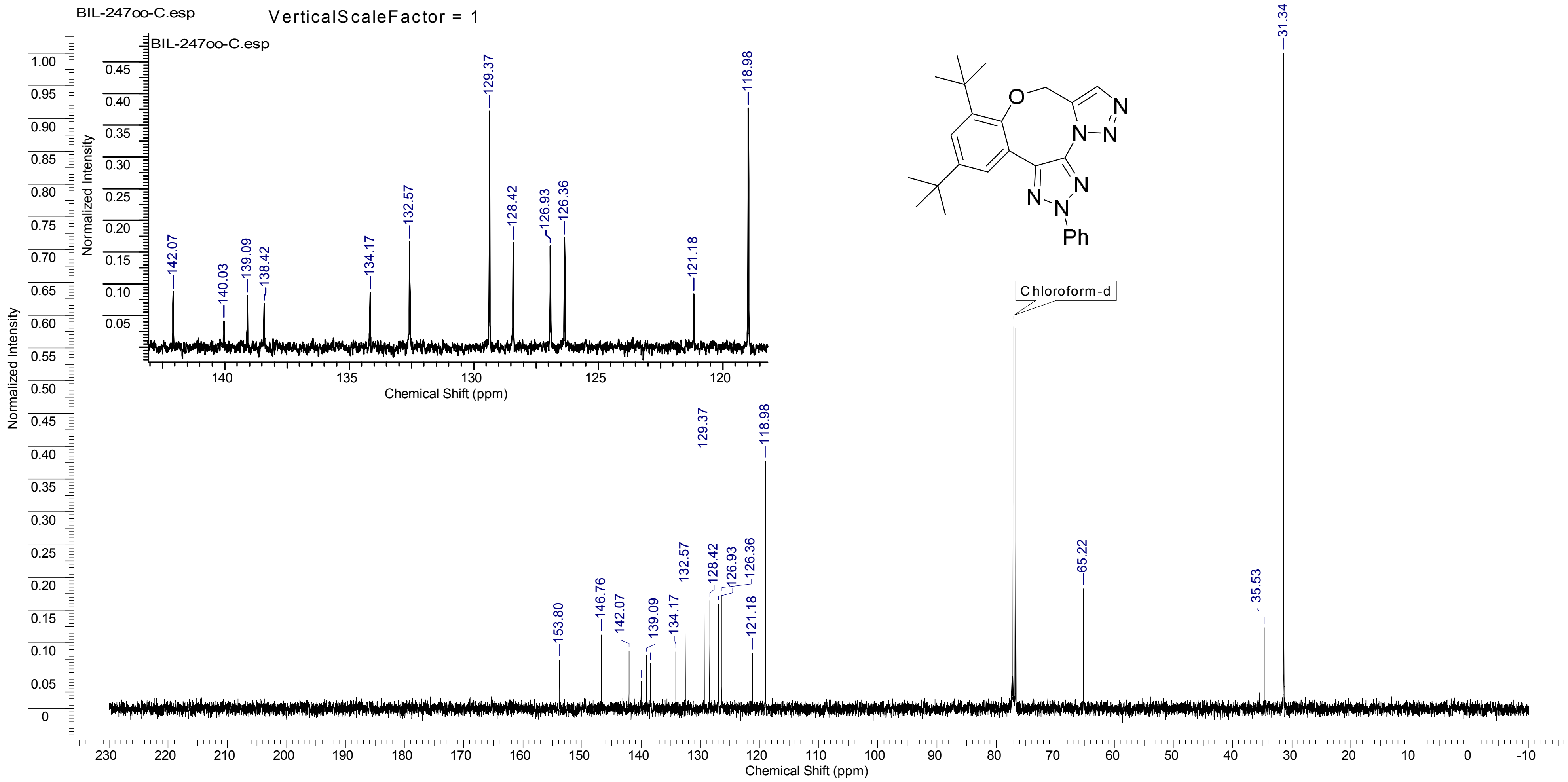

^{13}C NMR spectrum of **4f** (100.6 MHz, $CDCl_3$)

20.05.2019 14:38:49

**Acquisition Time (sec)**	4.0894	**Comment**	5 mm BBO BB-1H/D Z3918/0123			**Date**	12 Mar 2019 11:24:48
**Date Stamp**	12 Mar 2019 11:24:48						
**File Name**						**Frequency (MHz)**	400.13
**Nucleus**	1H	**Number of Transients**	4	**Origin**	spect	**Original Points Count**	32768
**Owner**	root	**Points Count**	131072	**Pulse Sequence**	zg30	**Receiver Gain**	143.70
**SW(cyclical) (Hz)**	8012.82	**Solvent**	CHLOROFORM-d			**Spectrum Offset (Hz)**	2395.8254
**Sweep Width (Hz)**	8012.76	**Temperature (degree C)**	27.000				

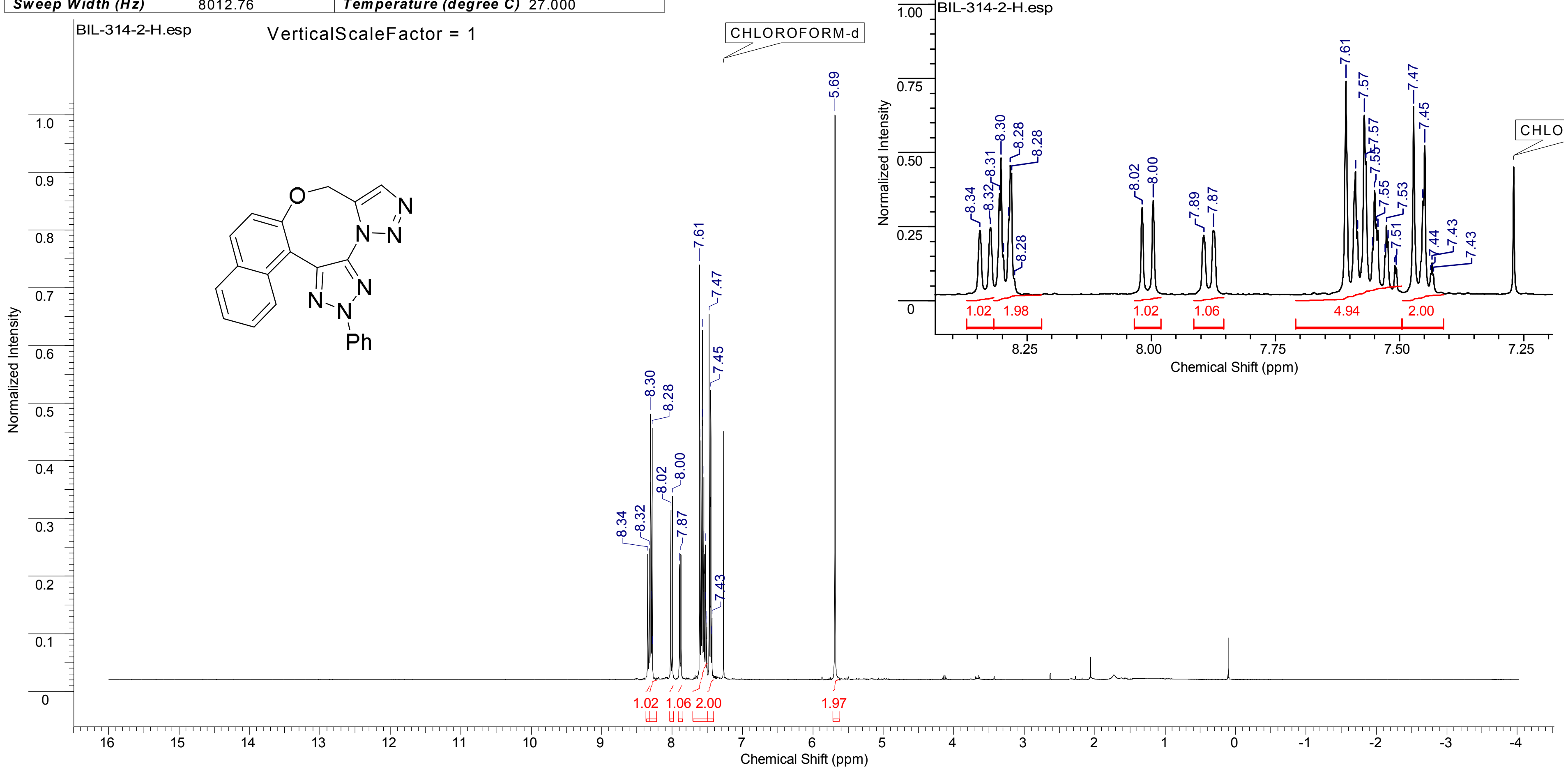

^{1}H NMR spectrum of **4g** (400.1 MHz, $CDCl_3$)

20.05.2019 14:40:40

**Acquisition Time (sec)**	0.6783	**Comment**	5 mm BBO BB-1H/D Z3918/0123			**Date**	12 Mar 2019 11:29:04
**Date Stamp**	12 Mar 2019 11:29:04						
**File Name**							
**Frequency (MHz)**	100.62	**Nucleus**	13C	**Number of Transients**	218	**Origin**	spect
**Original Points Count**	16384	**Owner**	root	**Points Count**	16384	**Pulse Sequence**	zgpg30
**Receiver Gain**	16384.00	**SW(cyclical) (Hz)**	24154.59	**Solvent**	CHLOROFORM-d	**Spectrum Offset (Hz)**	11058.5283
**Sweep Width (Hz)**	24153.11	**Temperature (degree C)**	27.000				

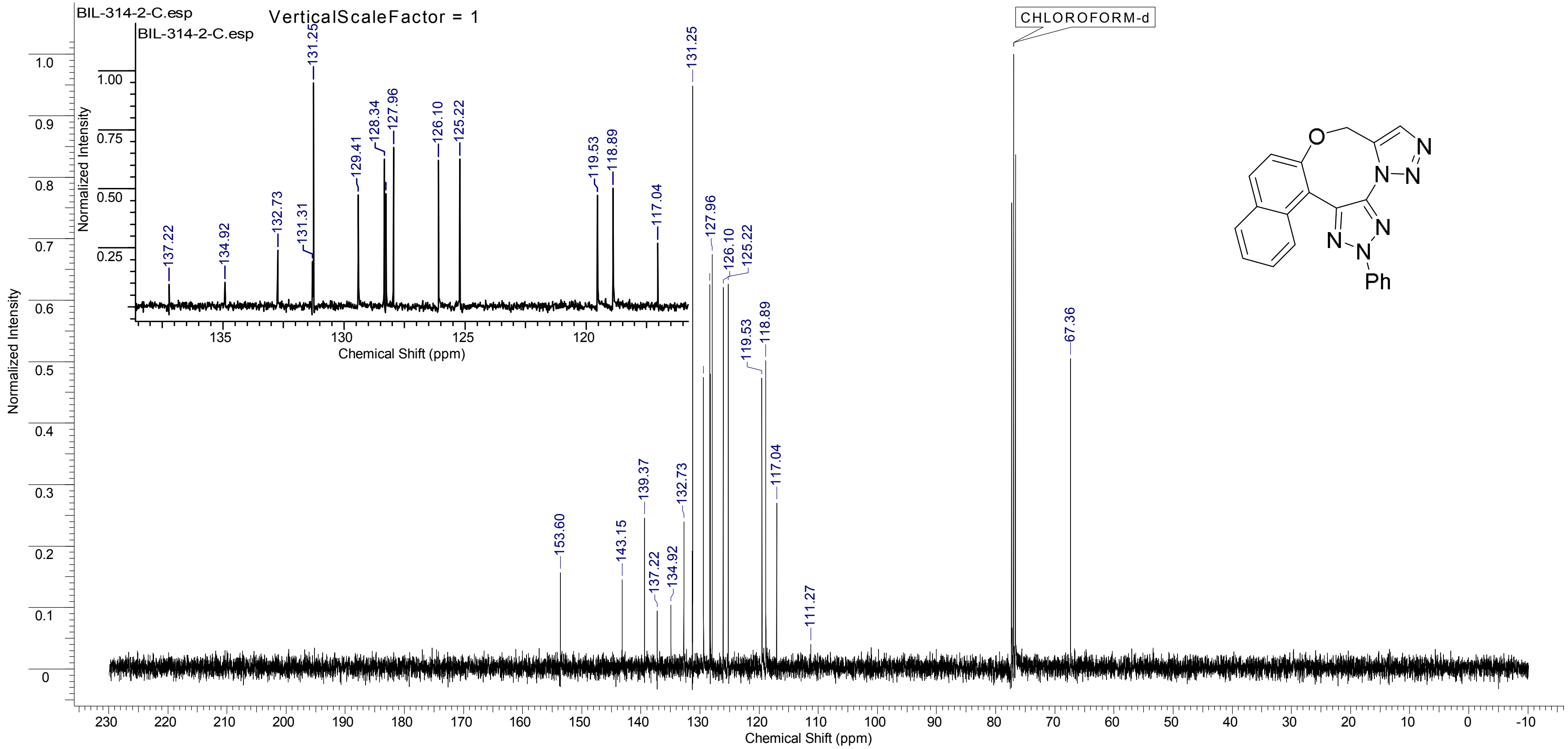

^{13}C NMR spectrum of **4g** (100.6 MHz, $CDCl_3$)

20.05.2019 15:53:16

**Acquisition Time (sec)**	2.5559	**Comment**	5 mm BBO BB-1H/D Z3918/0123			**Date**	01 Nov 2018 08:29:52
**Date Stamp**	01 Nov 2018 08:29:52						
**File Name**						**Frequency (MHz)**	400.13
**Nucleus**	1H	**Number of Transients**	5	**Origin**	spect	**Original Points Count**	16384
**Owner**	root	**Points Count**	65536	**Pulse Sequence**	zg30	**Receiver Gain**	114.00
**SW(cyclical) (Hz)**	6410.26	**Solvent**	CHLOROFORM-d			**Spectrum Offset (Hz)**	2594.6238
**Sweep Width (Hz)**	6410.16	**Temperature (degree C)**	27.000				

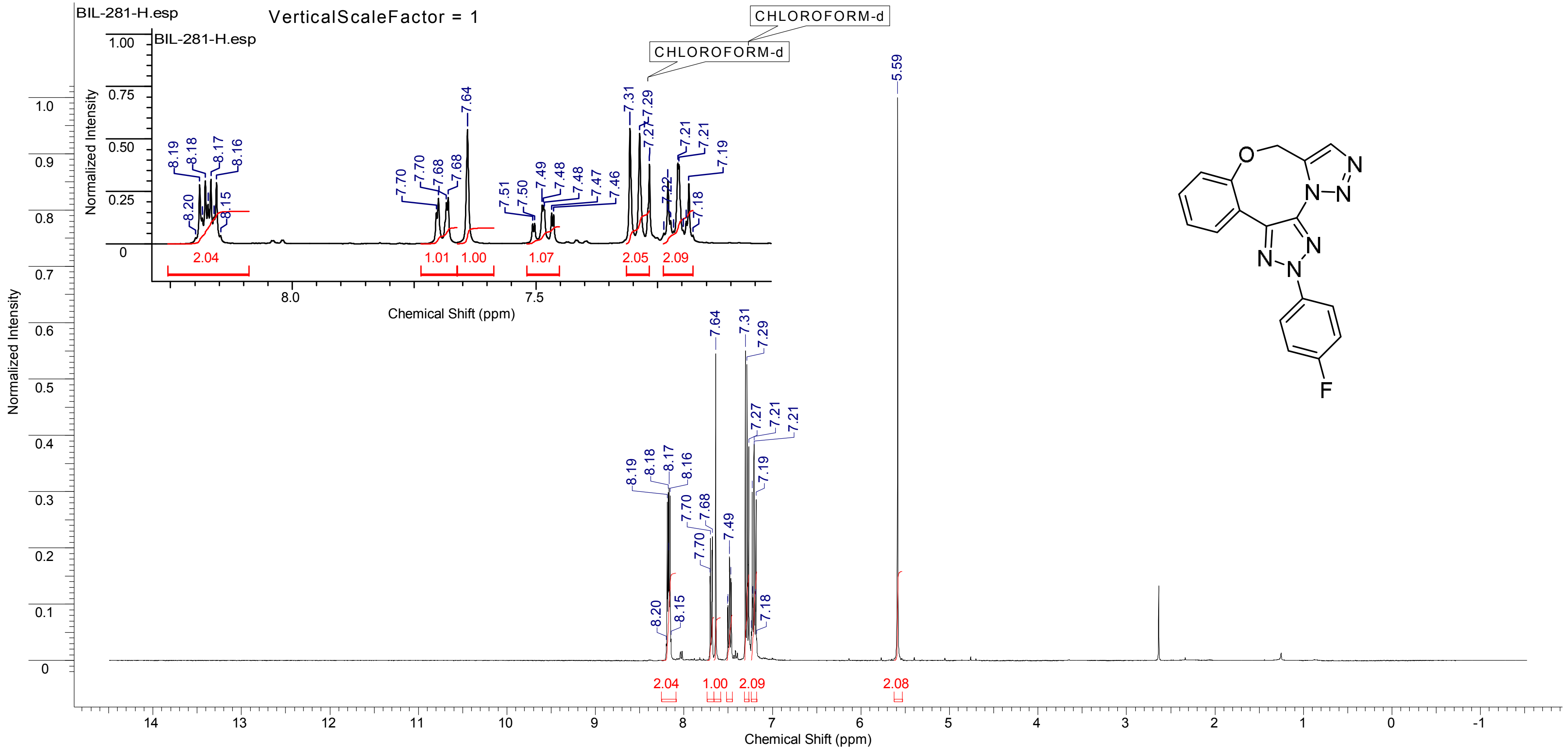

^{1}H NMR spectrum of **4h** (400.1 MHz, $CDCl_3$)

20.05.2019 15:54:08

Acquisition Time (sec)	0.4999	***Comment***	5 mm BBO BB-1H/D Z3918/0123			***Date***	01 Nov 2018 08:32:00
Date Stamp	01 Nov 2018 08:32:00						
File Name						***Frequency (MHz)***	100.61
Nucleus	13C	***Number of Transients***	129	***Origin***	spect	***Original Points Count***	12076
Owner	root	***Points Count***	65536	***Pulse Sequence***	zgpg30	***Receiver Gain***	8192.00
SW(cyclical) (Hz)	24154.59	***Solvent***	CHLOROFORM-d	***Spectrum Offset (Hz)***	11056.7578	***Sweep Width (Hz)***	24154.22
Temperature (degree C)	27.000						

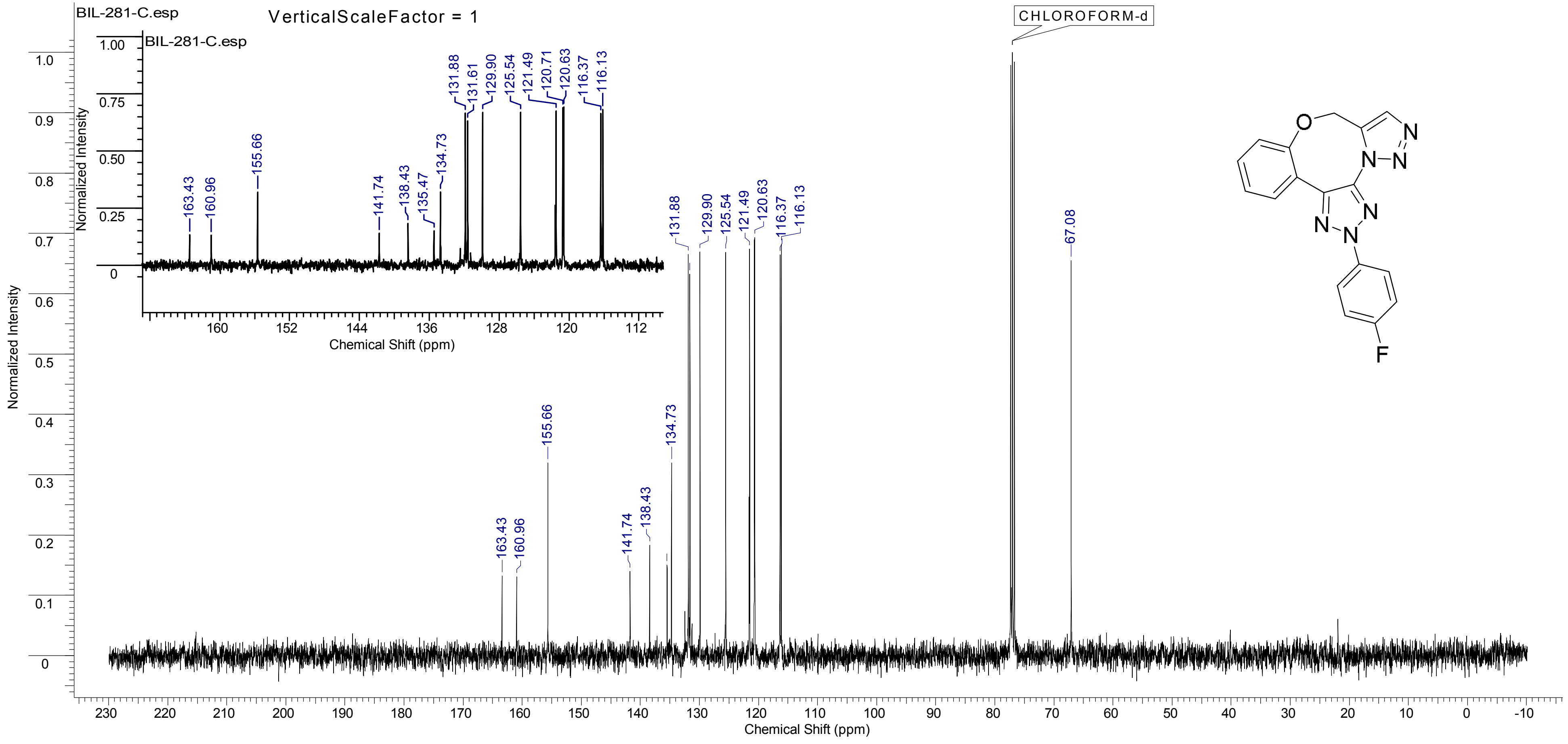

^{13}C NMR spectrum of **4h** (100.6 MHz, $CDCl_3$)

20.05.2019 17:35:26

**Acquisition Time (sec)**	2.5559	**Comment**	5 mm BBO BB-1H/D Z3918/0123			**Date**	17 Dec 2018 12:16:00		
**Date Stamp**	17 Dec 2018 12:16:00			**File Name**				**Frequency (MHz)**	400.13
**Nucleus**	1H	**Number of Transients**	4	**Origin**	spect	**Original Points Count**	16384	**Owner**	root
**Points Count**	65536	**Pulse Sequence**	zg30	**Receiver Gain**	143.70	**SW(cyclical) (Hz)**	6410.26	**Solvent**	CHLOROFORM-d
**Spectrum Offset (Hz)**	2595.9934	**Sweep Width (Hz)**	6410.16	**Temperature (degree C)**	27.000				

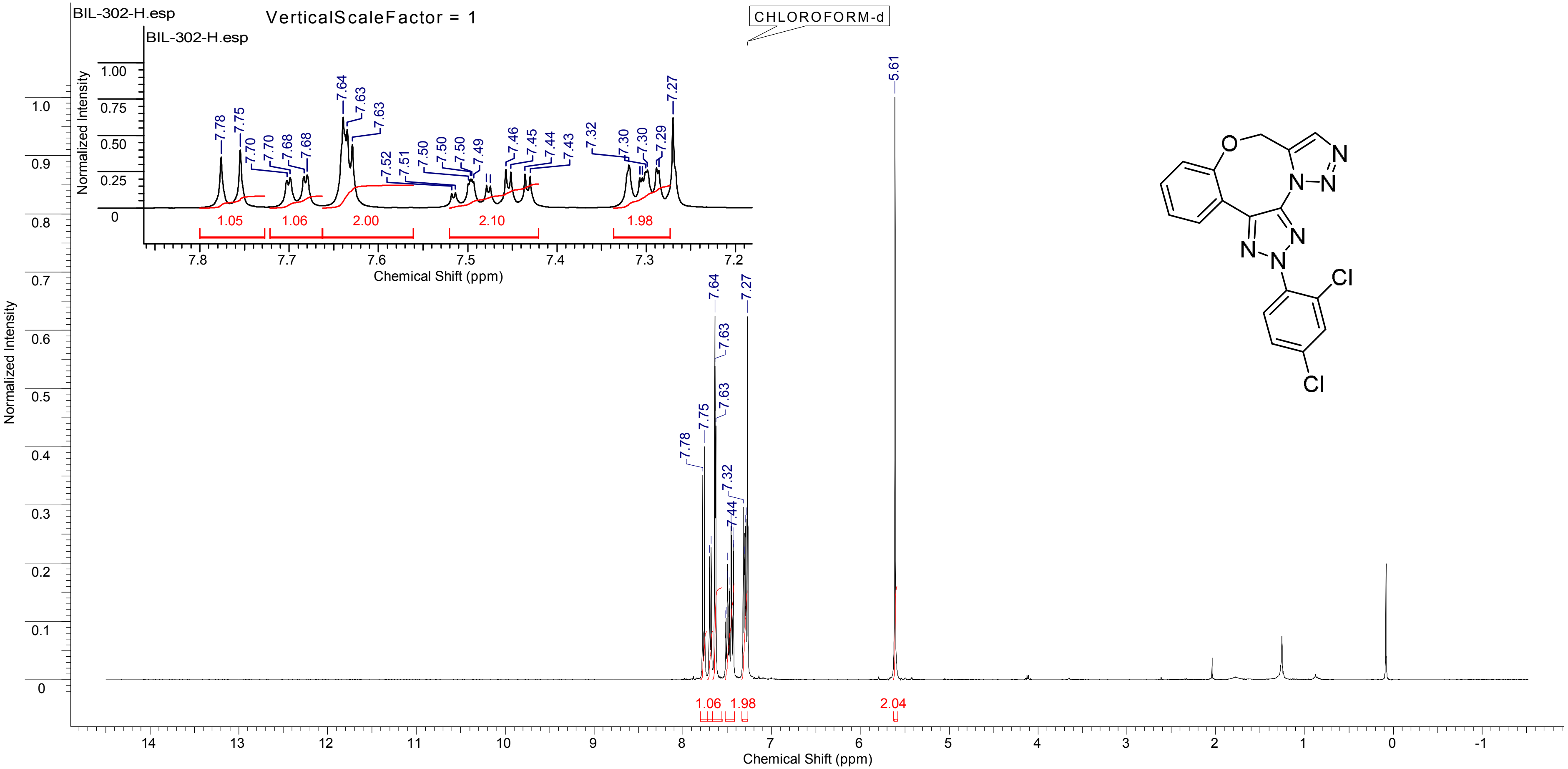

^{1}H NMR spectrum of **4i** (400.1 MHz, $CDCl_3$)

20.05.2019 17:36:20

Acquisition Time (sec)	0.6783	Comment	5 mm BBO BB-1H/D Z3918/0123	Date	17 Dec 2018 12:18:08
Date Stamp	17 Dec 2018 12:18:08		File Name		
Frequency (MHz)	100.61	Nucleus	13C	Number of Transients	43	Origin	spect
Original Points Count	16384	Owner	root	Points Count	131072	Pulse Sequence	zgpg30
Receiver Gain	13004.00	SW(cyclical) (Hz)	24154.59	Solvent	CHLOROFORM-d		
Spectrum Offset (Hz)	11057.2197	Sweep Width (Hz)	24154.41	Temperature (degree C)	27.000		

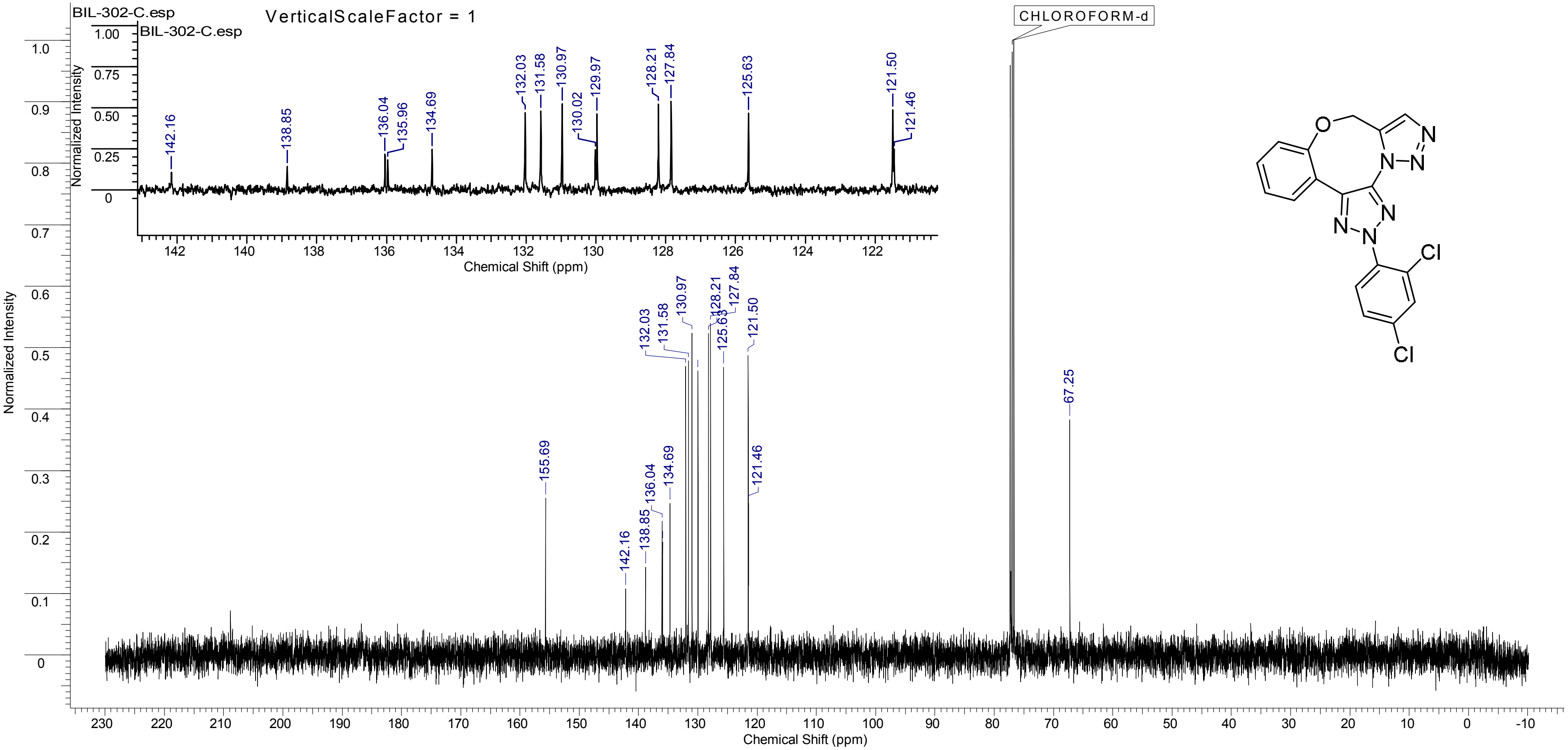

^{13}C NMR spectrum of **4i** (100.6 MHz, $CDCl_3$)

04.12.2019 0:50:15

Acquisition Time (sec)	2.5559	***Comment***	5 mm BBO BB-1H/D Z3918/0123			***Date***	10 Dec 2018 12:50:08
Date Stamp	10 Dec 2018 12:50:08			***File Name***			
Frequency (MHz)	400.13	***Nucleus***	1H	***Number of Transients***	4	***Origin***	spect
Original Points Count	16384	***Owner***	root	***Points Count***	65536	***Pulse Sequence***	zg30
Receiver Gain	228.10	***SW(cyclical) (Hz)***	6410.26	***Solvent***	CHLOROFORM-d		
Spectrum Offset (Hz)	2595.7979	***Sweep Width (Hz)***	6410.16	***Temperature (degree C)***	27.000		

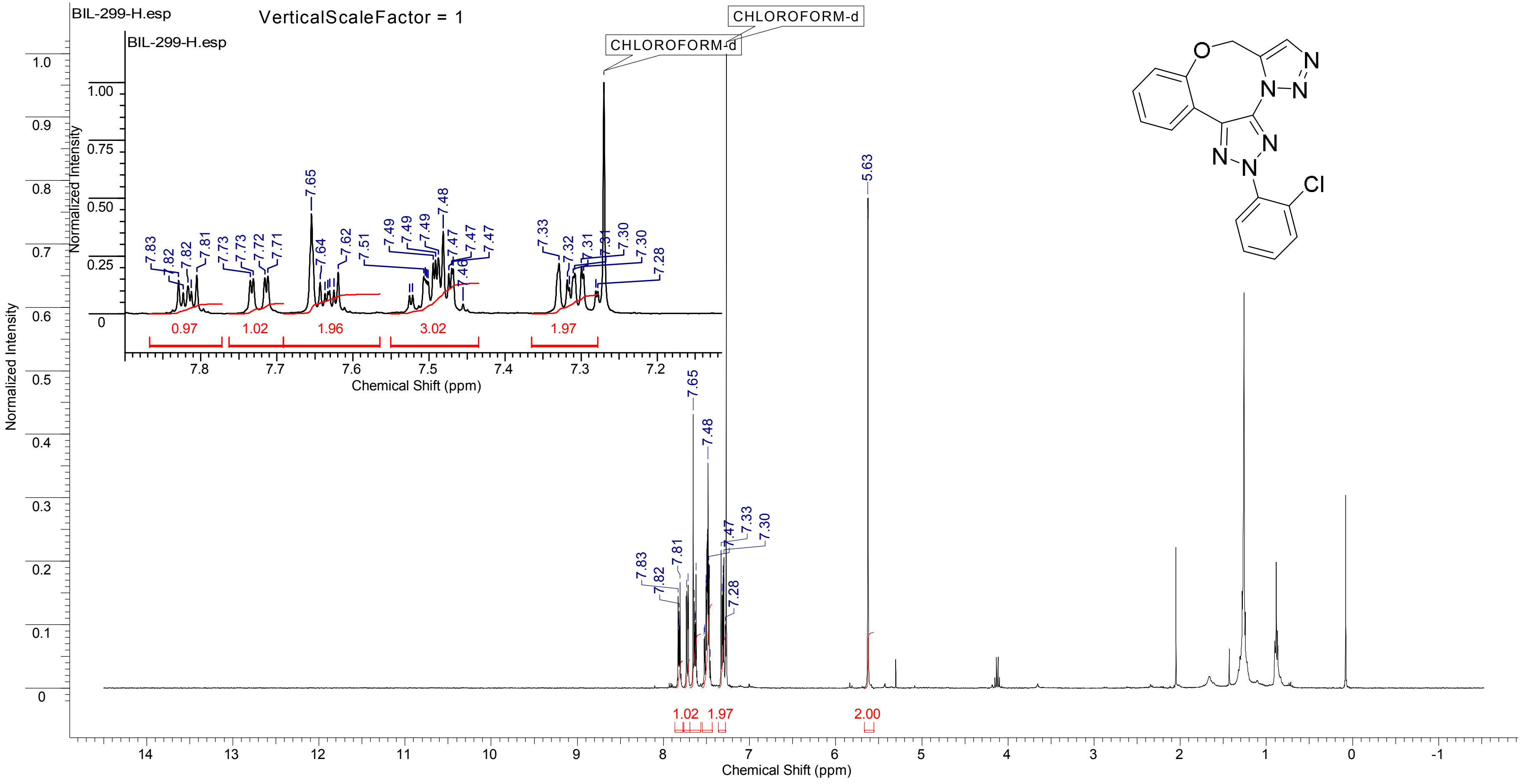

^{1}H NMR spectrum of **4j** (400.1 MHz, $CDCl_3$)

20.05.2019 18:05:41

Acquisition Time (sec)	0.6783	Comment	5 mm BBO BB-1H/D Z3918/0123			Date	10 Dec 2018 12:52:16
Date Stamp	10 Dec 2018 12:52:16			File Name			
Frequency (MHz)	100.61	Nucleus	13C	Number of Transients	194	Origin	spect
Original Points Count	16384	Owner	root	Points Count	131072	Pulse Sequence	zgpg30
Receiver Gain	13004.00	SW(cyclical) (Hz)	24154.59	Solvent	CHLOROFORM-d		
Spectrum Offset (Hz)	11062.0107	Sweep Width (Hz)	24154.41	Temperature (degree C)	27.000		

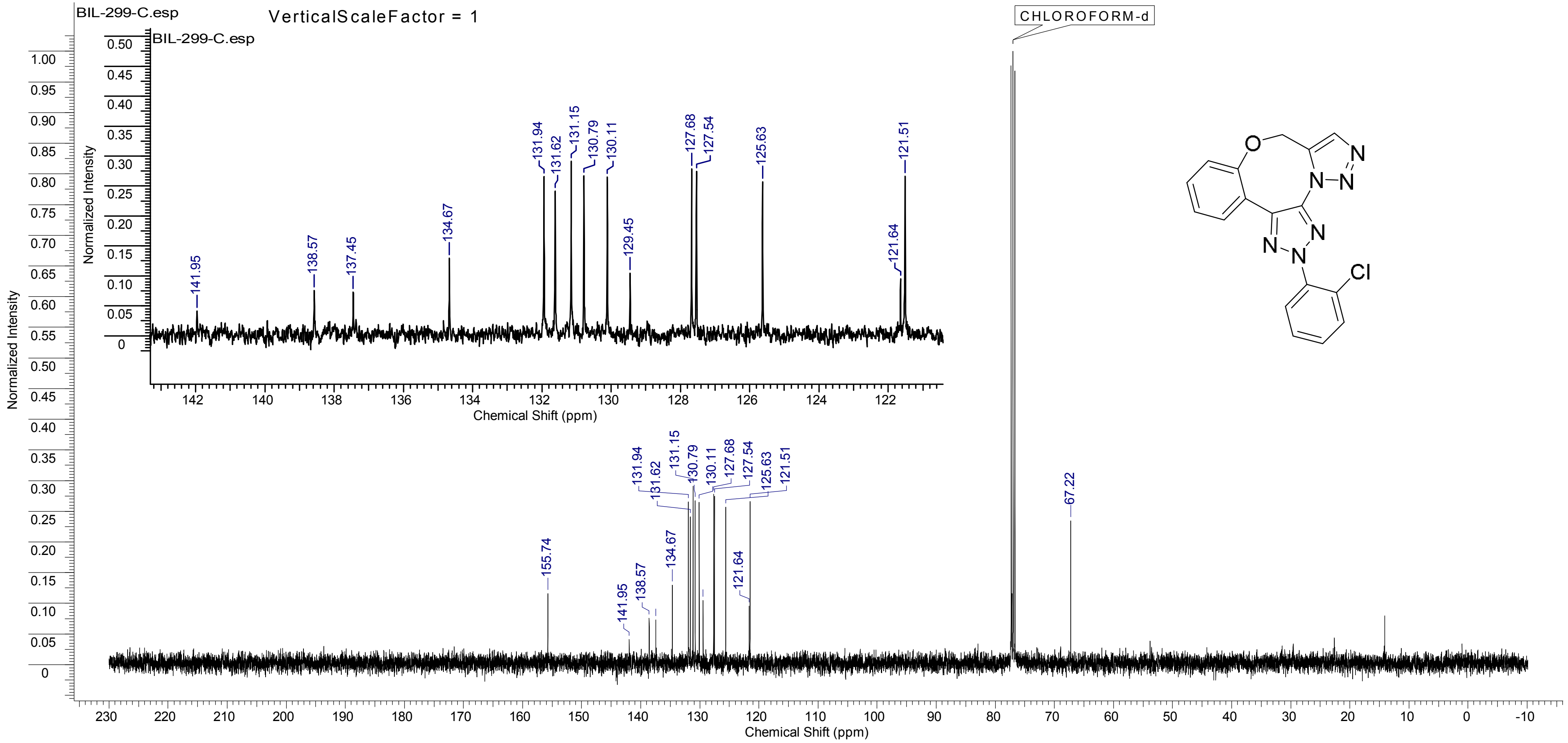

^{13}C NMR spectrum of **4j** (100.6 MHz, $CDCl_3$)

18.06.2019 22:40:24

Acquisition Time (sec)	4.0894	***Comment***	5 mm BBO BB-1H/D Z3918/0123			***Date***	17 Jun 2019 12:20:16
Date Stamp	17 Jun 2019 12:20:16						
File Name							
Frequency (MHz)	400.13	***Nucleus***	1H	***Number of Transients***	4	***Origin***	spect
Original Points Count	32768	***Owner***	root	***Points Count***	131072	***Pulse Sequence***	zg30
Receiver Gain	114.00	***SW(cyclical) (Hz)***	8012.82	***Solvent***	CHLOROFORM-d	***Spectrum Offset (Hz)***	2395.8870
Sweep Width (Hz)	8012.76	***Temperature (degree C)***	27.000				

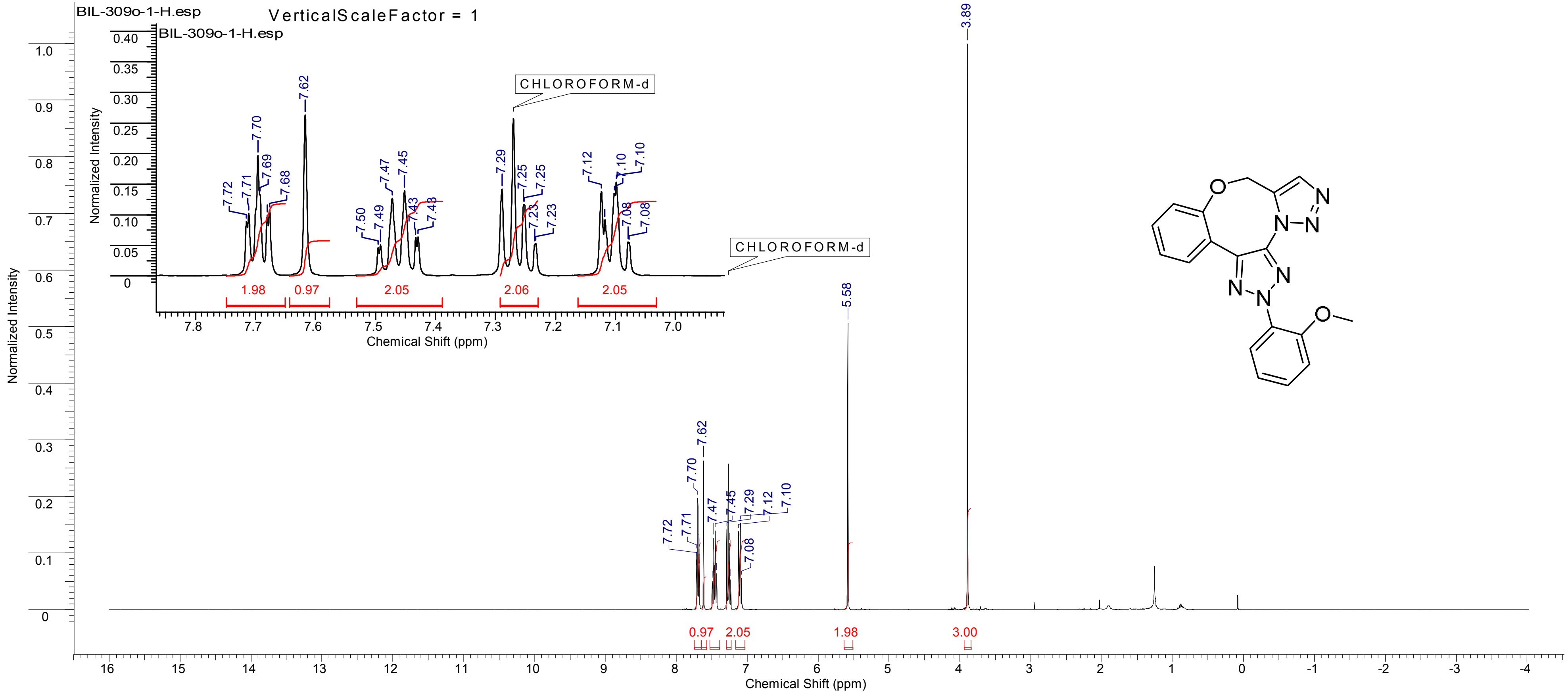

^{1}H NMR spectrum of **4k** (400.1 MHz, $CDCl_3$)

18.06.2019 22:36:06

**Acquisition Time (sec)**	0.6783	**Comment**	5 mm BBO BB-1H/D Z3918/0123			**Date**	17 Jun 2019 12:22:24
**Date Stamp**	17 Jun 2019 12:22:24						
**File Name**						**Frequency (MHz)**	100.61
**Nucleus**	13C	**Number of Transients**	65	**Origin**	spect	**Original Points Count**	16384
**Owner**	root	**Points Count**	131072	**Pulse Sequence**	zgpg30	**Receiver Gain**	9195.20
**SW(cyclical) (Hz)**	24154.59	**Solvent**	CHLOROFORM-d	**Spectrum Offset (Hz)**	11053.7178	**Sweep Width (Hz)**	24154.41
**Temperature (degree C)**	27.000						

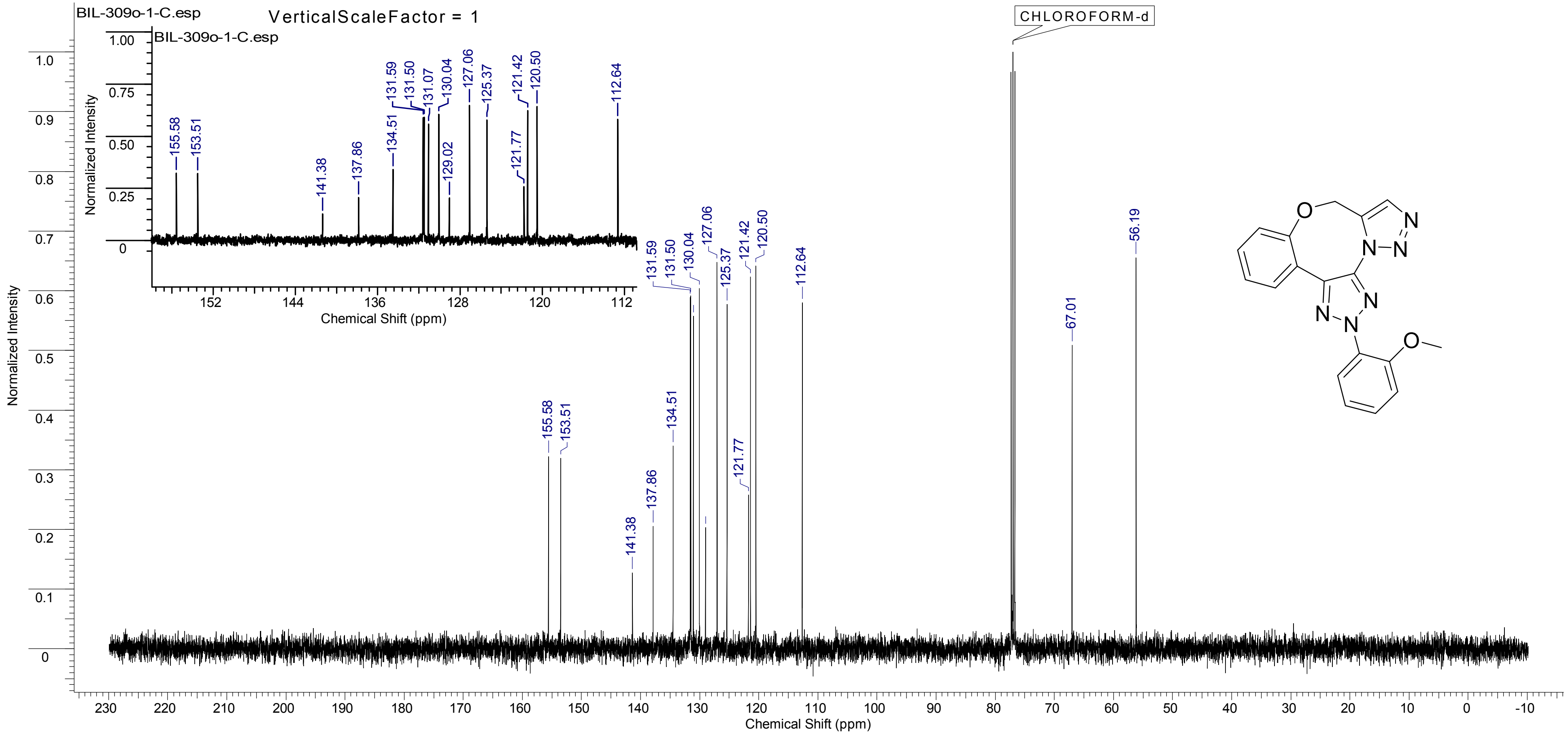

^{13}C NMR spectrum of **4k** (100.6 MHz, $CDCl_3$)

04.06.2019 14:09:05

**Acquisition Time (sec)**	4.0894	**Comment**	5 mm BBO BB-1H/D Z3918/0123			**Date**	25 May 2019 17:25:20
**Date Stamp**	25 May 2019 17:25:20						
**File Name**						**Frequency (MHz)**	400.13
**Nucleus**	1H	**Number of Transients**	4	**Origin**	spect	**Original Points Count**	32768
**Owner**	root	**Points Count**	131072	**Pulse Sequence**	zg30	**Receiver Gain**	203.20
**SW(cyclical) (Hz)**	8012.82	**Solvent**	CHLOROFORM-d	**Spectrum Offset (Hz)**	2395.7031	**Sweep Width (Hz)**	8012.76
**Temperature (degree C)**	27.000						

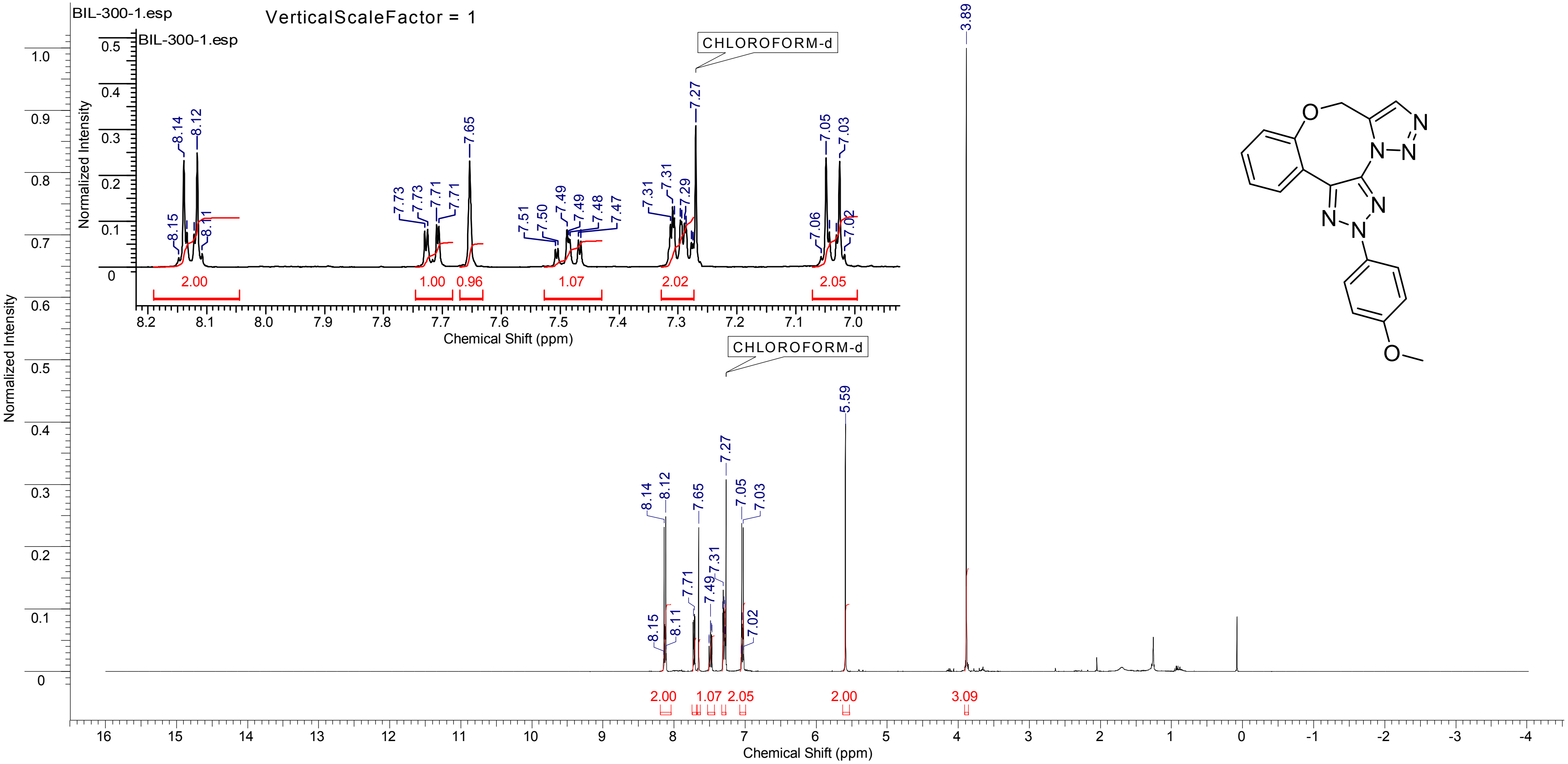

^{1}H NMR spectrum of **4l** (400.1 MHz, $CDCl_3$)

04.06.2019 14:14:45

Acquisition Time (sec)	0.6783	Comment	5 mm BBO BB-1H/D Z3918/0123			Date	27 May 2019 12:48:00
Date Stamp	27 May 2019 12:48:00						
File Name						Frequency (MHz)	100.61
Nucleus	13C	Number of Transients	117	Origin	spect	Original Points Count	16384
Owner	root	Points Count	131072	Pulse Sequence	zgpg30	Receiver Gain	13004.00
SW(cyclical) (Hz)	24154.59	Solvent	CHLOROFORM-d	Spectrum Offset (Hz)	11060.7207	Sweep Width (Hz)	24154.41
Temperature (degree C)	27.000						

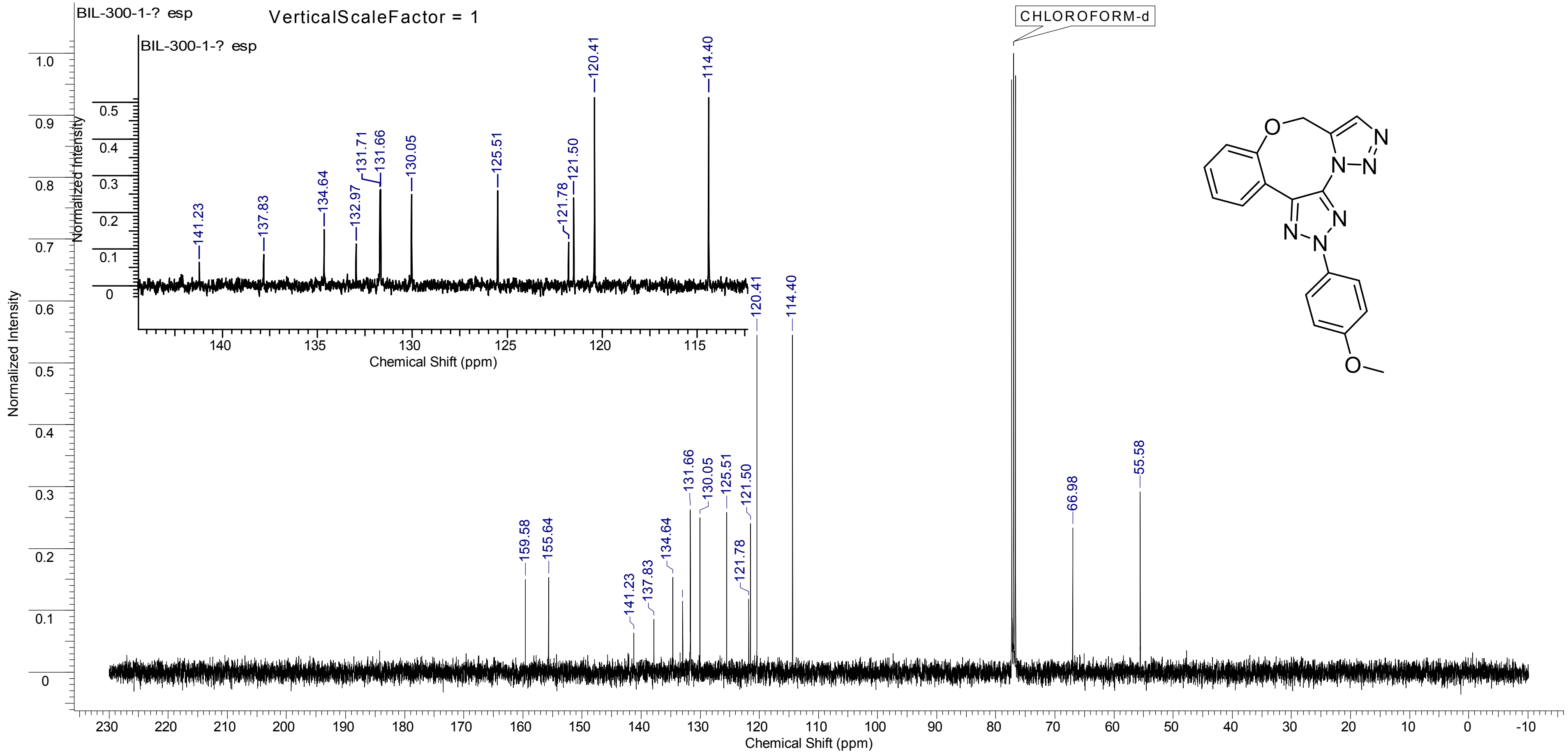

^{13}C NMR spectrum of **4l** (100.6 MHz, $CDCl_3$)

20.11.2019 12:47:00

Acquisition Time (sec)	4.0894	***Comment***	5 mm BBO BB-1H/D Z3918/0123			***Date***	16 Apr 2019 11:52:32
Date Stamp	16 Apr 2019 11:52:32						
File Name						***Frequency (MHz)***	400.13
Nucleus	1H	***Number of Transients***	4	***Origin***	spect	***Original Points Count***	32768
Owner	root	***Points Count***	131072	***Pulse Sequence***	zg30	***Receiver Gain***	203.20
SW(cyclical) (Hz)	8012.82	***Solvent***	CHLOROFORM-d			***Spectrum Offset (Hz)***	2395.6421
Sweep Width (Hz)	8012.76	***Temperature (degree C)***	27.000				

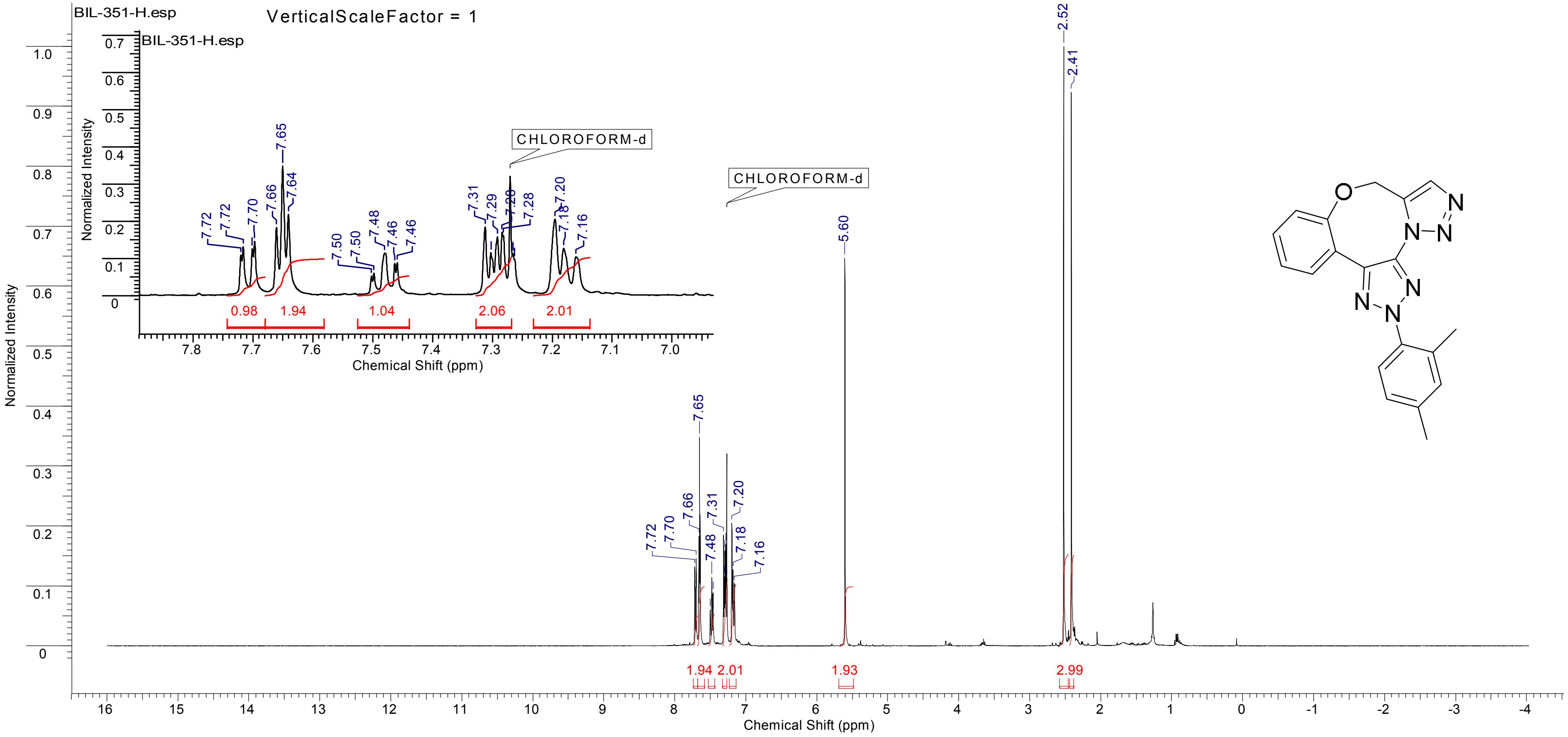

^{1}H NMR spectrum of **4m** (400.1 MHz, $CDCl_3$)

**Acquisition Time (sec)**	0.6783	**Comment**	5 mm BBO BB-1H/D Z3918/0123			**Date**	16 Apr 2019 11:52:32
**Date Stamp**	16 Apr 2019 11:52:32						
**File Name**						**Frequency (MHz)**	100.61
**Nucleus**	13C	**Number of Transients**	218	**Origin**	spect	**Original Points Count**	16384
**Owner**	root	**Points Count**	131072	**Pulse Sequence**	zgpg30	**Receiver Gain**	9195.20
**SW(cyclical) (Hz)**	24154.59	**Solvent**	CHLOROFORM-d	**Spectrum Offset (Hz)**	11061.8262	**Sweep Width (Hz)**	24154.41
**Temperature (degree C)**	27.000						

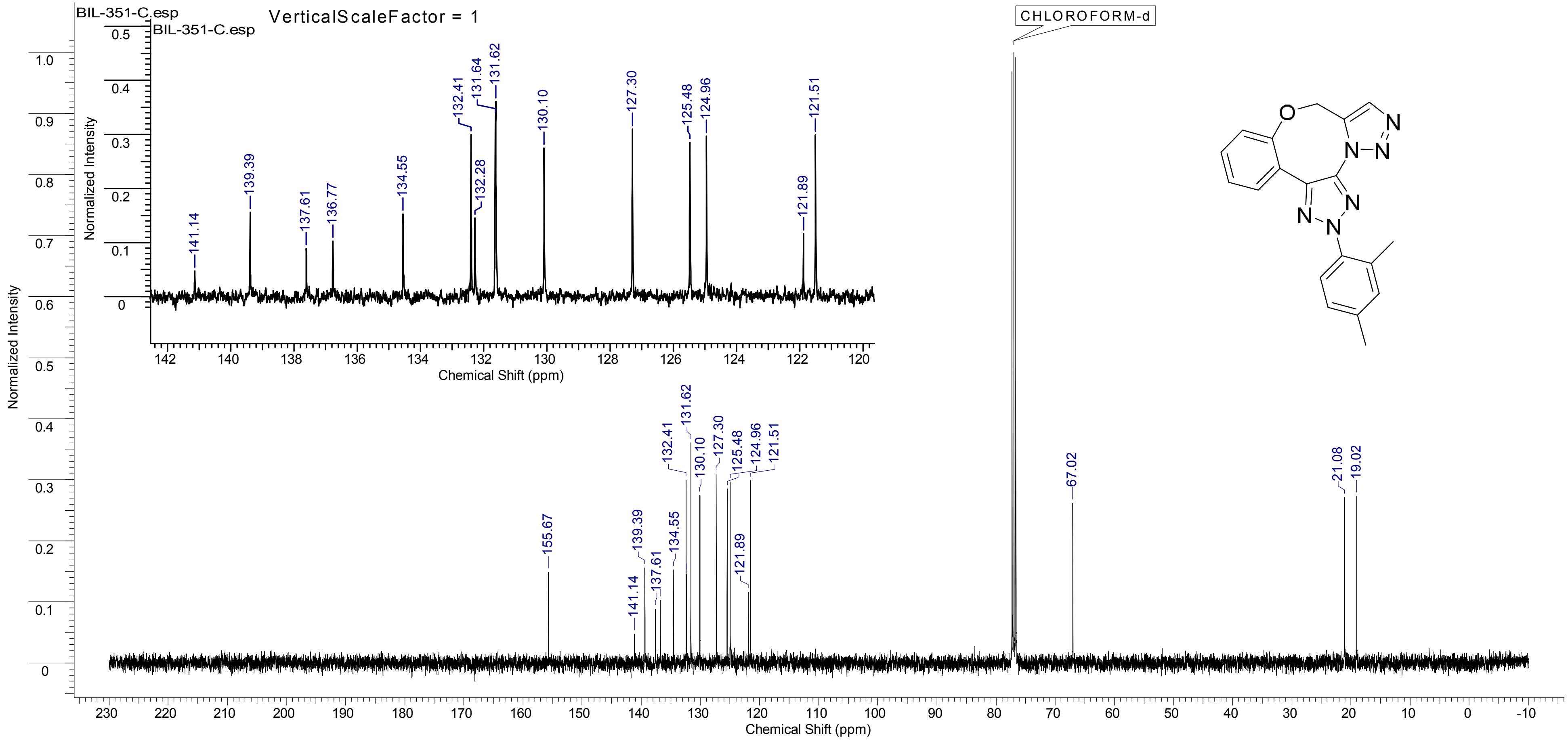

^{13}C NMR spectrum of **4m** (100.6 MHz, $CDCl_3$)

04.06.2019 18:53:43

**Acquisition Time (sec)**	2.5559	**Comment**	5 mm BBO BB-1H/D Z3918/0123			**Date**	01 Dec 2018 11:18:24		
**Date Stamp**	01 Dec 2018 11:18:24			**File Name**				**Frequency (MHz)**	400.13
**Nucleus**	1H	**Number of Transients**	4	**Origin**	spect	**Original Points Count**	16384	**Owner**	root
**Points Count**	65536	**Pulse Sequence**	zg30	**Receiver Gain**	161.30	**SW(cyclical) (Hz)**	6410.26	**Solvent**	CHLOROFORM-d
**Spectrum Offset (Hz)**	2595.7000	**Sweep Width (Hz)**	6410.16	**Temperature (degree C)**	27.000				

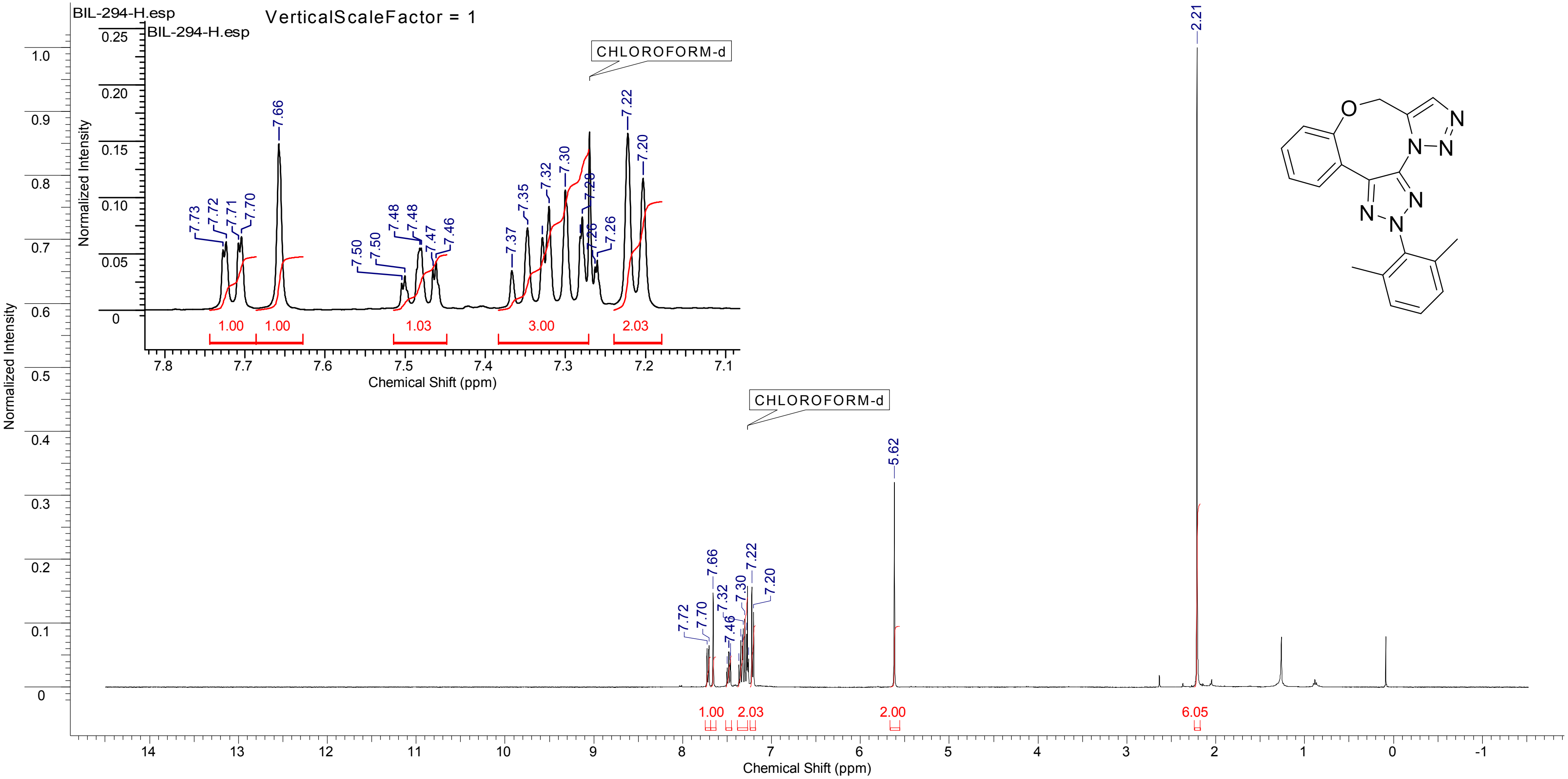

^{1}H NMR spectrum of **4n** (400.1 MHz, $CDCl_3$)

04.06.2019 18:54:49

Acquisition Time (sec)	0.6783	Comment	5 mm BBO BB-1H/D Z3918/0123	Date	01 Dec 2018 11:20:32		
Date Stamp	01 Dec 2018 11:20:32		File Name			Frequency (MHz)	100.61
Nucleus	13C	Number of Transients	76	Origin	spect	Original Points Count	16384
Owner	root						
Points Count	131072	Pulse Sequence	zgpg30	Receiver Gain	9195.20	SW(cyclical) (Hz)	24154.59
Solvent	CHLOROFORM-d			Spectrum Offset (Hz)	11058.1406	Sweep Width (Hz)	24154.41
Temperature (degree C)	27.000						

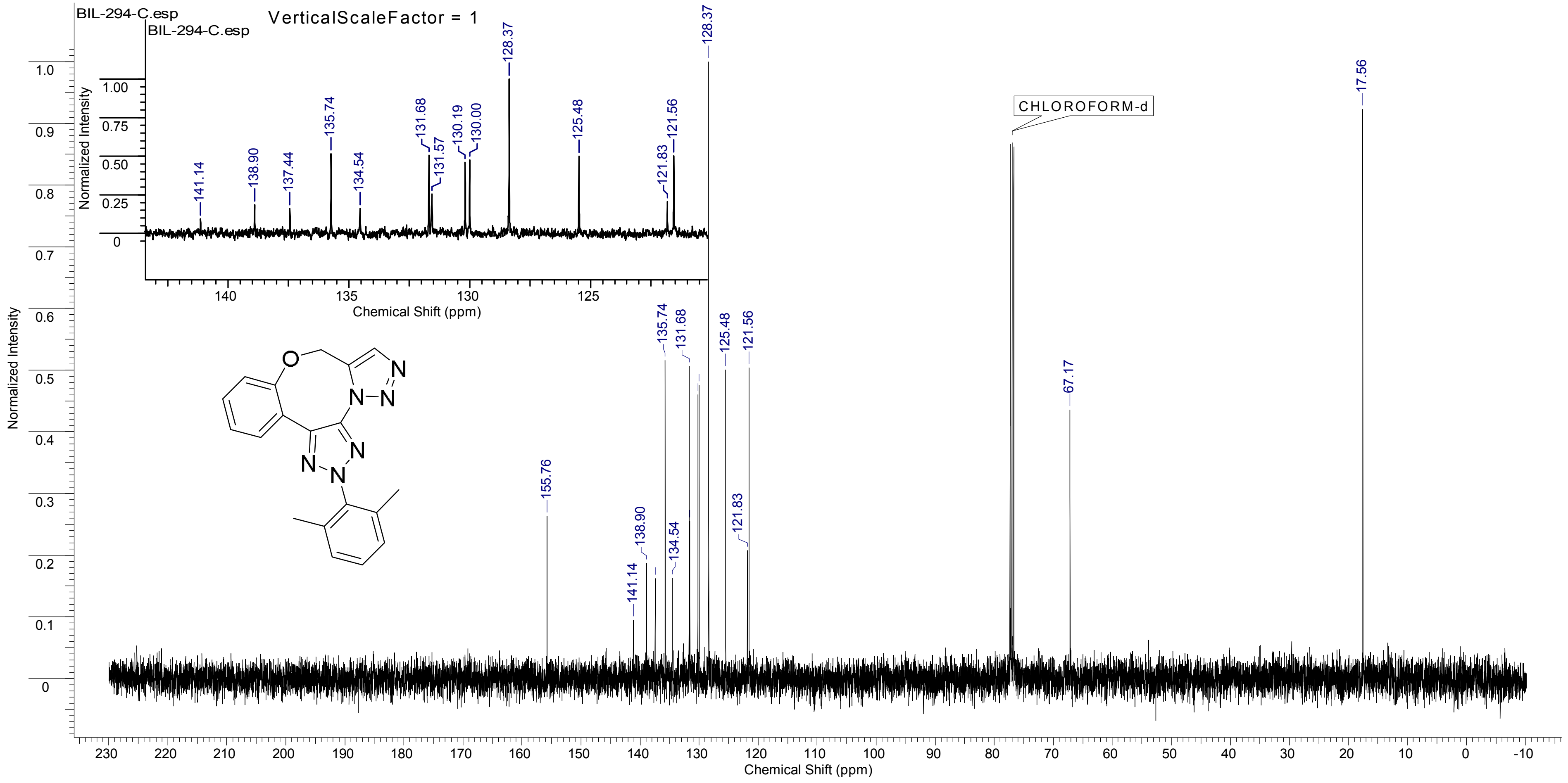

^{13}C NMR spectrum of **4n** (100.6 MHz, $CDCl_3$)

04.12.2019 0:26:08

Acquisition Time (sec)	4.0894	***Comment***	5 mm BBO BB-1H/D Z3918/0123			***Date***	13 Mar 2019 14:15:28
Date Stamp	13 Mar 2019 14:15:28						
File Name						***Frequency (MHz)***	400.13
Nucleus	1H	***Number of Transients***	4	***Origin***	spect	***Original Points Count***	32768
Owner	root	***Points Count***	131072	***Pulse Sequence***	zg30	***Receiver Gain***	161.30
SW(cyclical) (Hz)	8012.82	***Solvent***	CHLOROFORM-d			***Spectrum Offset (Hz)***	2395.8870
Sweep Width (Hz)	8012.76	***Temperature (degree C)***	27.000				

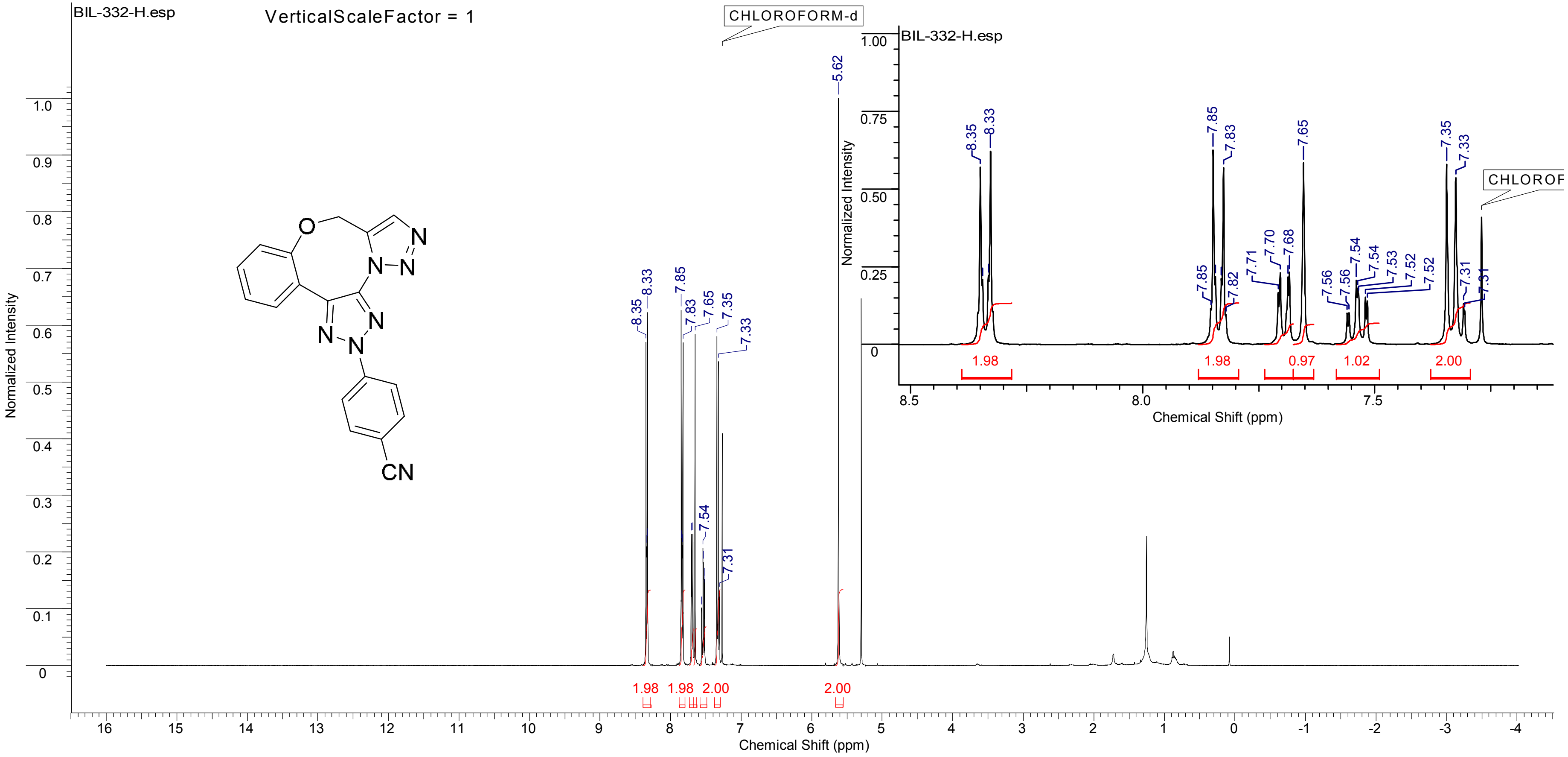

^{1}H NMR spectrum of **4o** (400.1 MHz, $CDCl_3$)

04.06.2019 19:24:21

**Acquisition Time (sec)**	0.6783	**Comment**	5 mm BBO BB-1H/D Z3918/0123			**Date**	13 Mar 2019 14:17:36
**Date Stamp**	13 Mar 2019 14:17:36						
**File Name**						**Frequency (MHz)**	100.61
**Nucleus**	13C	**Number of Transients**	73	**Origin**	spect	**Original Points Count**	16384
**Owner**	root	**Points Count**	131072	**Pulse Sequence**	zgpg30	**Receiver Gain**	16384.00
**SW(cyclical) (Hz)**	24154.59	**Solvent**	CHLOROFORM-d	**Spectrum Offset (Hz)**	11058.8779	**Sweep Width (Hz)**	24154.41
**Temperature (degree C)**	27.000						

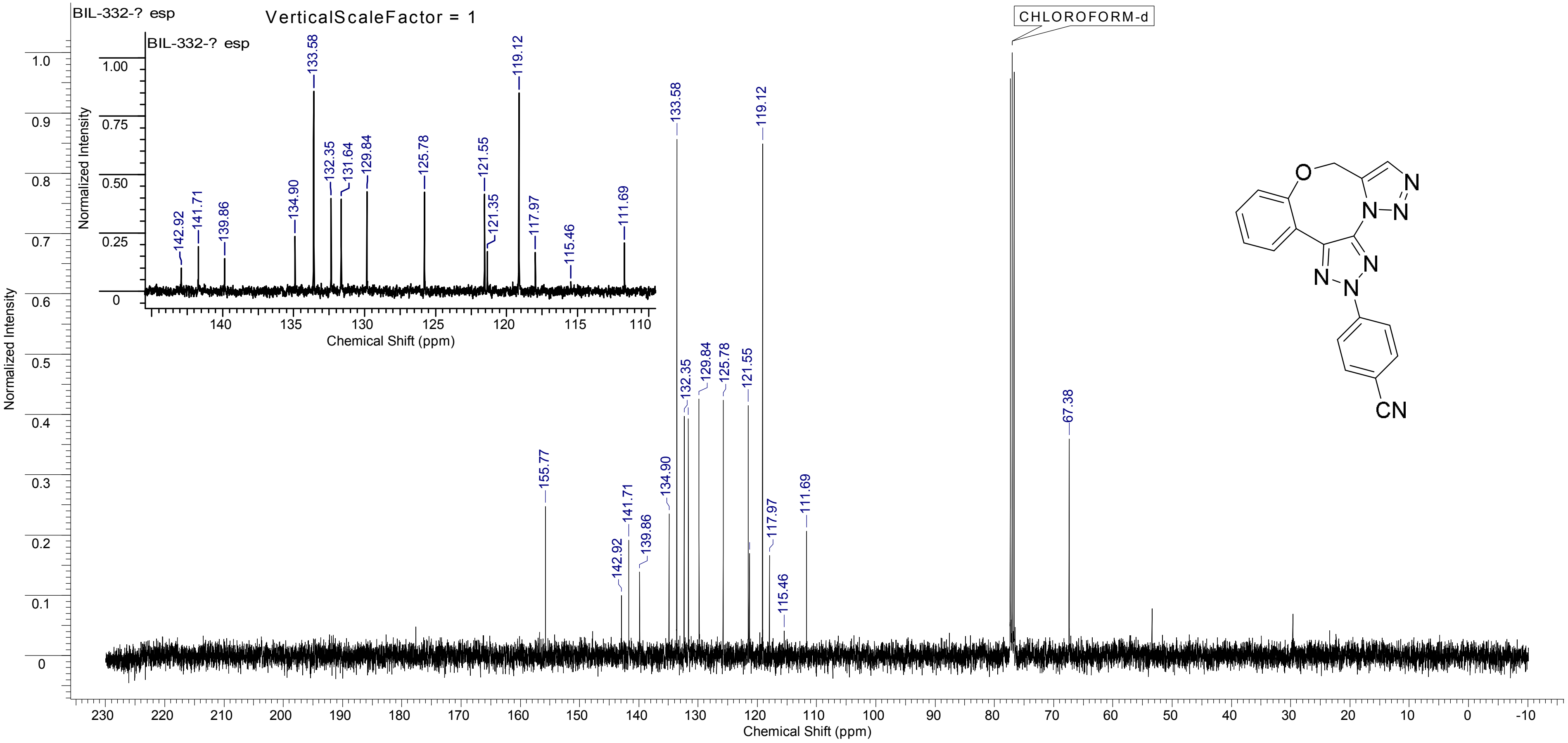

^{13}C NMR spectrum of **4o** (100.6 MHz, $CDCl_3$)
